# Von Hippel Lindau tumor suppressor controls m6A-dependent gene expression in renal tumorigenesis

**DOI:** 10.1172/JCI175703

**Published:** 2024-04-15

**Authors:** Cheng Zhang, Miaomiao Yu, Austin J. Hepperla, Zhao Zhang, Rishi Raj, Hua Zhong, Jin Zhou, Lianxin Hu, Jun Fang, Hongyi Liu, Qian Liang, Liwei Jia, Chengheng Liao, Sichuan Xi, Jeremy M. Simon, Kexin Xu, Zhijie Liu, Yunsun Nam, Payal Kapur, Qing Zhang

**Affiliations:** 1 Department of Pathology, University of Texas Southwestern Medical Center, Dallas, Texas, USA.; 2 Lineberger Comprehensive Cancer Center, University of North Carolina (UNC) School of Medicine, Chapel Hill, North Carolina, USA.; 3 Department of Genetics, Neuroscience Center and; 4 UNC Neuroscience Center, Carolina Institute for Developmental Disabilities, UNC, Chapel Hill, North Carolina, USA.; 5 Department of Molecular Medicine, Mays Cancer Center, University of Texas Health Science Center at San Antonio, San Antonio, Texas, USA.; 6 Department of Biochemistry, Department of Biophysics, Simmons Comprehensive Cancer Center and; 7 Department of Pathology, Department of Bioinformatics, University of Texas Southwestern Medical Center, Dallas, Texas, USA.; 8 Thoracic Epigenetics Section, Thoracic Surgery Branch, Center for Cancer Research, National Cancer Institute, National Institutes of Health, Bethesda, Maryland, USA.; 9 Kidney Cancer Program, Simmons Comprehensive Cancer Center, Department of Urology,; 10 Simmons Comprehensive Cancer Center, University of Texas Southwestern Medical Center, Dallas, Texas, USA.

**Keywords:** Oncology, Tumor suppressors

## Abstract

N6-Methyladenosine (m6A) is the most abundant posttranscriptional modification, and its contribution to cancer evolution has recently been appreciated. Renal cancer is the most common adult genitourinary cancer, approximately 85% of which is accounted for by the clear cell renal cell carcinoma (ccRCC) subtype characterized by VHL loss. However, it is unclear whether VHL loss in ccRCC affects m6A patterns. In this study, we demonstrate that VHL binds and promotes METTL3/METTL14 complex formation while VHL depletion suppresses m6A modification, which is distinctive from its canonical E3 ligase role. m6A RNA immunoprecipitation sequencing (RIP-Seq) coupled with RNA-Seq allows us to identify a selection of genes whose expression may be regulated by VHL-m6A signaling. Specifically, *PIK3R3* is identified to be a critical gene whose mRNA stability is regulated by VHL in a m6A-dependent but HIF-independent manner. Functionally, PIK3R3 depletion promotes renal cancer cell growth and orthotopic tumor growth while its overexpression leads to decreased tumorigenesis. Mechanistically, the VHL-m6A–regulated PIK3R3 suppresses tumor growth by restraining PI3K/AKT activity. Taken together, we propose a mechanism by which VHL regulates m6A through modulation of METTL3/METTL14 complex formation, thereby promoting *PIK3R3* mRNA stability and protein levels that are critical for regulating ccRCC tumorigenesis.

## Abstract 2

VHL regulates mRNA stability in an m6A-dependent but HIF-independent manner, which controls ccRCC tumorigenesis.

## Graphical abstract

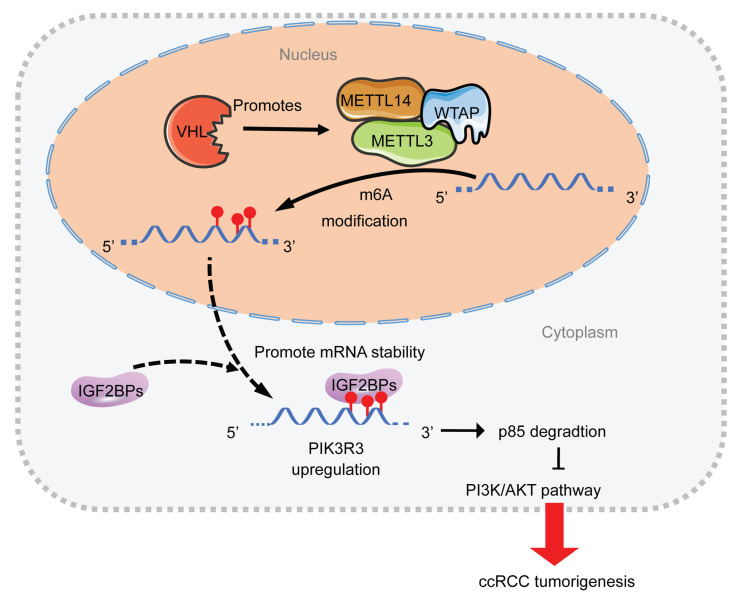

## Introduction

Estimated new cases and deaths from renal cancer in the US for 2020 were 73,750 and 14,830 respectively (1). Kidney cancer incidence has been steadily increasing over the past few decades, with unclear reasons for this trend (2). The *VHL* tumor suppressor gene was initially discovered as a germline mutation in patients at risk for clear cell renal cell carcinoma (ccRCC), which constitutes approximately 85% of all kidney cancers (3). Inactivating *VHL* mutations also play major roles in sporadic kidney cell cancer (4), and HIF signaling/activation emerges as a major effector downstream of VHL loss in ccRCC. The stabilization of HIF2-α, resulting from VHL loss, is both sufficient and necessary for promoting kidney tumor growth (5). Consequently, HIF2-α stands out as an important therapeutic target in *VHL* loss–related kidney cancer. Recent reports indicate that the specific HIF2-α inhibitor PT2399 (or highly related compound PT2385) effectively inhibits primary tumor growth and invasion in a subset of kidney cancers (6). However, approximately 50% of ccRCC cases exhibit resistance to HIF2-α inhibitor treatment (6), underscoring the importance of identifying additional therapeutic vulnerabilities for ccRCC.

Evidence suggests that the dysregulated N6-methyladenosine on RNA (m6A) is involved in the initiation and progression of renal cancers (7), suggesting a potential therapeutic opportunity for ccRCC. The m6A represents a most prevalent modification of mRNA in higher eukaryotes (8). In mammals, installation of the methyl group onto selective adenosine is catalyzed by a methyltransferase complex that comprises METTL3, METTL14, and WTAP (9). The methylation can be removed by 2 RNA demethylases, FTO and ALKBH5 (9). Reader proteins like YT521-B homology (YTH) domain–containing proteins and IGF2BP proteins are crucial for mRNA decay, translation, and stability, with YTH proteins influencing decay or translation, and IGF2BP proteins enhancing stability and translation of target mRNAs. YTH domain–containing proteins are responsible for mRNA decay or translation, while IGF2BP proteins promote the stability and translation of most of their target mRNAs (10). Multiple studies underscore the significance of m6A in renal cancer: (a) the m6A writer proteins METTL3 and METTl14 have been identified as powerful prognostic factors in ccRCC (11); (b) the m6A demethylase FTO is a synthetic lethal partner of VHL in ccRCC, and aberrant of FTO in ccRCC sensitizes these tumors to BRD9 inhibitors (12, 13); and (c) the reader protein IGF2BP3 promotes ccRCC tumor growth by stabilizing target genes in an m6A-dependent manner (14). However, the molecular mechanism underlying the role of m6A in ccRCC tumorigenesis and aggression is still not well understood. Moreover, in the ccRCC, which is characterized by the inactivation of VHL tumor suppressor gene, it is largely unclear how genetic alterations involving VHL would affect m6A in ccRCC.

The phosphoinositide 3 kinase (PI3K) pathway has been linked closely with kidney cancer, characterized by hyperactivation of PI3K and AKT signaling (15). Various types of cancer, including renal cancer, show a connection between altered PI3K/AKT pathway activation and changes in m6A mRNA methylation or its regulators (16). In renal cancer cells, overexpression of METTL3 leads to decreased PI3K/AKT activation as well as suppressed tumor growth (17), suggesting a PI3K/Akt-dependent regulatory function of m6A modification in ccRCC. However, it remains unclear the regulation mechanism of m6A on PI3K/AKT pathway in ccRCC. As a regulator subunit of class I PI3K complex, PIK3R3 has been suggested to play pivotal roles in different physiological and pathologic processes including cell proliferation, inflammation, epithelial-mesenchymal transition (EMT) and tumor growth (18–20). Interestingly, survival analysis of samples of patients with kidney renal clear cell carcinoma (KIRC) from the cancer genome atlas (TCGA) data set shows a favorable role of PIK3R3 on survival of patients with KIRC. However, the detailed function and mechanism of PIK3R3 in KIRC remain unexplored. In this study, we establish the role of VHL on the regulation of m6A, especially on PIK3R3, which leads to dysregulation of PI3K/AKT signaling.

## Results

### VHL binds with m6A enzymatic complex proteins.

**Figure 1:**
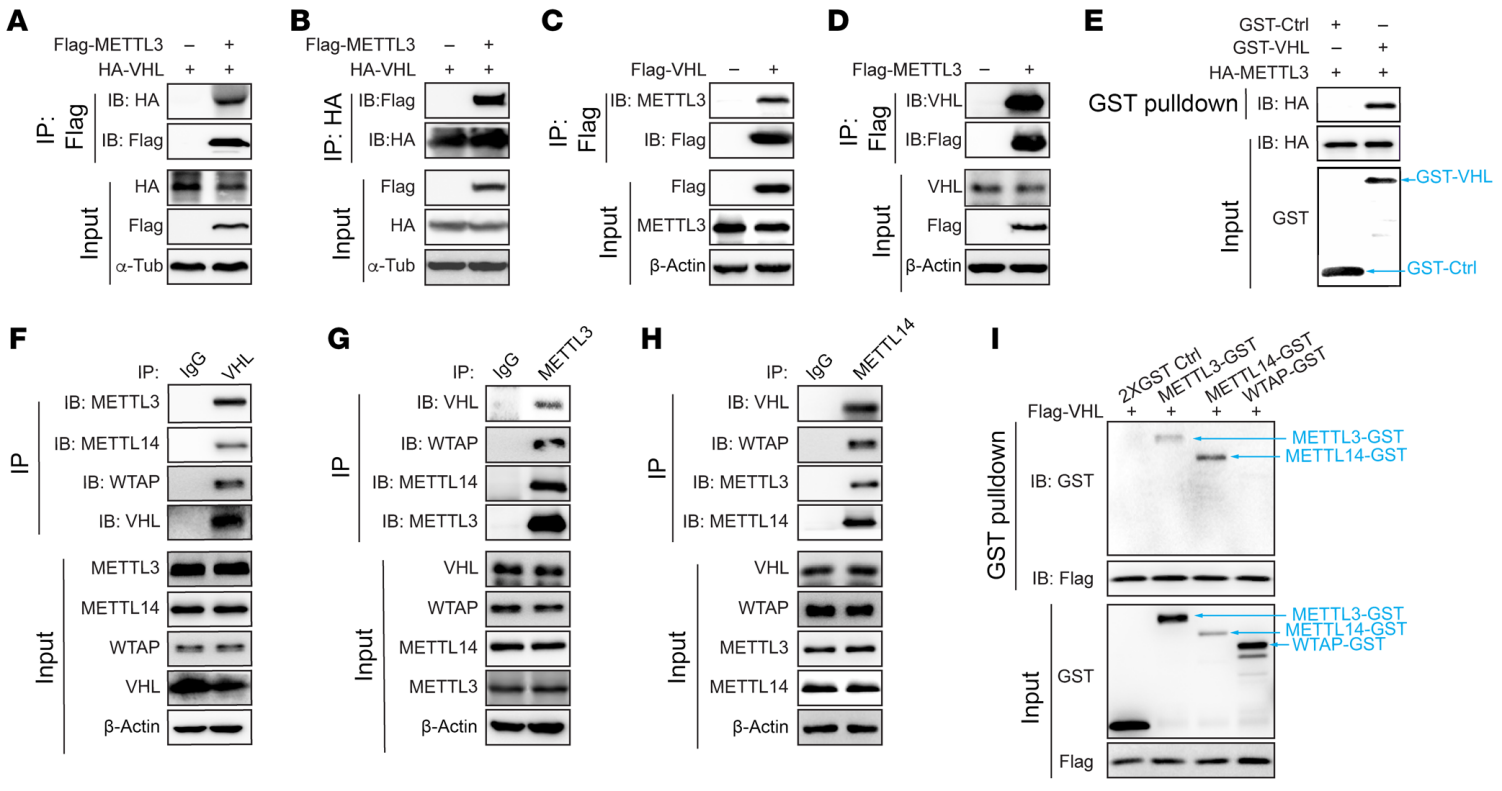
VHL interacts with m6A writer proteins. (**A**–**D**) Anti-Flag or HA immunoprecipitation with the samples from 293T cells transfected with indicated vectors. (**E**) GST-pull down assay with purified GST, GST-VHL and in vitro translated (IVT) HA-METTL3. (**F**–**H**) Endogenous immunoprecipitation assay with indicated antibodies. METTL3, METTL14, and WTAP blots were run in parallel using the same biological samples. (**I**) Anti-Flag immunoprecipitation with IVT Flag-VHL and GST tagged proteins (2XGST, METTL3-GST, METTL14-GST, WTAP-GST).

A previous report showed that VHL could potentially regulate mRNA stabilization of the survival factor parathyroid hormone-related protein in human renal cell carcinoma (21). In addition, the role of m6A regulatory proteins has been reported to be potentially important in ccRCC (22). However, the potential role of VHL in regulating m6A and RNA stability has not been reported. As VHL is the most important tumor suppressor in ccRCC, we aimed to determine whether VHL could potentially interact with m6A enzymatic complex proteins. To this end, we performed coimmunoprecipitation (co-IP) assays and found that VHL interacted with METTL3, either exogenously, semiendogenously in 293T cells, or in the pure in vitro conditions (Figure 1, A–E). To determine whether VHL could potentially interact with other m6A complex proteins, such as METTL14 or WTAP, we also performed VHL endogenous immunoprecipitation and found that VHL interacted with METTL3, METTL14, and WTAP (Figure 1F). In HKC cells, endogenous VHL was able to interact with METTL3 and METTL14 but not WTAP (Supplemental Figure 1A; supplemental material available online with this article; https://doi.org/10.1172/JCI175703DS1). In addition, reciprocal immunoprecipitations for METTL3 or METTL14 revealed that these proteins interacted with VHL endogenously (Figure 1, G and H), further supporting the potential connection between VHL and m6A regulation. GST-pull down assay with in vitro–expressed METTL3, METTL14, WTAP, and VHL revealed the interaction between VHL and METTL3 or METTL14 (Figure 1I). It should be noted that the in vitro pull-down assay is performed in the presence of RNase to exclude the bridge effect of RNA on protein-protein interaction (Supplemental Figure 1B). Our results suggested that METTL3 and METTL14 interacted with VHL directly. Since VHL is an E3 ligase, we examined whether VHL-METTL3 interaction depends on its E3 ligase activity. Interestingly, co-IP of VHL with METTL3 or METTL14 showed that, in contrast to either VHL WT or other truncation mutants, the VHL E3 ligase deficient mutant (VHL^Δ92–121^) failed to interact with METTL3 or METTL14 (Supplemental Figure 1, C and D). Our results suggest that the VHL E3 ligase domain may be important for regulating METTL3/14 complex formation.

### VHL regulates m6A levels in ccRCC cells.

**Figure 2:**
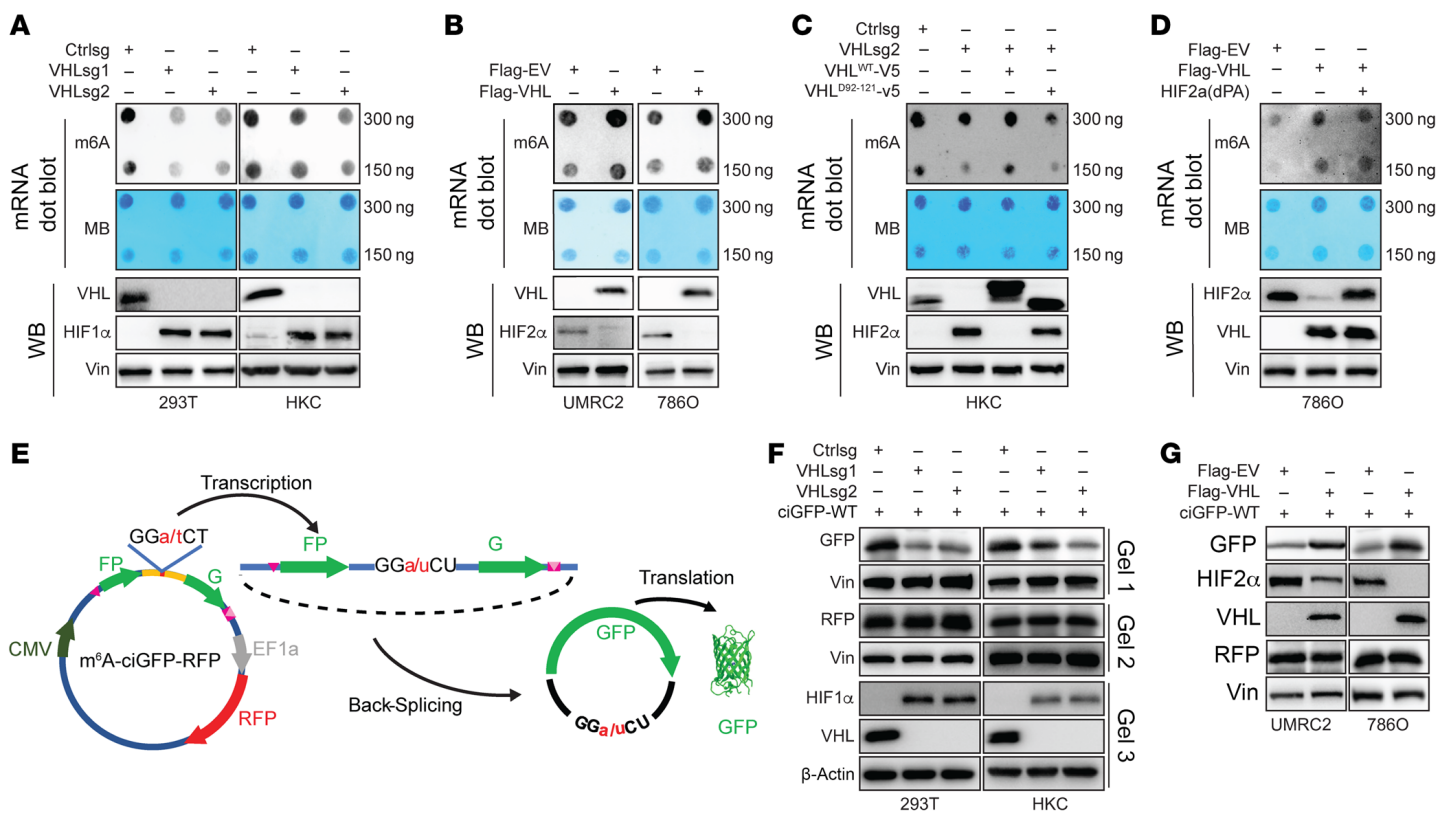
VHL regulates m6A modification in ccRCC. (**A** and **B**) The m6A level of poly(A) + RNAs (mRNAs) isolated from VHL knockout (KO) cells (**A**) or VHL-overexpressing ccRCC cells (**B**) were indicated by m6A dot blot. Corresponding RNAs were loaded equally by a 2-fold serial dilution of 600 ng and 300 ng. Methylene blue staining served as a loading control. Western blots show the knockout efficiency of indicated sgRNAs or overexpressing levels of exogenous VHL. HIF1-α or HIF2-α were used as a positive control of VHL KO. (**C** and **D**) m6A dot of mRNAs and immunoblot of cell lysate from the cells transfected with indicated plasmids. (**E**) Schematic diagram of a circular RNA (circRNA) translation reporter consisting of a single exon and 2 introns with complementary sequences. The exon can be back-spliced to generate circRNAs that drive GFP translation from the GGACU motif. (**F** and **G**) Immunoblotting analysis of indicated GFP expressing level in cells that transfected with indicated vectors. GFP, RFP, and VHL blots were run in parallel using the same biological samples.

Since VHL binds directly with the catalytic subunit of m6A enzymatic complexes, we were interested to see whether VHL may regulate m6A levels in kidney cells. To this end, first we depleted *VHL* with 2 different sgRNAs in HKC and 293T cells and performed dot blots to examine gross m6A levels. VHL depletion led to decreased m6A levels in both cell lines (Figure 2A). Because ccRCC is characterized by loss of VHL, we reintroduced VHL in 2 different VHL-null ccRCC cell lines (786O and UMRC2) and observed increased m6A in these cells (Figure 2B). In addition, in ccRCC patient–derived tumorgraft (PDX) cell lines that are VHL null, upon overexpression of VHL, we observed increased m6A modification (Supplemental Figure 2A). This suggests that the function of VHL on m6A modification is generalizable to patients with ccRCC. To examine whether the effect of VHL depletion on m6A is due to the on-target effect of *VHL* sgRNAs, we performed rescue experiments in VHL-depleted cells by reintroducing a sgRNA-resistant VHL construct and found that VHL restoration rescued the m6A depletion effect. On the other hand, the VHL E3 ligase–deficient mutant failed to do so, arguing that the effect of VHL on m6A depends on its E3 ligase activity (Figure 2C). Since HIF is a canonical VHL E3 ligase substrate, we aimed to determine whether HIF is essential for mediating the effect of VHL on m6A regulation. We overexpressed a HIF2-α variant HIF2-α(dPA), in which its 2 prolyl hydroxylation sites have been changed to alanine and are therefore resistant to VHL recognition and degradation. In 786O cells, which only express HIF2-α but not HIF1-α (23), VHL overexpression leads to increased m6A modification. However, HIF2-α (dPA) overexpression could not reverse the effects of VHL on m6A modification (Figure 2D). In addition, HIF2-α depletion in 786O cells didn’t cause significant change to m6A modification (Supplemental Figure 2B).

Next, we employed a circular RNA GFP reporter containing a consensus m6A GGACU motif in HKC cells (Figure 2E). The GFP pre-mRNA transcript was assembled by backsplicing to generate a circular RNA that joins 2 exon fragments of GFP. m6A methylation of the GGACU motifs on the circular RNA can drive translation initiation of the GFP transcript (24), therefore generating GFP signal that can be detected by either Western blots or immunofluorescence. As a result, the intensity of GFP can be used as a direct readout of m6A methylation level in the cells. In addition, this reporter contains an endogenous constitutively expressed RFP that can serve as the control. The usage of this reporter system has been recently published (24). As a proof-of-principle, we showed that GFP protein levels were higher in the m6A WT motif (GGACU) reporter cells than the mutant (GGUCU), and depletion of METTL3 by shRNAs led to decreased GFP protein levels in HKC cells, while not affecting RFP (Supplemental Figure 2, C and D), suggesting that this reporter system can faithfully detect m6A levels in these cells. Next, we depleted *VHL* by sgRNAs in HKC and 293T cells and found that abrogation of VHL in these cells led to decreased GFP (Figure 2F), indicating decreased m6A. Conversely, VHL overexpression in VHL-null 786O and UMRC2 ccRCC cells led to increased GFP (Figure 2G). Our results suggest that VHL positively regulates m6A in renal cells.

### VHL regulates the interaction between METTL3 and METTL14.

**Figure 3:**
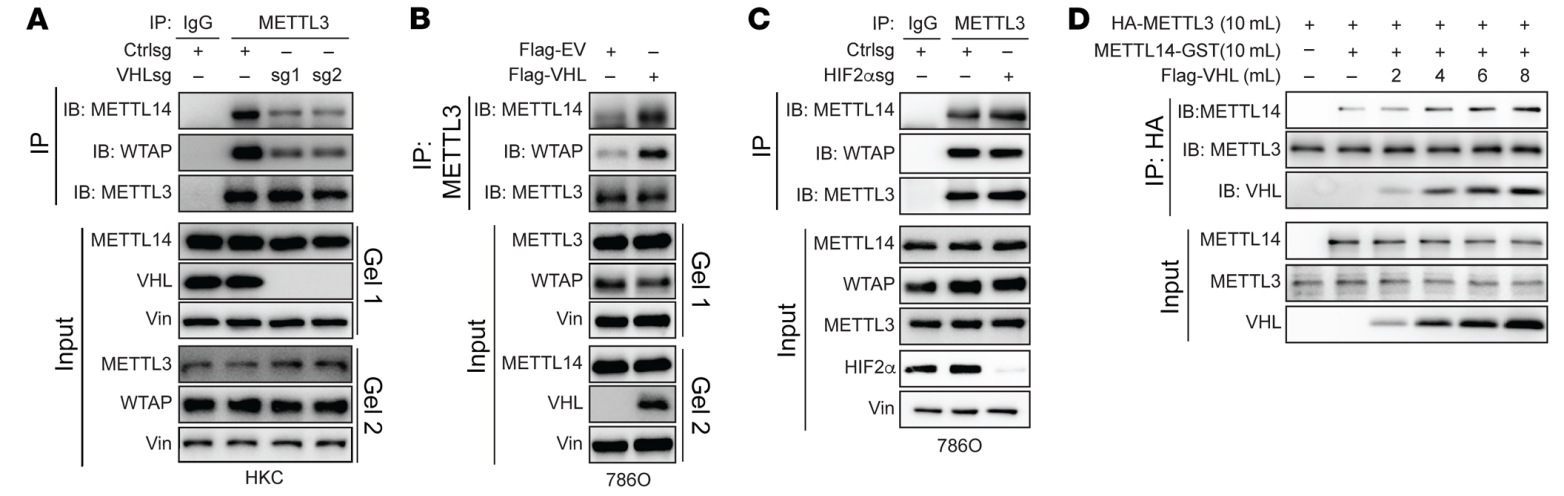
VHL regulates the interaction between METTL3 and METTL14. (**A**–**C**) METTL3 immunoprecipitation with samples from cells that transfected with indicated vectors. METTL3, METTL14, and WTAP blots were run in parallel using the same biological samples. (**D**) Anti-HA immunoprecipitation with IVT HA-METTL3, Flag-VHL, and METTL14-GST. METTL3 and METTL14 blots were run in parallel using the same biological samples.

We aimed to determine the molecular mechanism by which VHL regulates m6A. First, we examined whether VHL regulates protein levels of m6A writer complex proteins, including METTL3, 14, and WTAP. Neither VHL depletion (by sgRNAs) nor VHL overexpression affected these protein levels (Supplemental Figure 3, A and B). In addition, we also examined cellular localization of these proteins but did not observe any significant change on the localization of these proteins upon VHL manipulation (Supplemental Figure 3, C and D). Next, we examined the interaction of m6A writer protein complexes. To this end, first we depleted VHL by 2 independent sgRNAs and found that VHL depletion led to decreased binding between METTL3 and METTL14 (Figure 3A). Conversely, VHL overexpression in VHL-null 786O and UMRC2 ccRCC cells led to increased METTL3/METTL14 binding (Figure 3B and Supplemental Figure 3E). To further determine whether HIF signaling is important on METTL3/14 interaction, we also depleted HIFs and found that METTL3/14 interaction was not affected (Figure 3C and Supplemental Figure 3F). To examine whether VHL can directly affect their binding, we overexpressed various amounts of VHL in an in vitro translation system and found that VHL promoted METTL3/METTL14 in a dose-dependent manner (Figure 3D). However, in vitro methylation activity assays did not show increased METTL3/METTL14 complex activity with increased amounts of VHL, suggesting that the effect of VHL on METTL3/14 complex activity may not be direct but rather dependent on the cellular context (Supplemental Figure 3G).

### Identification of VHL-regulated m6A sites by MeRIP-Seq.

**Figure 4:**
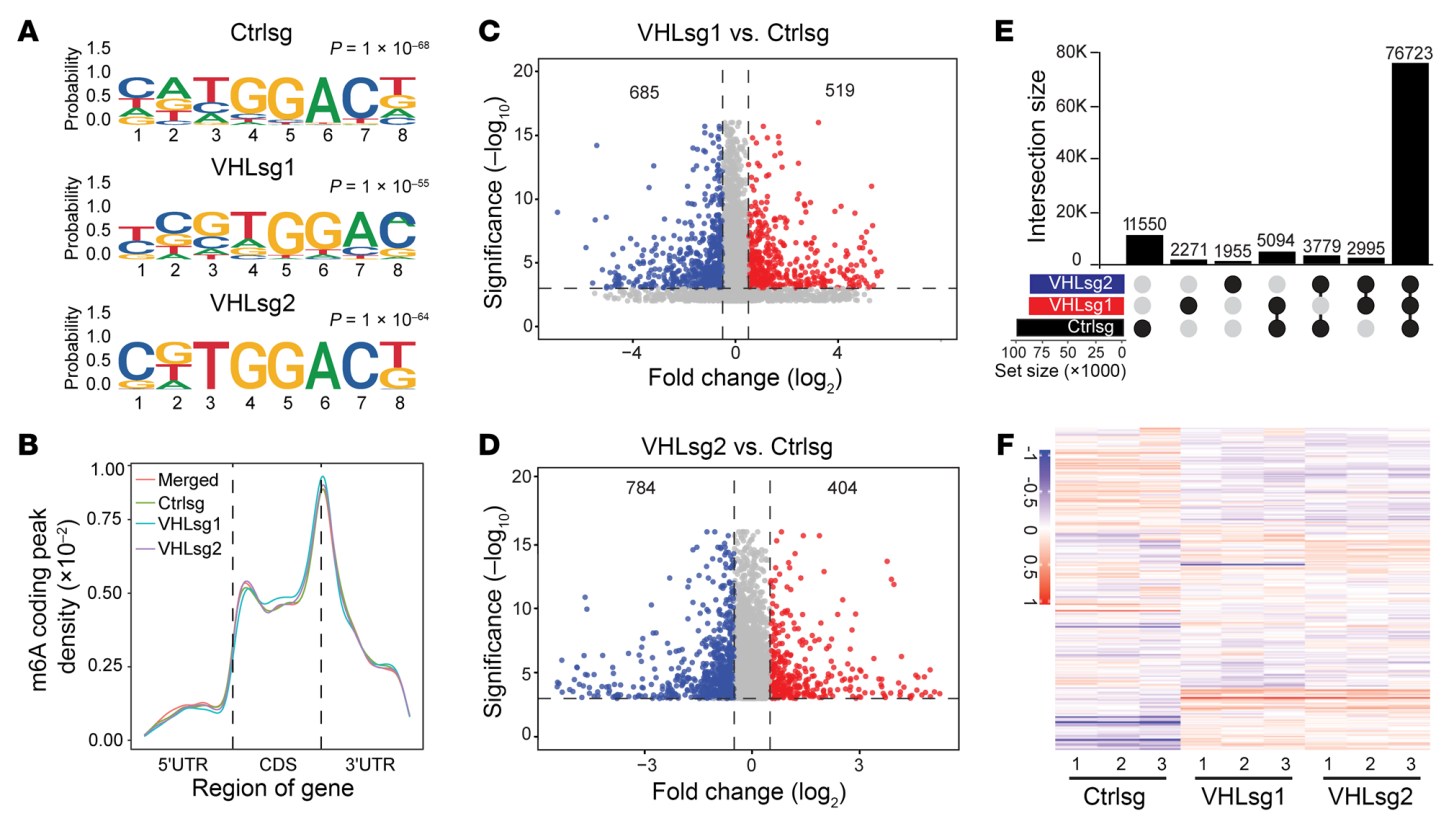
Transcriptome-wide identification of different m6A upon VHL depletion. (**A**) Top consensus sequences of the m6A motif detected by HOMER motif analysis with MeRIP-Seq data. (**B**) m6A distribution pattern across mRNA transcriptome. (**C** and **D**) Volcano plots show the different m6A peaks between Ctrlsg and *VHL*sg1 (**C**) or *VHL*sg2 (**D**). (**E**) Upset plot show the m6A modification sites number in different groups (Ctrlsg, *VHL*sg1, and *VHL*sg2). (**F**) Heatmap of differentially expressed genes (DEGs) identified by RNA-Seq.

Motivated by our finding with the effect of VHL on m6A enzymatic complex binding, especially on the binding between METTL3 and METTL14, we aimed to examine potential downstream target genes regulated by VHL involving m6A modification. It is important to point out that, to our knowledge, this has not been investigated in renal cancer before. To this end, we purified mRNA samples followed by m6A RNA immunoprecipitation (MeRIP). Quantitative PCR (qPCR) test results demonstrated that the m6A antibody pull-down efficiently enriched m6A-positive regions but displayed no enrichment for negative regions, suggesting specific MeRIP enrichment in these cells (Supplemental Figure 4A). We then performed high-throughput sequencing and investigated the sequence features of m6A methylation sites across the genome. We analyzed the motifs enriched by m6A antibody pull down and found that the canonical m6A motif sequence GGAC was retrieved from all cell lines (Ctrlsg, *VHL*sg1, or *VHL*sg2) (Figure 4A). In addition, most m6A peaks were enriched in the region close to 3′ UTR, which is consistent with previous publications (25) on the genomic localization of m6A (Figure 4B). Mapping of transcripts with m6A modification revealed that m6A methylation was globally decreased from the transcriptome of VHL-KO cells (Supplemental Figure 4B) Differential RNA methylation analysis resulted in 685 downregulated and 519 upregulated (*VHL*sg1 versus Ctrlsg) or 784 downregulated and 404 upregulated (*VHL*sg2 versus Ctrlsg) m6A peaks (Figure 4, C and D). We also employed a convolutional neural network model (MTAK, http://matk.renlab.org/#/home) to identify m6A sites in these cell lines and found that VHL depletion led to decreased m6A site numbers, which is consistent with the observation that VHL depletion led to decreased METTL3-METTL14 binding (97,146 sites in control versus 87,083 and 85,451 in *VHL* sgRNA [sg1, sg2], respectively) (Supplemental Figure 4C). Next, we enumerated the unique m6A sites that only appeared in control sgRNA, but not in *VHL* sgRNAs (sg1, sg2) and retrieved 11,550 sites, corresponding to 3,975 genes (Figure 4E).

### Identification of VHL-regulated mRNA by integrated analysis of m6A RIP-Seq and RNA-Seq.

**Figure 5:**
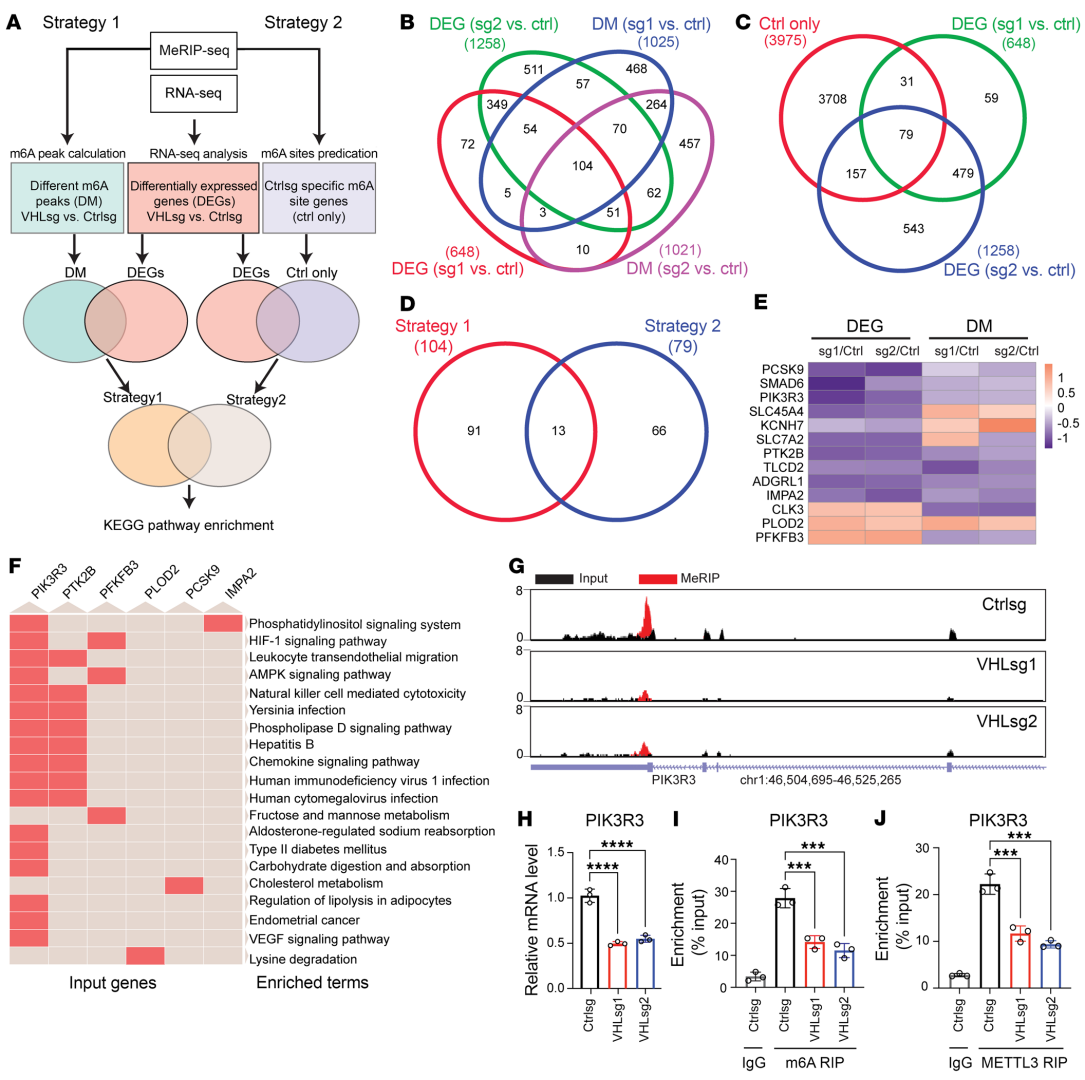
Transcriptome-wide RNA-Seq and m6A-Seq assays identify potential targets of VHL involved m6A modification. (**A**) Schematic flow chart demonstrating the framework to identify VHL-involved m6A targets. (**B**) Overlapping analysis of genes identified by different m6A peaks (DM) and differentially expressed genes (DEGs). (**C**) Overlapping analysis of genes identified by Ctrl only m6A site genes and DEGs. (**D**) Overlapping analysis of top enriched genes by strategy 1 and strategy 2. (**E**) Heatmap of DEGs and different m6A peaks (DM) of top enriched genes. (**F**) Clustergram of top enriched genes in KEGG pathways. (**G**) Genome browser representative tracks of 3 biological replicates displaying the m6A read distribution and changes (based on the m6A MeRIP-Seq and RNA-Seq data) in *PIK3R3* transcripts upon the KO of VHL in HKC cells. (**H**) qPCR analysis of *PIK3R3* mRNA levels in HKC Ctrlsg and VHLsg cells. (**I**) MeRIP-qPCR analysis of *PIK3R3* m6A levels in HKC Ctrlsg and VHLsg cells. (**J**) The association of METTL3 with *PIK3R3* mRNA was assessed by METTL3 RIP-qPCR analysis. Data show mean ± SD, ***P < 0.001, ****P < 0.0001, 1-way ANOVA analysis.

In order to examine gene expression that may be regulated by m6A modification, we performed RNA-Seq for these cell lines and focused on genes differentially regulated following depletion of VHL (Figure 4F). To identify the m6A targets downstream of VHL, 2 complementary strategies were used for target gene identification (Figure 5A). With the first strategy, we chose genes that displayed differential m6A enrichment (DM, |Log_2_FC| > 0.5, *P* < 0.001) and differential expression (DEGs, |Log_2_FC| > 0.5, *P* < 0.01) between control and *VHL* sgRNAs (Figure 5B). With the second strategy, we chose genes that displayed m6A modification sites only in control cells with DEGs (|Log_2_FC| > 0.5, *P* < 0.01) (Figure 5C). By combining and overlapping these 2 target gene lists, we obtained a short list of 13 genes, in which we were able to validate their differential gene expression and differential m6A enrichment (Figure 5, D and E). By performing KEGG pathway analysis, the present frequency of these 13 genes in the top listed pathways was ranked (Figure 5F). Next, we examined m6A occupancy on the top 3 genes. VHL depletion led to decreased m6A occupancy in 3′ UTR regions of *PIK3R3, PTK2B,* and *PFKFB3* (Figure 5G and Supplemental Figure 5A) as well as decreased RNA levels (Figure 5H and Supplemental Figure 5B), consistent with the RNA-Seq results (Supplemental Figure 5C). To confirm these findings from m6A RIP-Seq, we also designed primers and performed m6A RIP-PCR for these m6A-occupied regions. Our results demonstrated that VHL depletion led to decreased m6A occupancy on *PIK3R3*, *PTK2B*, and *PFKFB3* 3′ UTR regions (Figure 5I and Supplemental Figure 5D). In addition, VHL knockout led to decreased METTL3 occupancy on those genes (Figure 5J, Supplemental Figure 5E).

### PIK3R3 mRNA stability is regulated by VHL in an m6A and IGF2BP-dependent manner.

**Figure 6:**
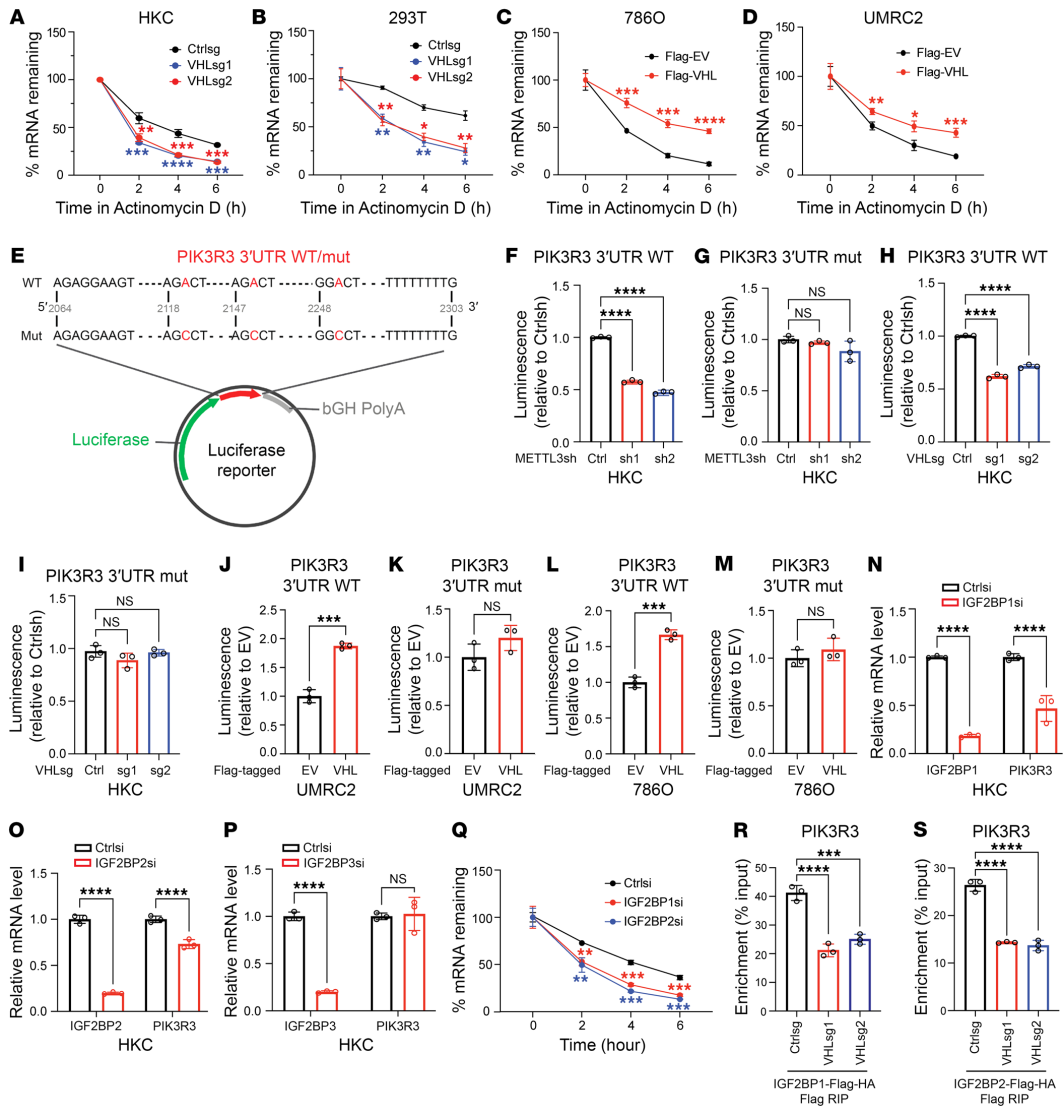
VHL regulates *PIK3R3* mRNA stability in an m6A dependent manner. (**A**–**D**) *PIK3R3* mRNA levels were measured by qPCR in the cells transfected with indicated vectors and treated with Actinomycin D (5 μg/mL) for indicated time. (**E**) Schematic diagram of *PIK3R3* 3′ UTR WT and mutant luciferase reporter. (**F**–**M**) Relative luciferase activity of *PIK3R3* 3′ UTR WT or mutant in HKC cells transfected with indicated vectors. (**N**–**P**) qPCR analysis of *IGF2BP1/2/3* and *PIK3R3* mRNA levels in indicated HKC cell lines transfected with Ctrlsi or *IGF2BP1/2/3*siRNAs. (**Q**) *PIK3R3* mRNA levels were measured by qPCR in HKC cells after treatment with Actinomycin D (normalized to 0 hours). (**R** and **S**), Anti-Flag RIP qPCR test the interaction between *PIK3R3* mRNA and IGF2BP1 (**R**) or IGF2BP2(**S**). Data show mean ± SD, *P < 0.05, **P < 0.01, ***P < 0.001, ****P < 0.0001, 2-way ANOVA (**A**–**D** and **Q**), 1-way ANOVA analysis (**F**–**I**, **N**–**P**, **R**, and **S**) or unpaired *t* test (**J**–**P**).

Since m6A has been reported extensively to regulate mRNA stability, we aimed to determine whether m6A target gene mRNA stability can be regulated by VHL. To this end, we treated cells with the transcription inhibitor actinomycin D followed by pulse chase experiments. We measured RNA stability of target genes identified above. Among them, *PIK3R3* displayed faster RNA decay upon VHL depletion with 2 independent sgRNAs, which was consistent with our RNA-Seq results (Figure 6, A and B). Similar patterns also were observed for other target genes, including *PTK2B*, *PFKFB3*, *ADGRL1*, and *PCSK9* (Supplemental Figure 6A). Conversely, VHL overexpression led to increased *PIK3R3* mRNA stability both in 786O and UMRC2 ccRCC cells (Figure 6, C and D).

To directly prove that methylation modification per se is essential for *PIK3R3* mRNA stability, we have generated a *PIK3R3* WT 3′ UTR reporter construct that harbors all potential m6A binding sites indicated by our m6A RIP-Seq (hereafter referred to as “WT”) (Figure 6E). In addition, we also made the corresponding mutant 3′ UTR reporter that mutated all m6A binding sites (hereafter referred to as “Mut”) (Figure 6E). First, we depleted METTL3 in HKC cells with 2 different shRNAs (Supplemental Figure 6B) and found that, while WT reporter activity was diminished upon METTL3 depletion, Mut reporter activity was not affected (Figure 6, F and G), suggesting that this reporter can recapitulate m6A activity in cells. We also performed VHL depletion by 2 different sgRNAs in VHL-proficient HKC cells and found that, similar to METTL3, VHL depletion led to consistent downregulation of m6A WT reporter activity while not affecting the Mut reporter activity (Figure 6, H and I). Conversely, we overexpressed VHL in 2 representative ccRCC cell lines (UMRC2 and 786O) and found that VHL overexpression led to increased m6A WT reporter activity but not Mut reporter activity (Figure 6, J–M). Our data strongly suggest that VHL regulates *PIK3R3* mRNA stability in a m6A-dependent manner.

The mRNA processing events that occur downstream of m6A deposition are mainly guided by various m6A reader proteins. Therefore, these readers play very important roles in determining the fate of methylated mRNAs (26). As for m6A-mediated mRNA stability, IGF2BP1, 2, and 3 were found to be the major readers that protect mRNA from degradation (27). To confirm which reader protein controls the stability of *PIK3R3* mRNA, we first knocked down each member followed by measurement of *PIK3R3* mRNA levels. We found that siRNA depletion of IGF2BP1 and 2, but not IGF2BP3, led to decreased *PIK3R3* mRNA (Figure 6, N–P), suggesting that IGF2BP1 and 2 are the major m6A readers for regulation of *PIK3R3*. Consistent with this, we also found that IGF2BP1 and 2 depletion led to accelerated *PIK3R3* mRNA degradation upon actinomycin D pulse chase (Figure 6Q). Furthermore, IGF2BP1 and 2 displayed efficient binding in the 3′ UTR region of *PIK3R3* where m6A was localized, and the binding diminished with VHL depletion by 2 independent sgRNAs (Figure 6, R and S and Supplemental Figure 6, C and D). However, VHL depletion did not affect Insulin-like growth factor-2 mRNA-binding proteins (IGF2BPs) protein levels (Supplemental Figure 6E), indicating that the regulation by VHL on *PIK3R3* mRNA is not mediated by affecting levels of IGF2BPs but rather through controlling the m6A modification. Our data suggest that IGF2BP1 and 2 may be the main m6A readers for VHL-regulated m6A signaling.

### VHL regulates PIK3R3 protein levels in ccRCC.

**Figure 7:**
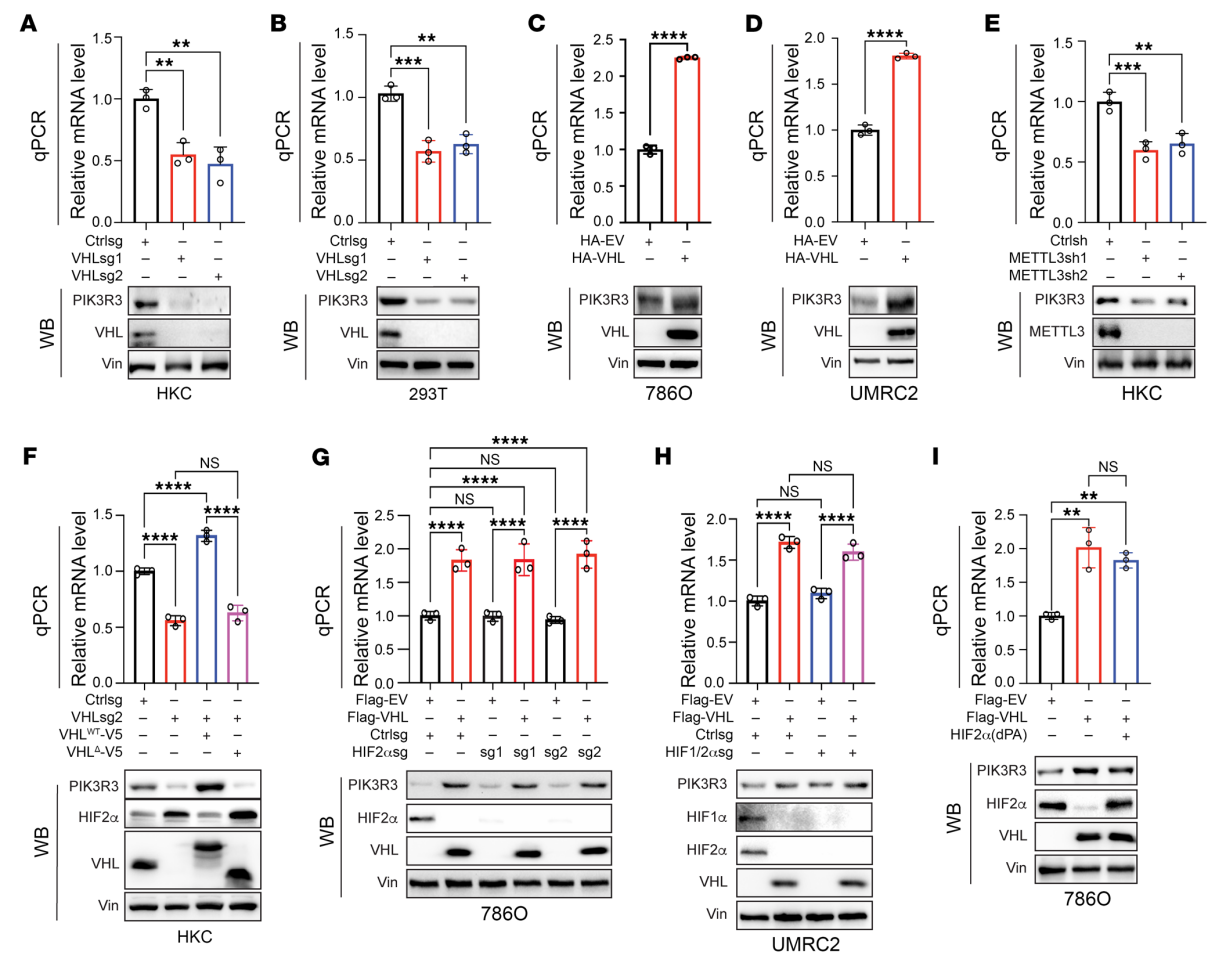
VHL positively regulates *PIK3R3* mRNA and protein levels in ccRCC cells. (**A** and **B**) *PIK3R3* mRNA and protein levels in HKC (**A**) or 293T (**B**) cells transduced with lentivirus either expressing control sgRNA (Ctrlsg) or *VHL*sgRNAs (sg1 and sg2) were detected by qPCR and Western blot, respectively. (**C** and **D**) *PIK3R3* mRNA and protein levels in indicated ccRCC cells that transfected with empty or VHL-overexpressing vectors were detected by qPCR and Western blot, respectively. (**E**) qPCR and immunoblots of samples from HKC cells transduced with lentivirus either expressing control shRNA (Ctrlsh) or *METTL3*shRNAs (sh1 and sh2). (**F**) qPCR and immunoblotting of HKC cells transduced with Ctrlsg or *VHL*sg2 followed by infection with V5-tagged sgRNA resistant WT VHL (VHL^WT^-V5) or VHL E3 ligase deficient mutant (VHL^Δ^-V5). (**G**) qPCR and immunoblotting of 786O cells overexpressed with empty vector or Flag-VHL followed by infection with sgRNA (Ctrlsg) or HIF2-α sgRNAs (sg1 and sg2). (**H**) qPCR and immunoblotting of UMRC2 cells overexpressed with empty vector or Flag-VHL followed by infection with sgRNA (Ctrlsg) or *HIF1/2*α sgRNAs. (**I**) qPCR and immunoblotting of 786O cells overexpressed with empty vector or Flag-VHL followed by infection with HIF2-α (dPA) vector. Statistical analysis was conducted by 1-way ANOVA. Error bars, mean ± SD, **P < 0.01, ***P < 0.001, ****P < 0.0001.

Next, we aimed to further confirm that *PIK3R3* mRNA levels were regulated by VHL. To achieve this, we depleted VHL by sgRNAs in HKC and 293T cells and found decreased *PIK3R3* mRNA in these cells upon VHL depletion, which also corresponded with decreased PIK3R3 protein levels in these cells (Figure 7, A and B). Conversely, we overexpressed VHL in 786O and UMRC2 cells and observed increased *PIK3R3* mRNA and protein levels (Figure 7, C and D). In HKC and RCC4 cells, VHL overexpression led to increased PIK3R3 protein levels (Supplemental Figure 7A). The regulation of *PIK3R3* mRNA and protein levels via the m6A regulatory pathway was also confirmed when we depleted METTL3 by 2 independent shRNAs in HKC cells (Figure 7E). Next, to examine whether the effect of *VHL* sgRNA on PIK3R3 expression was due to its on-target effect, we conducted rescue experiments with a sgRNA-resistant VHL construct and found that VHL overexpression completely rescued the effect on *PIK3R3* mRNA and protein expression. On the other hand, VHL E3 ligase–deficient mutants failed to do so, suggesting that the effect of VHL on *PIK3R3* mRNA and protein levels was dependent on its E3 ligase activity (Figure 7F). To examine whether HIF signaling is important for this regulation, we depleted HIF2-α (EPAS1) by 2 different sgRNAs in 786O ccRCC cells (786O only express HIF2-α, and not HIF1-α as established previously (23)). HIF2-α depletion by sgRNAs did not cause any significant change in *PIK3R3* mRNA or protein levels in 786O cells. In addition, VHL overexpression in HIF2-α depleted cells still led to increased *PIK3R3* mRNA and protein levels, further strengthening that PIK3R3 regulation by VHL is through a HIF-independent manner (Figure 7G). Additionally, we also depleted both HIF1-α and HIF2-α by sgRNAs in UMRC2 cells and found that double depletion of HIFα subunits in these cells did not lead to any difference in *PIK3R3* mRNA or protein levels and VHL overexpression in these cells still led to increased *PIK3R3* mRNA and protein levels (Figure 7H). In 786O ccRCC cells, VHL overexpression led to increased PIK3R3 levels, an effect that was not be rescued by expressing a hydroxylation-deficient HIF2-α (dPA) resistant to VHL recognition (Figure 7I). Conversely, VHL depletion in HKC cells led to decreased PIK3R3, and this effect was not ameliorated by concurrent depletion of ARNT (Supplemental Figure 7B). Cumulatively, our results suggest that VHL positively regulates PIK3R3 in an E3 ligase-dependent but HIF-independent manner.

**Figure 8:**
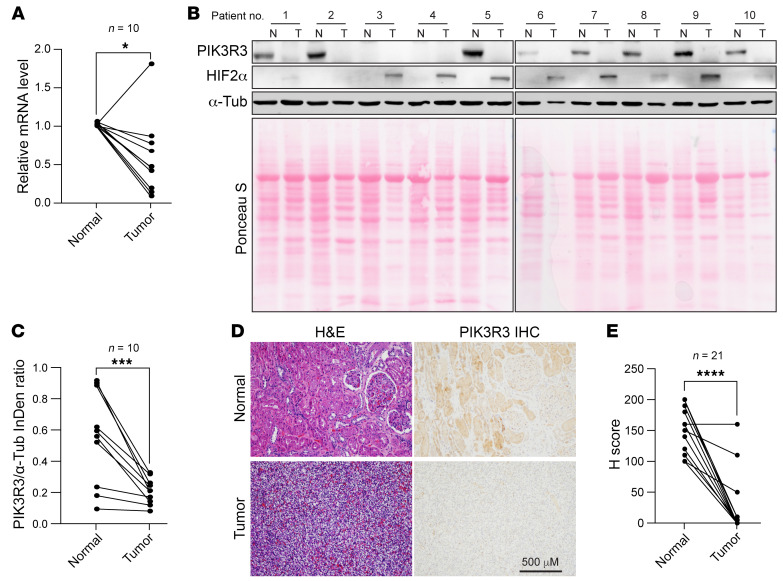
PIK3R3 is downregulated in human ccRCC. (**A**) *PIK3R3* mRNA level in paired patient normal (N) and tumor (T) tissues, *n* = 10. (**B** and **C**) Immunoblotting for lysates from indicated ccRCC paired patient normal (N) and tumor (T) tissues (**B**), and quantification of relative signal intensity of PIK3R3 versus α-Tub (**C**). *n* = 10. (**D** and **E**) Representative images (**D**) and corresponding H-score (**E**) for *PIK3R3* IHC staining in the ccRCC paired patient tissues, *n* = 21. Statistical analysis was conducted by paired 2-tailed *t* test. Error bars, mean ± SD, *P < 0.05, ***P < 0.001, ****P < 0.0001.

In order to examine the correlation between VHL and PIK3R3, we collected cell lysates from a panel of renal epithelial cell lines (such as 293T and HKC), VHL-positive RCC cell lines (such as Caki-1 and ACHN) and VHL-null RCC lines (including UMRC2, UMRC6, 786O, A498, RCC4, and Caki-2). Consistently, we found a strong correlation between PIK3R3 and VHL protein levels (*r* = 0.8744), suggesting that VHL-PIK3R3 regulation is relevant in ccRCC cell lines (Supplemental Figure 7, C and D). In addition, qPCR on mRNA samples from 10 frozen ccRCC tumor and normal sample pairs revealed that the mRNA level of *PIK3R3* is decreased in ccRCC tumors compared with normal samples (Figure 8A). Correspondingly, we also examined HIF2-α (as a surrogate marker for VHL depletion) and PIK3R3 in human tissue samples. Indeed, HIF2-α accumulation in tumors corresponded with diminished PIK3R3 protein levels in these samples, suggesting that PIK3R3 was lost during the course of ccRCC development (Figure 8, B and C). Additionally, we assayed PIK3R3 expression in 21 pairs of FFPE ccRCC tumor and normal samples by IHC. The validity of the PIK3R3 antibody for IHC was confirmed using HKC cells with PIK3R3 overexpression or knockout, where PIK3R3 overexpression led to increased staining intensity while PIK3R3 knockout resulted in decreased PIK3R3 staining (Supplemental Figure 8A). By performing IHC with FFPE samples, we observed decreased PIK3R3 protein levels in ccRCC tumors compared with normal samples (Figure 8, D and E). However, through RNA-Seq analysis with human patient tumors and nonmalignant samples from IMmotion151 clinical trial subtypes, we didn’t find a significant change in the *PIK3R3* RNA level (Supplemental Figure 8B). This discrepancy could be attributed to the heterogeneity of solid tumors and potential interference from stromal cells in the tumor microenvironment. To exclude the effects of stromal cells we examined the PIK3R3 expression with RNA-Seq in our PDX platform (28, 29) because human stromal cells originally present in tumors will gradually be replaced by host stromal cells as the xenograft grows (30). Notably, we observed significantly lower PIK3R3 expression levels in ccRCC tumorgrafts compared with nonmalignant counterparts (Supplemental Figure 8C). In addition, survival analysis with the data from IMmotion151 clinical subtypes and TCGA data set suggested that decreased *PIK3R3* mRNA level corresponded with better survival probability in patients with ccRCC (Supplemental Figure 8, D and E). In summary, our results suggest that VHL positively regulates PIK3R3 in ccRCC.

### PIK3R3 suppresses tumor growth in vitro and in vivo.

**Figure 9:**
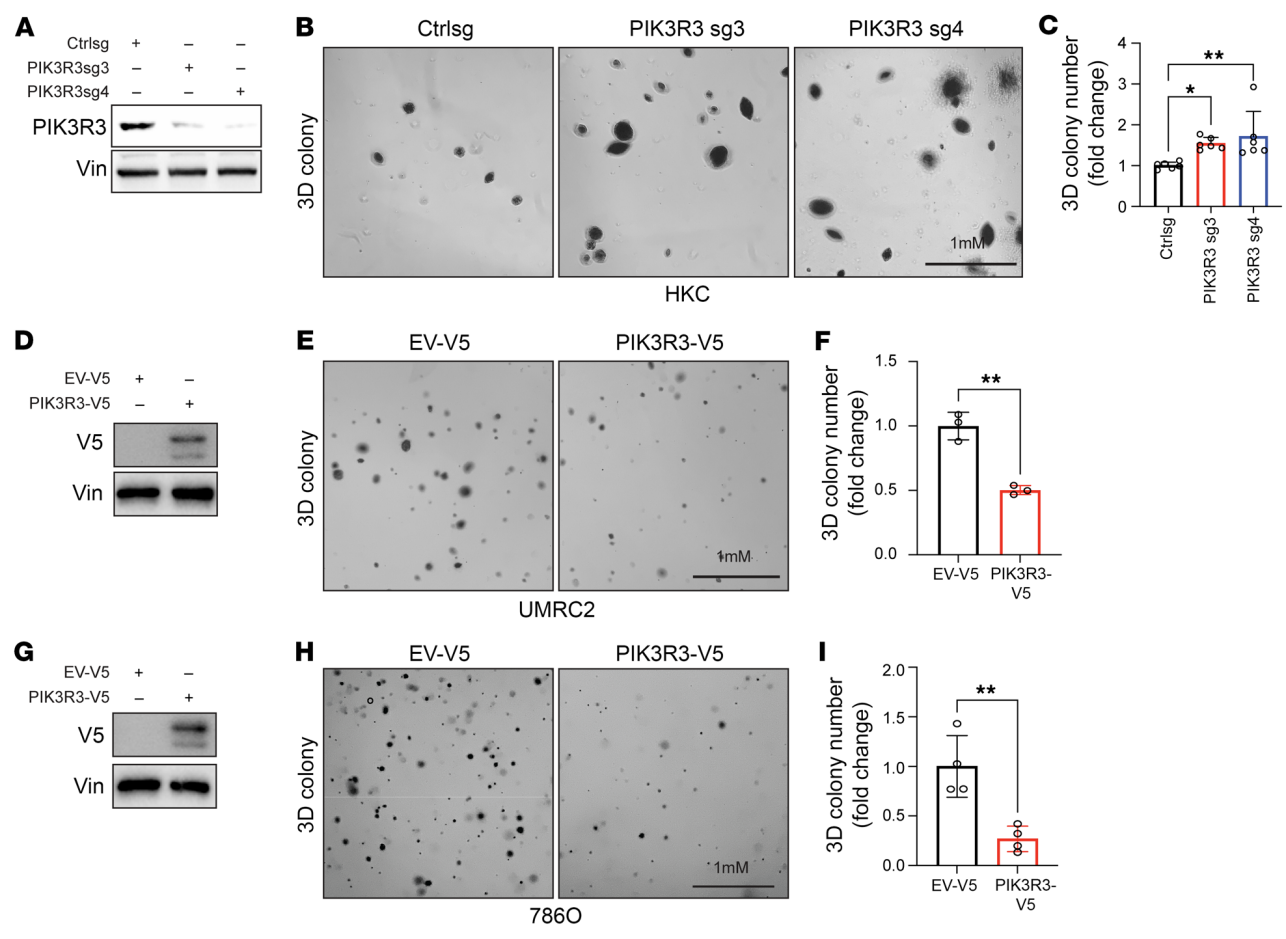
PIK3R3 suppresses tumor growth in vitro. (**A**–**C**) Immunoblotting (**A**), 3D colony formation assay (**B**), and corresponding quantification data (*n* = 6) (**C**) in HKC cell lines transduced with indicated sgRNAs. (**D**–**I**) Immunoblotting (**D** and **G**), 3D colony formation assay (**E** and **H**), and corresponding quantification data (*n* = 3) (**F** and **I**) in UMRC2 or 786O cell lines transduced with indicated overexpressing vectors. V5 and Vin blots were run in parallel using the same biological samples. Data show mean ± SD, *P < 0.05, **P < 0.01, 1-way ANOVA analysis (**C**) or unpaired *t* test (**F**–**I**).

To examine the effect of PIK3R3 in ccRCC, first we depleted PIK3R3 in HKC cells followed by assay of 2-D colony formation and 3-D soft agar growth assay. PIK3R3 depletion led to increased colony formation both in 2-D and 3-D (Figure 9, A–C and Supplemental Figure 9, A and B). Conversely, we also overexpressed PIK3R3 in UMRC2 and 768O cells and observed decreased cell growth in both 2-D and 3-D in both cell lines (Figure 9, D–I and Supplemental Figure 9, C–F).

**Figure 10:**
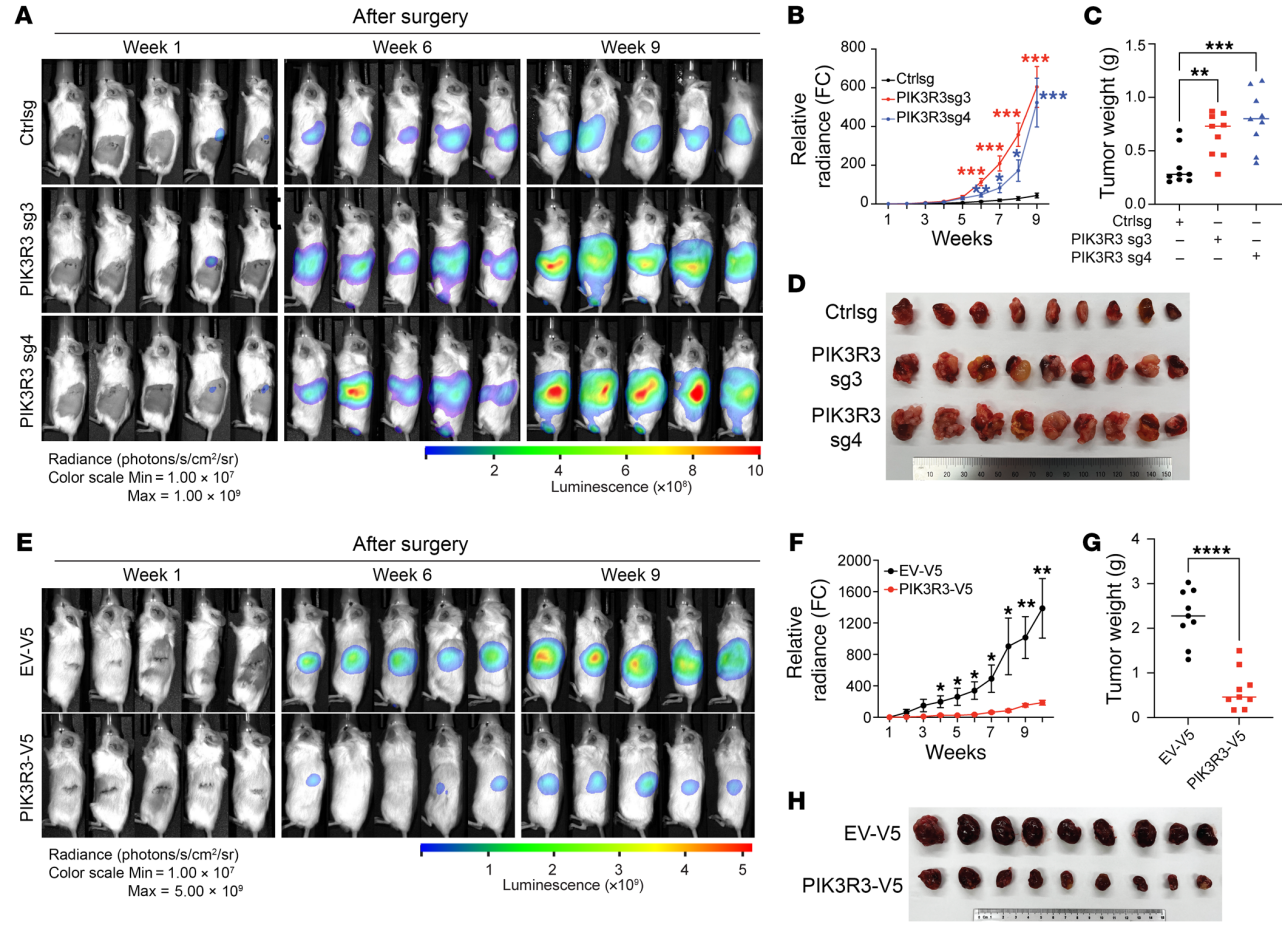
PIK3R3 suppresses tumor growth in vivo. (**A**–**D**) Representative bioluminescence imaging (**A**) of mice renal subcapsule orthotopic injected with Ctrlsg or *PIK3R3*sg transduced HKC luciferase cell lines and corresponding quantitation data of bioluminescence signal (**B**), tumor weight (**C**), or tumors images (**D**) after dissection. (**E**–**H**) Representative bioluminescence imaging (**E**) of mice renal subcapsule orthotopic injected with empty vector or PIK3R3-V5 transduced UMRC2 luciferase cell lines and corresponding quantitation data of bioluminescence signal (**F**), tumor weight (**G**), tumors images (**H**) after dissection. Data show SEM for **B** and **F** or mean ± SD for others; *P < 0.05, **P < 0.01, ***P < 0.001, ****P < 0.0001, 2-way ANOVA (**B** and **F**), 1-way ANOVA (**C**) or unpaired *t* test (**G**).

To examine the effect of PIK3R3 on tumor development, we orthotopically injected HKC cells with either Ctrl sgRNA or 2 *PIK3R3* sgRNAs (sg3 and sg4) into the kidney capsules and monitored tumor growth over time with weekly bioluminescence imaging. PIK3R3 depletion by 2 independent sgRNAs all led to increased tumor progression over time, which also corresponded with increased tumor weights and bigger tumor size at necropsy (Figure 10, A–D). Additionally, we also examined potential lung metastasis ex vivo and found that PIK3R3 depletion led to increased lung metastasis (Supplemental Figure 10, A and B). Conversely, we also overexpressed PIK3R3 in UMRC2 cells and observed decreased tumor progression and size as well as lung metastasis (Figure 10, E–H and Supplemental Figure 10, C and D). In support of our conclusion, we also injected UMRC2 cells engineered with PIK3R3 overexpression subcutaneously into the flanks of NSG mice and observed decreased tumor progression and tumor size (Supplemental Figure 10, E–G). Overall, PIK3R3 depletion led to increased tumorigenesis while its overexpression resulted in decreased ccRCC tumor progression. Therefore, PIK3R3 may serve as a tumor inhibitory factor in ccRCC.

### PIK3R3 suppresses ccRCC by antagonizing PI3K activation.

**Figure 11:**
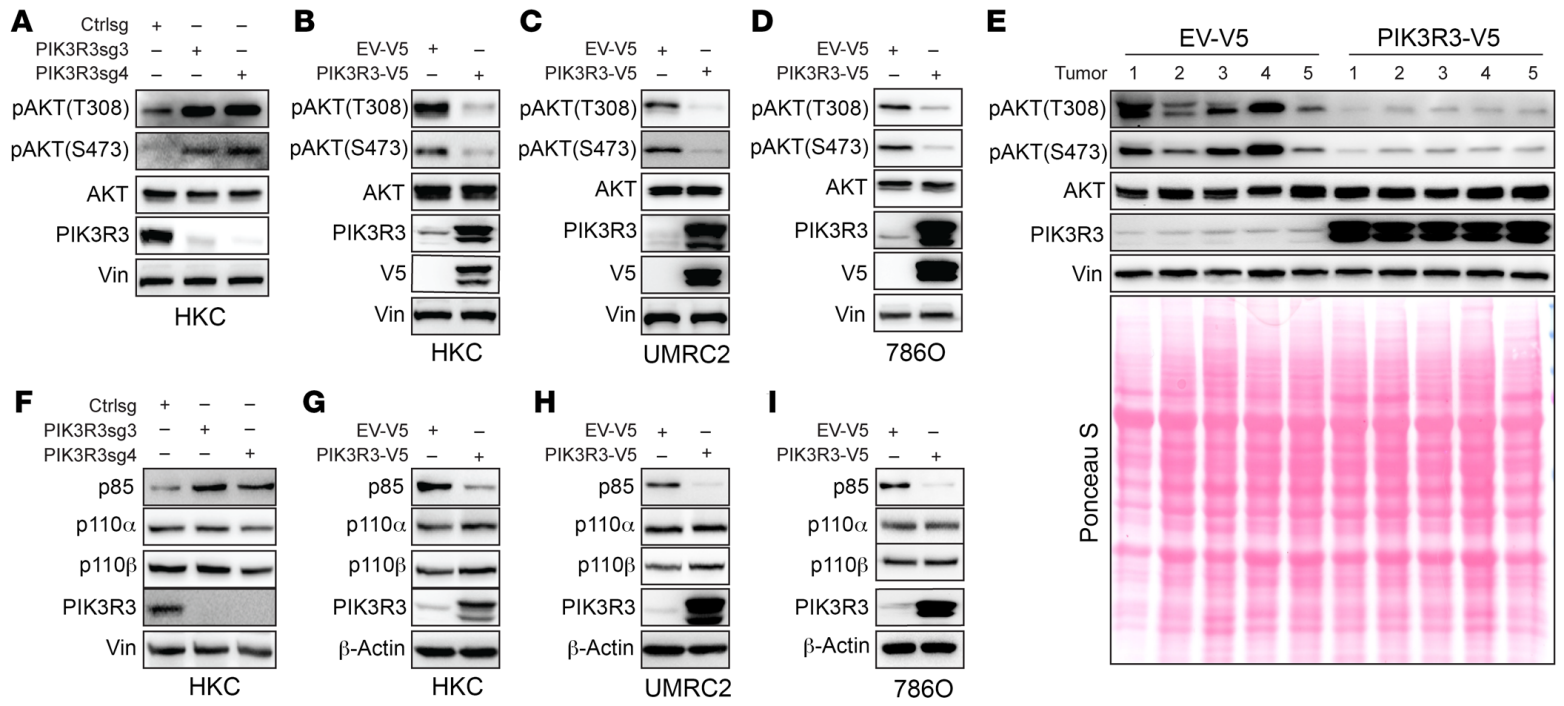
PIK3R3 suppresses AKT signaling. (**A**–**D**) Immunoblotting analysis shows increased AKT phosphorylation in PIK3R3-KO HKC cells (**A**) or decreased AKT phosphorylation in PIK3R3 overexpressing cell lines (**B**–**D**). pAKT(T308), pAKT(S473), AKT, and PIK3R3 blots were run in parallel using the same biological samples. (**E**) Immunoblotting with lysate of tumors grown out from mice renal subcapsule orthotopic injected empty vector or PIK3R3-V5 transduced UMRC2 luciferase cell lines. pAKT(T308), pAKT(S473), AKT, and PIK3R3 blots were run in parallel using the same biological samples. (**F**–**I**) Immunoblotting analysis shows increased p85 in PIK3R3-KO HKC cells (**F**) or decreased p85 in PIK3R3 overexpressing cell lines (**G**–**I**). P110-α and P110-β blots were run in parallel using the same biological samples.

The molecular mechanism underlying how PIK3R3 regulates renal tumorigenesis remains unclear. PI3K3R3 is a subunit of PI3K signaling according to the previous literature (19, 31). On the other hand, it is unclear on how PIK3R3 may regulate PI3K signaling in renal cancer. To this end, we first depleted PIK3R3 in HKC cells with 2 independent sgRNAs and found that PIK3R3 depletion led to increased pAKT both on serine 473 and threonine 308 residues (Figure 11A), an important indicator of activated PI3K signaling. Conversely, overexpression of PIK3R3 in multiple cell lines (including HKC, 786O,and UMRC2) led to decreased pAKT signaling (Figure 11, B–D). We also performed Western blots with cell lysates harvested from UMRC2 orthotopic xenograft tumors expressing either control or PIK3R3. Consistently, PIK3R3 overexpression in UMRC2 tumors led to decreased pAKT levels (Figure 11E). Taken together, these data suggest that PIK3R3 is a negative regulator for PI3 kinase activation.

Mechanistically, we examined the effect of PIK3R3 on key signaling molecules in PI3K signaling, including p85, p110-α, and p110-β. Interestingly, PIK3R3 depletion led to increased p85 levels while its overexpression led to decreased p85 subunit in kidney cells (Figure 11, F–I). However, the mRNA level of p85 did not exhibit a significant change upon the knockout or overexpression of PIK3R3 (Supplemental Figure 11, A and B). This regulation appeared to be specific as protein levels of p110-α and p110-β were not affected by either PIK3R3 depletion or overexpression (Figure 11, F–I).

**Figure 12:**
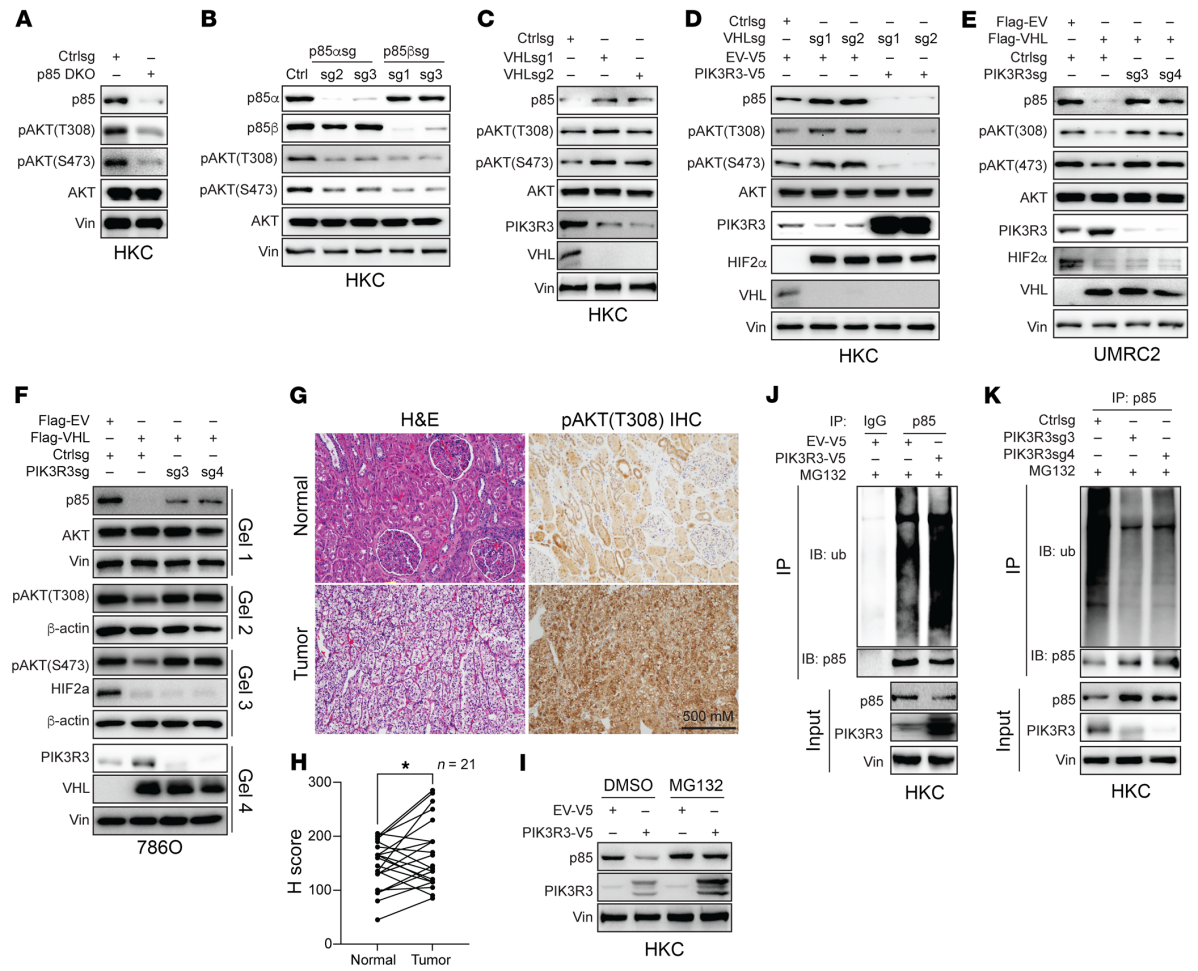
VHL suppresses AKT signaling by upregulating PIK3R3. (**A**) Immunoblot of cell lysate from HKC cells transduced with Ctrlsg or *p85* DKO sgRNAs. (**B**) Immunoblotting of HKC cells infected with Ctrlsg, *p85*α sgRNAs (2 and 3), or *p85*β sgRNAs (1 and 2). (**C**) Immunoblotting of HKC cells infected with Ctrlsg or VHLsg (1 and 2). (**D**) Immunoblotting of HKC cells transduced with Ctrlsg or *VHL*sg (sg1 and sg2) followed by infection with empty vector or PIK3R3-V5. (**E** and **F**) Immunoblotting of UMRC2 (**E**) or 786O (**F**) cells infected with empty vector or Flag-VHL followed by transduction of Ctrlsg or *PIK3R3*sgRNAs (sg3 and sg4). P85, pAKT(T308), pAKT(S473), AKT, and PIK3R3 blots were run in parallel using the same biological samples. (**G**–**H**) Representative images (**G**) and corresponding H-score (**H**) for pAKT (T308) IHC staining in the ccRCC paired patient tissues, *n* = 21. Statistical analysis was conducted paired 2-tailed *t* test. Error bars, mean ± SD *P < 0.05. (**I**) Immunoblots of lysates from HKC cells transfected with empty vector or PIK3R3-V5 and then treated with DMSO or MG132 as indicated. (**J** and **K**) Anti-p85 immunoprecipitation with the samples from HKC cells transfected with overexpressing vector (**J**) or sgRNAs (**K**) and then treated with MG132.

The p85 contains 2 subunits, including p85-α and p85-β. To examine the effect of p85 on PI3K activation, we used a mixed sgRNA pool targeting both p85-α and p85-β and found that p85 depletion by this pool sgRNA infection led to decreased AKT phosphorylation on both the 308 and 473 sites (Figure 12A). To dissect which isoform of p85 may be important for its regulation on AKT phosphorylation, we implemented 2 independent sgRNAs against each isoform and found that these sgRNAs both depleted target protein expression. Interestingly, depletion of either isoform led to decreased pAKT (Figure 12B), suggesting that both isoforms may be important with regard to regulating AKT phosphorylation. To simplify our study thereafter, we used a pan-p85 antibody to detect p85 protein levels in these cells. VHL depletion led to decreased PIK3R3 protein levels, which corresponded with increased p85 protein levels and increased pAKT levels (Figure 12C). To examine whether decreased PIK3R3 upon VHL loss accounted for increased p85 protein levels and increased pAKT, we overexpressed PIK3R3 in cells with VHL depletion (by sgRNAs) and found that the effect of AKT phosphorylation induction by VHL loss was ameliorated (Figure 12D). Conversely, we depleted PIK3R3 in UMRC2 and 786O cells that overexpressed VHL. Whereas VHL overexpression led to increased p85 and decreased pAKT on 308 and 473 sites, this effect can be ameliorated by concurrent depletion of PIK3R3 (Figure 12, E and F). In addition, we observed decreased pAKT (308) levels in ccRCC tumors compared with normal samples by IHC in FFPE samples (Figure 12, G and H).

To determine how PIK3R3 regulates p85 protein levels in ccRCC, we treated PIK3R3-overexpressing HKC cells with vehicle control or proteasomal inhibitor MG132, as previous studies have shown p85 can be degraded through ubiquitination proteosome pathway (32). PIK3R3 overexpression led to decreased p85 protein level, the effect abrogated upon MG132 (Figure 12I). This suggests that PIK3R3 regulates p85 protein stability. In accordance with this observation, we also observed increased p85 poly ubiquitination upon PIK3R3 overexpression (Figure 12J). Conversely, PIK3R3 depletion led to decreased p85 poly ubiquitination and increased p85 protein levels (Figure 12K).

ccRCC tumors are characterized by increased AKT phosphorylation and PI3 kinase activation, which was reported previously (33). Indeed, we also found that pAKT was upregulated in patients with ccRCC compared with paired normal controls. To functionally explore the role of AKT phosphorylation on mediating the effect of PI3KR3 depletion on ccRCC growth, we treated Ctrl and PIK3R3 sgRNA-infected cells with the AKT inhibitor (Capivasertib), which led to increased AKT phosphorylation but caused a decrease in AKT kinase activity (34). Cell viability was measured at varying concentrations of Capivasertib to determine the appropriate dosage. We found 2.5 μM of Capivasertib did not suppresses the cell viability (Supplemental Figure 11C) in different cells (including HKC, UMRC2, and 786O) but resulted in reduced AKT activity, as evidenced by the decreased phosphorylation of its subsequent targets, including pGSK3-β (S9) and pPRAS40 (T246) (Supplemental Figure 11, D–F). Consistently, PIK3R3 depletion led to increased 2-D and 3-D colony formation, and this effect was abrogated by concurrent treatment with these inhibitors (Supplemental Figure 12A). Taken together, we argue that VHL positively regulates PIK3R3 by stabilizing its mRNA in an m6A-dependent manner and thus results in increased PIK3R3. PIK3R3 stabilization causes increased p85 ubiquitination and decreased p85 protein levels, which leads to decreased AKT phosphorylation in ccRCC.

## Discussion

This study highlights the VHL protein’s role in controlling m6A modification and RNA stability in ccRCC cells. We found that VHL interacts with various m6A complex proteins like METTL13, METTL3, METTL14, and WTAP. Depletion of VHL reduces m6A levels, while its overexpression increases them in kidney cells. Mechanistically, VHL regulates the interaction between METTL3 and METTL14, affecting m6A levels. By performing m6A RIP-Seq, we identified numerous m6A sites that are differentially regulated upon VHL depletion. Additionally, we have performed integrated analysis for m6A RIP-Seq and RNA-Seq and identified genes that show differential m6A and differential expression upon VHL depletion. Among them, we have focused on 13 genes, including *PIK3R3*, which was enriched in several pathways. *PIK3R3* mRNA stability is regulated by VHL in an m6A and IGF2BP-dependent manner; VHL depletion resulted in faster RNA decay of *PIK3R3* and other target genes, suggesting that VHL plays a role in regulating mRNA stability. The stability of *PIK3R3* mRNA was found to be dependent on m6A modification and interaction with IGF2BPs, specifically IGF2BP1 and 2. PIK3R3 is a negative regulator for PI3K/AKT activation in ccRCC and decreased PIK3R3 upon VHL loss leads to increased PI3K activation and increased tumorigenesis. Conversely, VHL restoration led to increased PI3KR3 and decreased PI3K and AKT activation, therefore contributing to decreased ccRCC tumorigenesis. Overall, our findings underscore VHL’s crucial role in ccRCC by regulating m6A modification and RNA stability, potentially impacting renal cell carcinoma development and progression.

The significance of VHL regulation on m6A and mRNA stability regulation lies in its role as a critical tumor suppressor in ccRCC and its ability to modulate key cellular processes involved in gene expression control. VHL, known as a canonical E3 ligase that regulates protein stability (35), may also play a role beyond its function as an E3 ligase (35). In this study, we show that VHL regulates METTL3/METTL14 protein complex formation. However, VHL does not robustly regulate METTL3/14 enzymatic activity in a pure in vitro system, suggesting that this regulation may primarily occur in vivo. We also provide evidence that the regulation of VHL on m6A activity is dependent on its E3 ligase domain but independent of its canonical substrates such as HIF1-α or HIF2-α. Knocking out or overexpressing VHL did not impact the protein levels of METTL3 and METTL14, indicating that VHL’s role in this context does not depend on its E3 ubiquitin ligase function governing protein stability. It appears that VHL interacts with METTL3/14 via its E3 ligase domain (where substrates normally bind) and acts as an adaptor that facilitates the formation of the METTL3/METTL14 complex. The VHL-regulated METTL3/METTL14 complex, in turn, modifies and regulates the stability of RNAs (e.g., *PIK3R3*) through m6A modification. However, it is currently unclear how VHL regulates METTL3/14 complex formation. RNA m6A modification is known to be a dynamic modification, as it can be deposited by the m6A writer complex and removed by eraser proteins (36). In addition to the writer complex proteins, the m6A erase proteins have also been identified as key players in renal cell carcinoma. For example, m6A RNA demethylase FTO was identified as a synthetic lethal partner of VHL in ccRCC (13) and was indispensable to the growth of HIF2^lo/−^ ccRCC(12). Whether VHL regulates FTO protein’s function and its underlying mechanism remain to be investigated. Our study further demonstrates that VHL depletion results in altered mRNA stability of target genes, including *PIK3R3*. *PIK3R3* mRNA displayed accelerated decay upon VHL depletion, suggesting that VHL plays a role in stabilizing target mRNAs. From our integrated m6A RIP-Seq and RNA-Seq data analysis, it appears that VHL predominantly positively regulated mRNA stability in a m6A-dependent manner. These findings are intriguing as it is unclear how these specific mRNA regulations take place in a VHL-dependent manner, which can be potentially explained by the specific m6A reader proteins, such as IG2FBPs, as discussed below. In addition, whereas our current study focuses on the characterization of *PIK3R3* as an m6A target in a VHL-dependent manner in ccRCC, the functional role of other target genes (such as *PCSK9*, *SMAD6*, *SCL45A4*, and others) in ccRCC remain to be determined and characterized. This regulation of mRNA stability by VHL could have broad implications for gene expression and cellular processes, potentially influencing cell proliferation, survival, and other cancer-related pathways.

IGF2BPs including IGF2BP1, IGF2BP2, and IGF2BP3 function as m6A reader proteins to promote the stability of their target mRNAs (37). Abundant evidence reveals that IGF2BPs are involved in multiple physiological and pathological processes, including in human cancers (such as TNBC (38), CRC (39), NSCLC (40), and ccRCC (14), as well as others). In this study, we found that IGF2BP1 and IGF2BP2 recognize *PIK3R3* m6A and promote its mRNA stability downstream of VHL. YTH domain proteins are another family of main m6A readers that regulate RNA stability (41). We examined the effects of YTHDF2 and YTHDC2, which are reported to regulate m6A-mediated RNA degradation (42) and found that their knockdown led to slightly increased *PIK3R3* (Supplemental Figure 6F). In our studies, we found that VHL KO suppressed m6A modification and promoted mRNA degradation. Thus, we mainly focused on the readers that are mainly responsible for promoting RNA stability, such as the IGF2BP proteins.

PIK3R3, also known as phosphoinositide3-kinase regulatory subunit 3, is a regulatory subunit of the phosphoinositide 3-kinase (PI3K) enzyme (43). Dysregulation of PI3K/AKT pathway is commonly observed in cancer, including renal cancer (44). In this study, we identified the tumor suppressive role of PIK3R3 in renal cancer by negatively regulating PI3K/AKT activation. In the present study, we found that PIK3R3 was downregulated in ccRCC tumors compared with normal tissues. Our results on the VHL regulation of *PIK3R3* mRNA stability support our observation of decreased PIK3R3 in patients with ccRCC, which is further confirmed by our PIK3R3 IHC staining results in ccRCC tumor and normal tissues. Mechanistically, we provide evidence that PIK3R3 overexpression promoted p85 subunit ubiquitination and degradation while PIK3R3 downregulation led to increased p85 protein stability by decreasing its ubiquitination and degradation. Our result demonstrates the critical role of PIK3R3 via negatively regulation of PI3K/AKT activation in ccRCC. The detailed mechanism of how PIK3R3 regulates p85 protein stability remains to be studied in ccRCC. It is important to note that our results show depletion of either isoform of p85 led to decreased pAKT, suggesting that either p85-α or p85-β positively regulates AKT phosphorylation (Figure 12B). The positive role of p85-α on AKT activation in our findings is in contrast with previous reports that p85-α restrains catalytic activity of PI3K and serves as a tumor suppressor in multiple cancer types such as breast cancer, lung cancer, and colorectal cancer (45), suggesting that the effect of the p85 subunit on AKT activation may depend on specific cellular context. Although the protein level of p85-α is lower in many cancer types, it is significantly higher in KIRC, which provides another piece of evidence that p85-α may play a different role in kidney cancer (46). However, the detailed mechanism by which p85-α regulates PI3K in kidney cancer remains to be investigated.

This study sheds light on how VHL affects m6A modification and RNA stability in ccRCC. The findings highlight the importance of VHL in regulating key components of the m6A enzymatic complex and its ability to modulate m6A levels. The identification of downstream target genes regulated by VHL through m6A modification expands our understanding of the functional consequences of VHL loss in ccRCC. Additionally, the study suggests a potential therapeutic strategy targeting the VHL/m6A/IGF2BP axis to modulate mRNA stability in ccRCC. Further research is warranted to explore the precise mechanisms by which VHL influences m6A modification and RNA stability and to uncover additional downstream target genes and pathways. Additionally, investigating the clinical relevance of VHL-mediated m6A regulation in patient samples and evaluating the therapeutic potential of targeting m6A writers, readers, or erasers in ccRCC could be avenues for future studies. Overall, this study sets the stage for further exploration of the VHL-m6A axis and its implications in renal cancer biology.

## Methods

### Sex as a biological variable.

In orthotopic xenograft tumor growth studies, male to female mouse ratio is set at 2:1 to mirror the human scenario where men are twice as likely as women to develop kidney cancer and also exhibit a higher mortality rate (47).

### Tumor xenograft and mouse image.

Xenograft tumor growth was performed with 6–8 week-old NSG mice (Jackson Lab) by injecting luciferase reporter cell lines into mice orthotopically. Approximately 5 × 10^5^ HKC or 1 × 10^6^ UMRC2 cells were resuspended in 25 μL Matrigel and diluted with medium by 1:1 and then injected into the left kidney of each mouse as described previously (48). To monitor the tumor growth in vivo, mice were i.p. injected at 150 mg/kg with the 15 mg/mL stock of D-Luciferin (Goldbio, LUCK-1G) and the signal was monitored weekly under a spectral AMI-HTX imaging system. Mice were euthanized 9–10 weeks after the cell injection. The kidneys of each mouse were taken out and weighed. Tumor weight was calculated by subtracting the weight of left kidney containing the tumor from weight of the right normal kidney. For the lung ex vivo imaging, lungs were taken out and immersed in 300 mg/mL D-Luciferin in 24-well plates followed by AMI-HTX imaging immediately.

### Cell culture.

HKC cells are obtained from Kimryn Rathmell from Vanderbilt University. Short tandem repeat (STR) assay was performed to validate the identity of HKC cells. 786O, 293T cells were purchased from ATCC while the UMRC2 cell line was from Sigma-Aldrich. All the cells were maintained in DMEM (Gibco, 11995073) supplemented with 10% FBS and 1% penicillin/streptomycin. The cells were incubated at 37°C in a humidified atmosphere containing 5% CO_2_. Mycoplasma testing was routinely carried out with MycoAlert PLUS Mycoplasma Detection Kit (Lonza, LT07-703) to ensure cells were mycoplasma free.

### shRNA, sgRNA, and siRNAs.

*METTL3* and *IGF2BP1* lentiviral shRNA constructs were purchased from Sigma-Aldrich. VHL, PIK3R3, *HIF1-α*, *HIF2-α*, *ARNT*, *IGF2BP2*, *IGF2BP3*, *p85*α, and *p85*β sgRNAs were cloned into pLentiCRISPR V2 -Puromycin vectors. siRNAs were purchased from Thermo Fisher Scientific. Target sequences or catalog numbers of sgRNAs, shRNAs, and siRNAs are listed in Supplemental Table 1.

### Transfection, Virus packaging, and infection.

293T or HKC cells were transfected with indicated plasmids following the instruction of Lipofectamine 3000 Transfection Reagent (Thermo Fisher Scientific, L3000150). Cell lysate was collected and subjected to Western blot or co-IP assay after 48 hours of transfection. *IGF2BP* siRNAs were transfected into HKC cells with Lipofectamine RNAi MAX Transfection Reagent (Thermo Fisher Scientific, 13778500). After 48 hours of transfection, the cells were subjected to RNA stability assay or cell lysate and RNA collection. Lentivirus transfer plasmids were cotransfected with pMD2.G and psPAX2 helper plasmids to 293 T cells using Lipofectamine 3000 Transfection Reagent. Medium was changed after overnight transfection, and the supernatant containing the virus was collected twice, at 48 hours and 72 hours after transfection by passing through a 0.45 μm filter. 200 μL virus was applied to the cells that seeded in 35 mm dishes at 30%–50% confluence in presence of 8 μg/mL polybrene. The medium was changed 6 hours after infection, and the cells were subjected to the appropriate antibiotic (puromycin, 2 μg/mL; Blasticidin 10 μg/mL; Hygromycin, 100 μg/mL; or Zeocin, 100 μg/mL) selection 48 hours after infection.

### m6A dots blot.

The Poly(A) + mRNAs were purified with the Dynabeads mRNA Purification Kit (Thermo Fisher Scientific, 61006) following the manufacturer’s instructions. mRNA samples were quantified using NanoDrop and diluted to 100 ng/μL and 50 ng/μL with RNase-free water. The mRNAs were first denatured at 65°C for 5 minutes. Application of 3 μL of denatured mRNA to Amersham Hybond-N + membrane (GE Healthcare) was followed by UV crosslinking. Methylene blue was used to detect the mRNA amount on the membrane. The membrane was then washed with 10% SDS buffer, blocked with 5% nonfat milk in PBST (PBS with 0.02% Tween-20), and incubated with anti-m6A antibody (Synaptic Systems, 202003, 1:1,000) overnight at 4°C. HRP-conjugated Goat anti-rabbit IgG (Thermo Fisher Scientific, 31460) was added to the blots and then developed with ultra-sensitive enhanced chemiluminescent (Thermo Fisher Scientific, 34094).

### Co-IP and pull-down assay.

The Co-IP assay and pull-down assay were performed as described previously (49). For Co-IP assay, cell lysates were prepared with EBC buffer (0.1 mM EDTA, 50 mM Tris-HCl pH 8.0, 120 mM NaCl, 0.5% NP-40, and 10% Glycerol) supplemented with protease inhibitor cocktail (Roche) and phosphoSTOP tablets (Roche). Approximately 1 μg protein from the cell lysate was incubated with appropriate antibias or Anti-HA Affinity Matrix (Roche, 11815016001) or anti-Flag M2 affinity gel (Sigma-Aldrich, A2220) for overnight at 4°C. For the samples that incubated with antibodies was added 10 μL of prewashed Protein G beads (Roche, 11243233001) and further incubation at 4°C for another 2 hours. The beads were washed 5 times with EBC buffer and the immunoprecipitated proteins was recovered by 1 × protein loading buffer (LB) and subjected to Western blot assay. For the pull-down assay, the proteins that expressed by IVT system or purified from *E*. *coli* bacterial (BL21) were coincubated with corresponded beads as indicated in EBC buffer overnight at 4°C. To exclude the inferring of RNA on the interaction, 5 μL of RNase Cocktail (Invitrogen, AM2288) were added during the incubation. After washing 5 times with EBC buffer, the beads with bound proteins were boiled with 1 × LB buffer and subjected to SDS-PAGE.

### Western blotting.

Western blotting was performed as previously described (50). In brief, whole cell or tissues sample lysates were prepared with EBC buffer (0.1 mM EDTA, 50 mM Tris-HCl pH 8.0, 120 mM NaCl, 0.5% NP-40, 10% Glycerol) supplemented with protease inhibitor and phosphoSTOP (Roche). Tissue samples were homogenized with TissueRuptor II (QIAGEN) in EBC buffer. After centrifuge at 20,000*g*, 4°C for 10 minutes, protein concentration of the supernatant was measured with Bio-Rad protein assay dye reagent. Equal amount of protein samples were loaded and resolved by SDS-PAGE. The protein samples were transferred to Nitrocellulose membranes and blocked with 5% nonfat milk. After incubation with primary antibodies overnight, the NC membranes were washed and subsequently incubated for 1 hour with secondary antibodies. All the antibody information is listed in Supplemental Table 3. Chemiluminescent substrates ECL or SuperSignal West Femto maximum sensitivity substrate were used for singling development on a ChemiDoc Imaging System (Bio-Rad).

### In vitro methylation assay.

The in vitro methylation assay was conducted in triplicate as previously described (51). In brief, the 15 μL mixture consisted of 200 nM RNA substrate, 20 mM Tris (pH 7.5), 0.01% Triton-X (Sigma-Aldrich), 1 mM DTT, 50 μM ZnCl_2_ (Sigma-Aldrich), 0.2 U/μL RNasin (Thermo Fisher Scientific), 1% glycerol (Sigma-Aldrich), and 460nM [3H]-SAM (Perkin Elmer). The recombinant METTL3/METTL14 complexes were purified from *E*. *coli* and recombinant VHL was purified from SF9 insect cells. Each reaction was incubated at 37°C for 2 hours. The levels of RNA with the incorporated 3H-methyl group are presented as disintegrations per minute (DPM). The mean ± SD of the in vitro methylation data is presented based on 3 replicates.

### MeRIP and Seq data analysis.

mRNAs were extracted with Dynabeads mRNA Purification Kit (Invitrogen, 61006) following the manufacturer’s manual instruction. The mRNAs were treated with DNase I (Sigma-Aldrich, 04716728001) to eliminate genome DNA contamination and then were fragmented with RNA Fragmentation Reagents (Invitrogen, AM8740). After precipitation, the RNA pellets were resuspended in Nuclease free water and concentration was measured with qubit. The pos-fragmentation size (approximately 100 nt) of mRNA was confirmed by running the mRNAs on a tape station. 6 μg of fragmented mRNAs were incubated with m6A antibody (Synaptic Systems, 202003, 1:100) in 200 μL reaction buffer (150 mM NaCl, 10 mM Tris-HCl pH 7.7 and 0.1% NP-40, supplemented with RNase inhibitor before using) for 2 hours at 4°C, followed by the addition of prewashed Protein A/G beads (Thermo Fisher Scientific, PI88802) and further incubation at 4°C for another 1 hour. The beads were serially washed 2 times with reaction buffer, 2 times with low salt reaction buffer (50 mM NaCl, 10 mM Tris-HCl pH 7.7 and 0.1% NP-40) and 2 times with high salt reaction buffer (500 mM NaCl, 10 mM Tris-HCl pH 7.7 and 0.1% NP-40). Immunoprecipitated RNA was extracted with RNeasy mini kit (Qiagen, 74106). A cDNA library was prepared with NEBNext Ultra II RNA Library Prep Kit for Illumina (NEB, E7775). Samples were subjected to qPCR analysis or sequenced on the Illumina NextSeq 500 NGS system as 75 bp pair-ended reads.

The MeRIP-seq data was analyzed as previously described (52). The MeRIP and input library reads were aligned to the mycoplasma genome to assess contamination followed by alignment to the human reference genome (hg19) with STAR. MarkDuplicates (Picard) was used for identifying and removing duplicates reads. DESeq2 was used to call the differential expression genes (DEG) between Ctrlsg and VHL knockout groups. The m6A peaks were called by MeTDiff package with default parameter by combining signals from all the biological triplicates. Homer was used to search for the enriched motif in the m6A peaks, and the m6A peak distribution across the whole transcriptome was called using the Guitar package. A convolutional neural network model (MATK, http://matk.renlab.org/#/home) was used identify the specific m6A site from the detected peaks. Pathway enrichment analysis was performed using Enrichr (https://maayanlab.cloud/Enrichr/enrich).

### RNA immunoprecipitation.

RNA immunoprecipitation (RIP) was performed using a modified native RIP procedure as previously described (53). In brief, approximately 3 × 10^7^ HKC cells were lysed with lysis buffer (100 mM KCl, 5 mM MgCl_2_, 10 mM HEPES pH 7.0, 0.5% NP-40, 1 mM DTT, supplemented with RNase inhibitor and EDTA-free Protease inhibitor before using). The cell lysate was incubated with protein-A/G magnetic beads that immobilized with corresponded antibodies overnight at 4°C. The beads were washed 6 times with NT-2 buffer (50 mM Tris-HCl pH 7.4, 250 mM NaCl, 1 mM MgCl_2_, 0.05% NP-40, 20 mM EDTA, 1 mM DTT, supplemented with RNase inhibitor before using). Input and co-IP RNAs were first digested with Proteinase K buffer (NT-2 buffer supplemented with 1 % SDS and 1.2 mg/mL Proteinase K) at 55°C for 30 minutes and then recovered by TRIzol according to the supplier’s instructions. Samples were analyzed by qPCR and normalized to the input.

### RNA extraction and real-time PCR.

Cellular RNA was extracted using the RNeasy mini kit (Qiagen, 74104) according to the manufacturer’s instructions. cDNA was synthesized with iScript Reverse Transcription Supermix (Bio-Rad, 1708841). cDNA was diluted 10-fold and 2 μL was subjected to qPCR with iTaq Universal SYBR Green Supermix (Bio-Rad, 1725124) on a CFX384 Real-Time PCR System (Bio-Rad). Results were analyzed using the 2−ΔΔCt method. All primers were list in Supplemental Table 2.

### 2D and 3D colony formation assay.

2D colony formation assays were carried out by seeding 6-well plates in triplicate with 2,000 cells/well. The colonies were stained with 5% crystal violet when the cells reached the appropriate density. The 3D soft agar colony formation assays were performed as described previously (54). In brief, 0.4% agar top layer containing specific numbers of cells (10,000 cells for HKC, 30,000 cells for UMRC2 and 786O cells) were applied to 1% agar bottom layer. 0.5 mL medium was added after the top layer became solid then 3 drops of complete medium was added every 4 days. For inhibitor treatment, AKT inhibitor was added the following day after seeding the cells and changed every day. After incubating for 2 weeks (UMRC2 and HKC cells) or 4 weeks (786O cells), cell colonies were stained with 100 μg/mL iodonitrotetrazoliuim chloride (Sigma-Aldrich, I8377) in complete medium overnight. The foci numbers were counted by ImageJ software.

### Luciferase reporter assay.

The fragment of WT and mutant of *PIK3R3*–containing predicted m6A motifs (Supplemental Table 4) were synthesized by Gene Universal and cloned into the XbalI/SalI site of Plenti-tk-luciferase-control (Addgene, 122277). luciferase activities were measured based on a dual-luciferase reporter assay system (Promega, E2920) according to the manufacture’s instruction.

### IHC.

Rabbit polyclonal antibody against PIK3R3 (Cell Signaling Technology, 11889S) or Phospho-AKT (Thr308) PIK3R3 (Cell Signaling Technology, 9275S) were used for IHC staining. The IHC staining was carried out by UT Southwestern Medical Center Tissue Management Shared Resource Core. Briefly, the slides were baked for 20 minutes at 60°C, then deparaffinized and hydrated before the antigen retrieval step. Heat-induced antigen retrieval was performed at pH 9.0 for 20 minutes in a Dako PT Link. The tissue was incubated with a peroxidase block and then an antibody incubation (PIK3R3, 1:100, 60 minutes; pAKT308,1:200, 20 minutes). The PIK3R3 staining was visualized using the EnVision FLEX (Agilent, K8000) visualization system. The pAKT308 staining was visualized using the EnVision+ single reagent Rabbit HRP (Agilent, K400311-2) visualization system. The slides were then dehydrated and mounted with cover glasses. H-Scores for the IHC slides were quantified by pathologist Liwei Jia from department of Pathology at UT Southwestern Medical Center and Sichuan Xi from National Cancer Institute, NIH.

### Statistics.

Statistical analyses were performed with Prism 9.0 software (GraphPad). For experiments comparing 2 sets of data, unpaired 2-tail Student’s *t* test was used. 1-way ANOVA was used for multiple comparison. Survival rates and overall patient survival were analyzed by Kaplan–Meier survival curve. The mouse orthotopic tumor luciferase signal and subcutaneous tumor sized data were presented as mean ± SEM. All other data represent mean ± SD from at least 3 independent experiments. A *P* value of less than 0.05 was considered significant.

### Study approval.

All animal procedures were performed in accordance with the National Institutes of Health guidelines and were approved by the IACUC of the University of Texas Southwestern Medical Center. The deidentified human tissues, which were used for qPCR and IHC assay, have been reviewed by The UT Southwestern Human Research Protection Program (HRPP), which determined that the analysis does not meet the definition of human subject research under 45 CFR 46.102 and therefore does not require IRB approval or oversight.

### Data availability.

MeRIP-Seq and RNA-Seq data have been deposited at GEO (GSE239892) and are publicly available as of the date of publication. Values for all data points are available in the Supporting Data Values file. All other data and reagents relevant to the current study are available from the corresponding authors on reasonable request excluding confidential patient identity information.

## Author contributions

CZ and QZ conceived of and designed the study. CZ, MY and RR acquired data. CZ, AJH, ZZ, RR, LJ, SX, HZ, and QZ analyzed and interpreted data. CZ, JMS, KX, YN, ZL, and QZ wrote and reviewed and/or revised the manuscript. CZ, JZ, LH, JF, HL, QL, CL, PK and QZ performed administrative, technical, or material support duties.

## Supplementary Material

Supplemental data

Unedited blot and gel images

Supporting data values

## Version 1 04/15/2024

Electronic publication

## Version 2 04/19/2024

added grant funding number
