# Supplementary material for: Von Hippel Lindau tumor suppressor controls m6A-dependent gene expression in renal tumorigenesis
Source: J Clin Invest. 2024 Apr 15;134(8):e175703. doi: 10.1172/JCI175703 (PMC11014668; doi:10.1172/JCI175703)
Supplement: Supplemental data [file jci-134-175703-s013.pdf]

Von Hippel Lindau tumor suppressor controls m6A-dependent gene expression in renal tumorigenesis

Cheng Zhang, Miaomiao Yu, Austin J. Hepperla, Zhao Zhang, Rishi Raj, Hua Zhong, Jin Zhou, Lianxin Hu, Jun Fang, Hongyi Liu, Qian Liang, Liwei Jia, Chengheng Liao, Sichuan Xi, Jeremy M. Simon, Kexin Xu, Zhijie Liu, Yunsun Nam, Payal Kapur, Qing Zhang

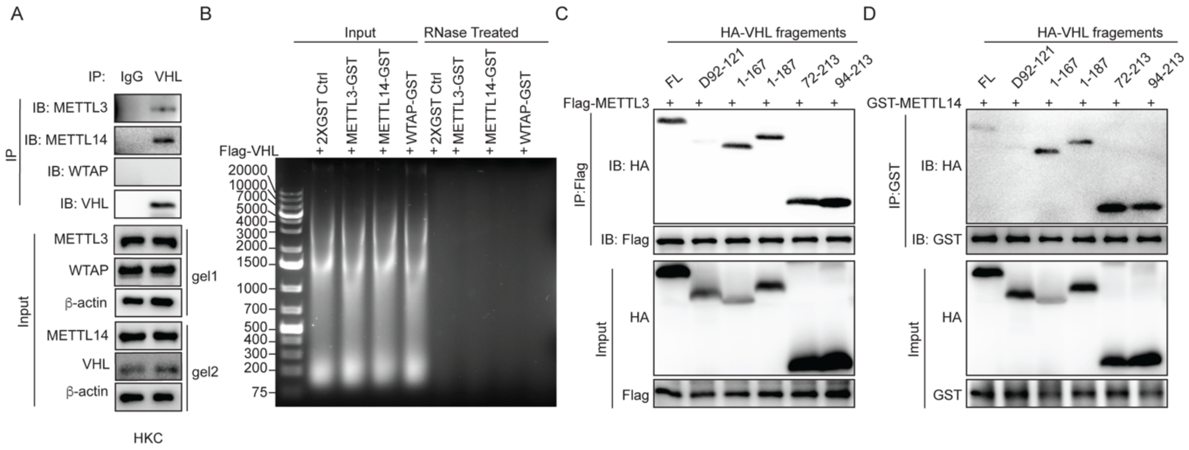

Supplemental Figure 1. VHL interacts with METTL3 and METTL14. (**A**) Endogenous immunoprecipitation assay with VHL antibody. METTL3, METTL14 and WTAP blots were run in parallel using the same biological samples. (**B**) Agarose gel electrophoresis analysis of IVT samples that treated with RNase or not. (**C-D**) Anti-Flag (**C**) or anti-GST (**D**) immunoprecipitation with IVT HA-VHL fragments and Flag-METTL3 or GST-METTL14 protein.

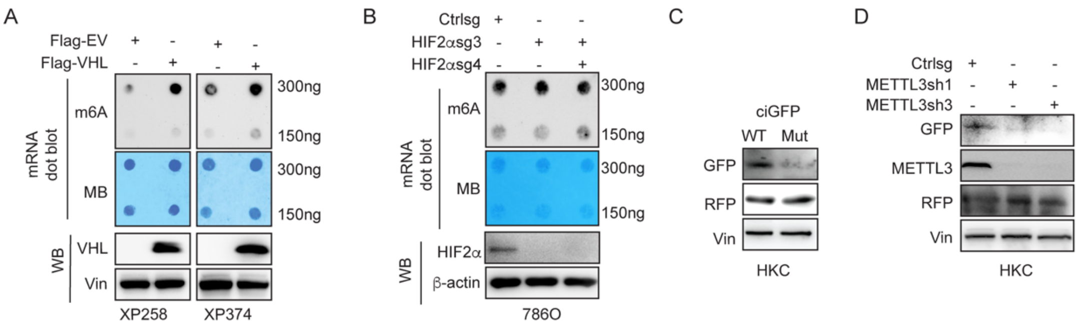

Supplemental Figure 2. VHL regulates m6A modification. (**A**) Dot blot and Western blot assay of samples from indicated PDX cell lines. (**B**) m6A dot blot of mRNA and immunoblot of cell lysate from the 786O cells transduced with lentivirus either expressing control sgRNA (Ctrlsg) or *VHL*sgRNAs (sg1 and sg2). (**C-D**) Immunoblotting analysis of indicated GFP expressing level in HKC cells that transfected with indicated vectors. GFP and RFP blots were run in parallel using the same biological samples.

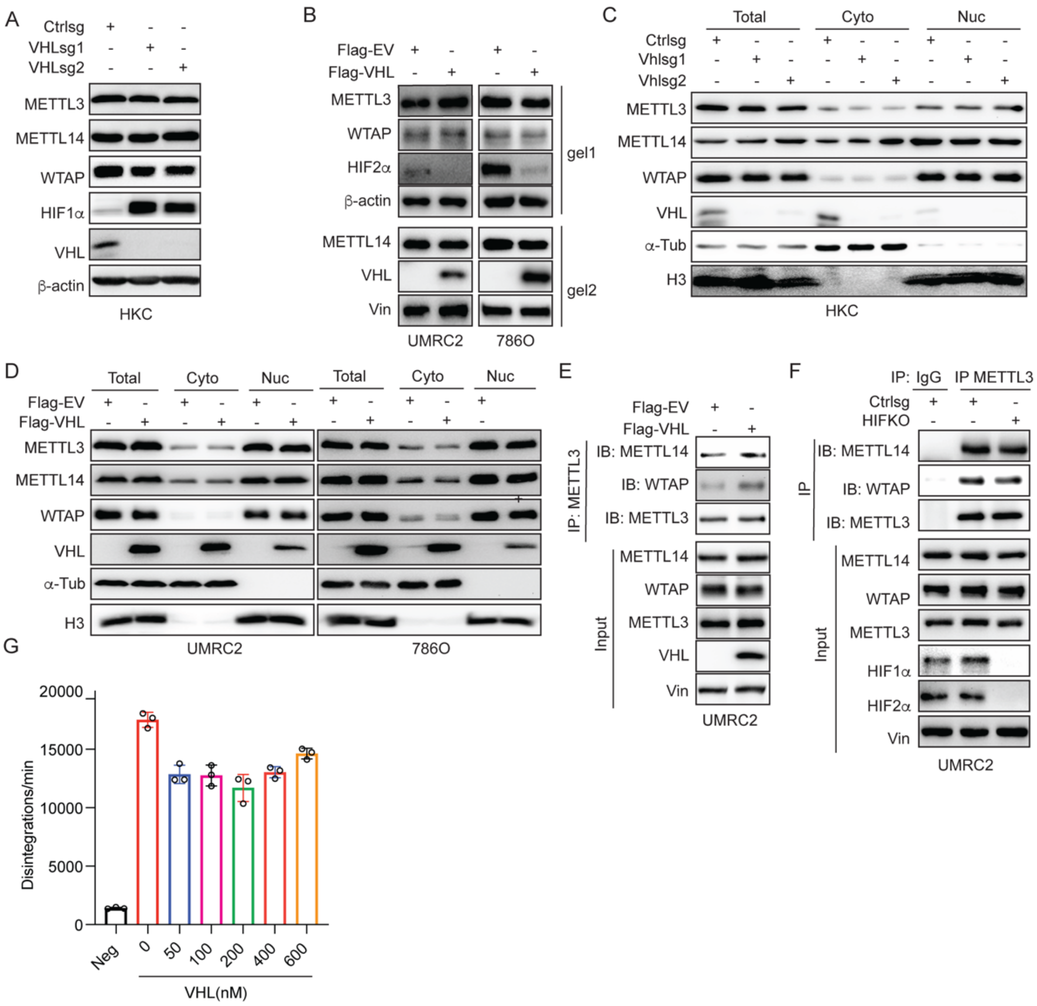

Supplemental Figure 3. VHL regulates the interaction between METTL3 and METTL14. (**A**) Immunoblot of lysate from HKC cells transduced with lentivirus either expressing control sgRNA (Ctrlsg) or *VHL*sgRNAs (sg1 and sg2). (**B**) Immunoblot of lysate from UMRC2 or 786O cells transduced with empty vector or Flag-VHL. METTL3, METTL14 and WTAP blots were run in parallel using the same biological samples. (**C-D**) Western blot analysis with the cellular fractionation samples from the cells that transfected with indicated vectors. (**E-F**) METTL3 immunoprecipitation with samples from UMRC2 cells that transfected with indicated vectors. METTL3, METTL14 and WTAP blots were run in parallel using the same biological samples. (**G**)

In vitro methyltransferase activity of the full-length METTL3/METTL14 complex in present of different amount of purified Flag-VHL protein.

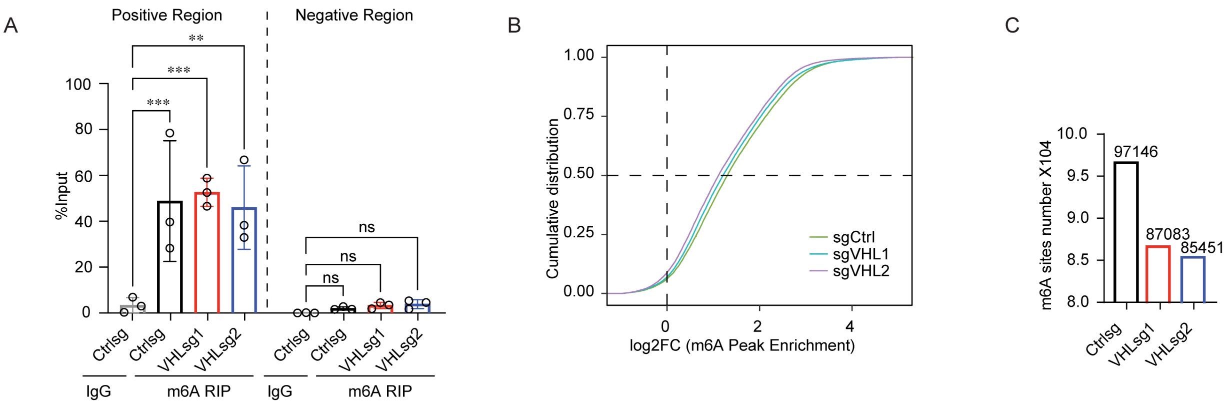

Supplemental Figure 4. Transcriptome-wide identification of different m6A upon VHL depletion. (**A**) MeRIP RNAs were analyzed by qRT-PCR using m6A Positive Control Primers (MeRIP primers human *EEF1A1* positive) and negative control primers (MeRIP primers human *EEF1A1* negative). (**B**) Cumulative distribution of log2 peak intensity of m6A infected with lentivirus encoding Ctrlsg or *VHL*sgRNAs (sg1 and sg2). (**C**) The number of m6A sites identified by MeRIP-seq with the mRNA samples from HKC cells that transduced with lentivirus either expressing control sgRNA (Ctrlsg) or *VHL*sgRNAs (sg1 and sg2).

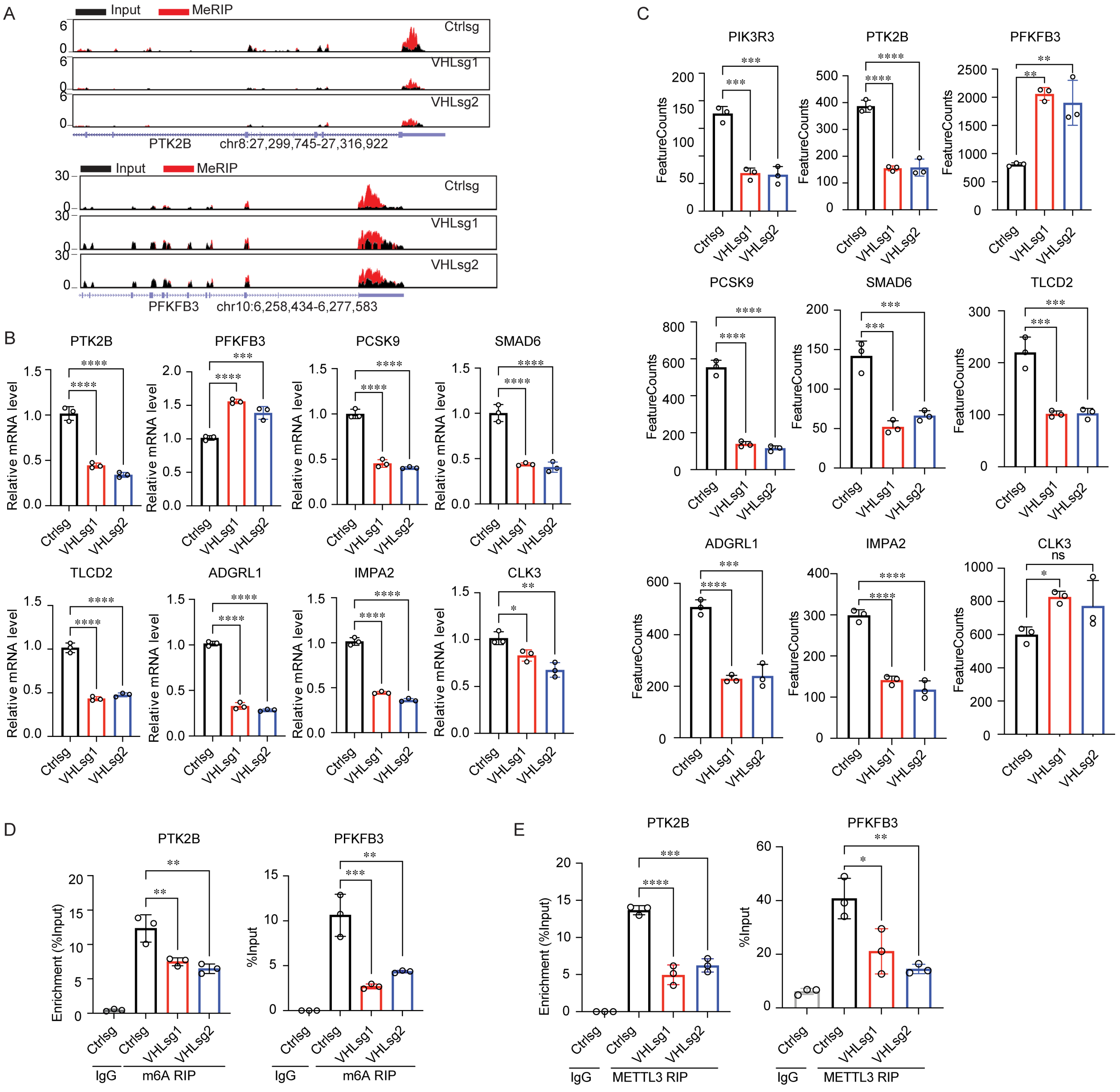

Supplemental Figure 5. Transcriptome-wide RNA-seq and m6A seq assays identify potential targets of VHL involved m6A modification. (**A**) Genome browser representative tracks of three biological replicates displaying the m6A read distribution and changes of *PTK2B* and *PFKFB3* transcripts. (**B**) qPCR analysis of indicated genes RNA levels in HKC Ctrlsg and VHLsg cells. (**C**) Feature counts of indicated genes that identified by RNA-seq with the RNA samples from HKC Ctrlsg and *VHL*sg cells. (**D**) MeRIP-qPCR analysis of *PTK2B* and *PFKFB3* m6A levels in HKC Ctrl sg and VHLsg cells. (**E**) The association of METTL3 with *PTK2B* and

PFKFB3 mRNA was assessed by METTL3 RIP-qPCR analysis. Data show mean ± SD, **P < 0.01, ***P < 0.001, ***P < 0.000, one-way ANOVA analysis.

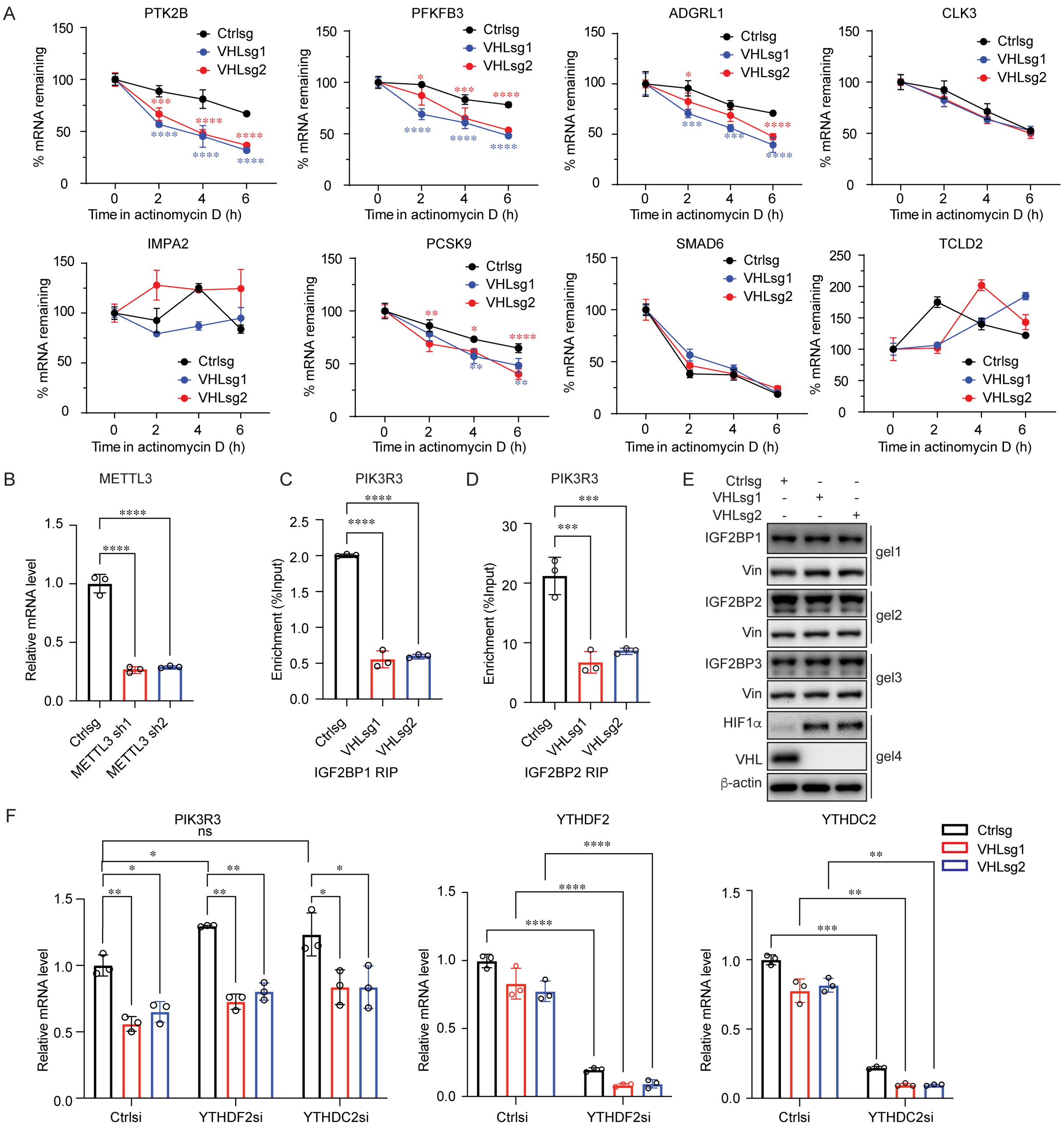

Supplemental Figure 6. VHL regulates mRNA stability in an m6A dependent manner. (**A**) mRNA stability of indicated genes were measured by qPCR in HKC cells transfected with Ctrlsg or VHLsg (sg1 and sg2) and treated with Actinomycin D (5μg/mL) for indicated time. (B) qPCR test the knockdown efficiency of METTL3 in HKC cells. (**C-D**) Endogenous RNA

immunoprecipitation assay test the interaction between *PIK3R3* mRNA and IGF2BP1 (**C**) or IGF2BP2 (**D**). (**E**) Immunoblot of lysate from HKC cells transduced with lentivirus either expressing control sgRNA (Ctrlsg) or *VHL*sgRNAs (sg1 and sg2). HIF1α, IGF2BP1, IGF2BP2 and IGF2BP3 blots were run in parallel using the same biological samples. (**F**) qPCR examine the *PIK3R3*, *YTHDF2* and *YTHDC2* RNA level in HKC cells transfected with indicated siRNAs. Data show mean ± SD, *P < 0.05, **P < 0.01, ***P < 0.001, ****P < 0.0001, ns: not significant, two-way ANOVA (**A** and **F**), one-way ANOVA analysis (**B-D**).

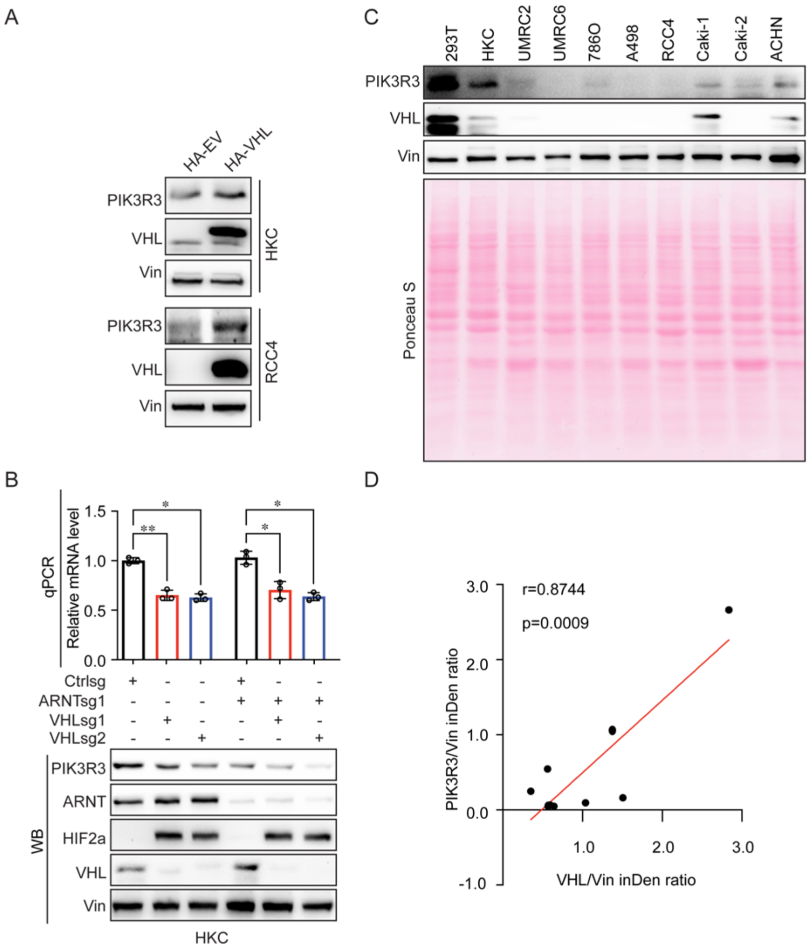

Supplemental Figure 7. VHL positively regulates *PIK3R3* mRNA and protein levels. (**A**) PIK3R3 protein levels in indicated ccRCC cells that transfected with empty or VHL overexpressing vectors were detected by Western blot. (**B**) *PIK3R3* mRNA and protein levels in indicated HKC

cells that transfected with indicated vectors were detected by qPCR and Western blot respectively. (**C**) Immunoblotting for lysates from indicated renal cell lines. (**D**) Pearson's correlation analysis of quantified protein level between VHL and PIK3R3 in renal cell lines. Data show mean ± SD, *P < 0.05, **P < 0.01, two-way ANOVA.

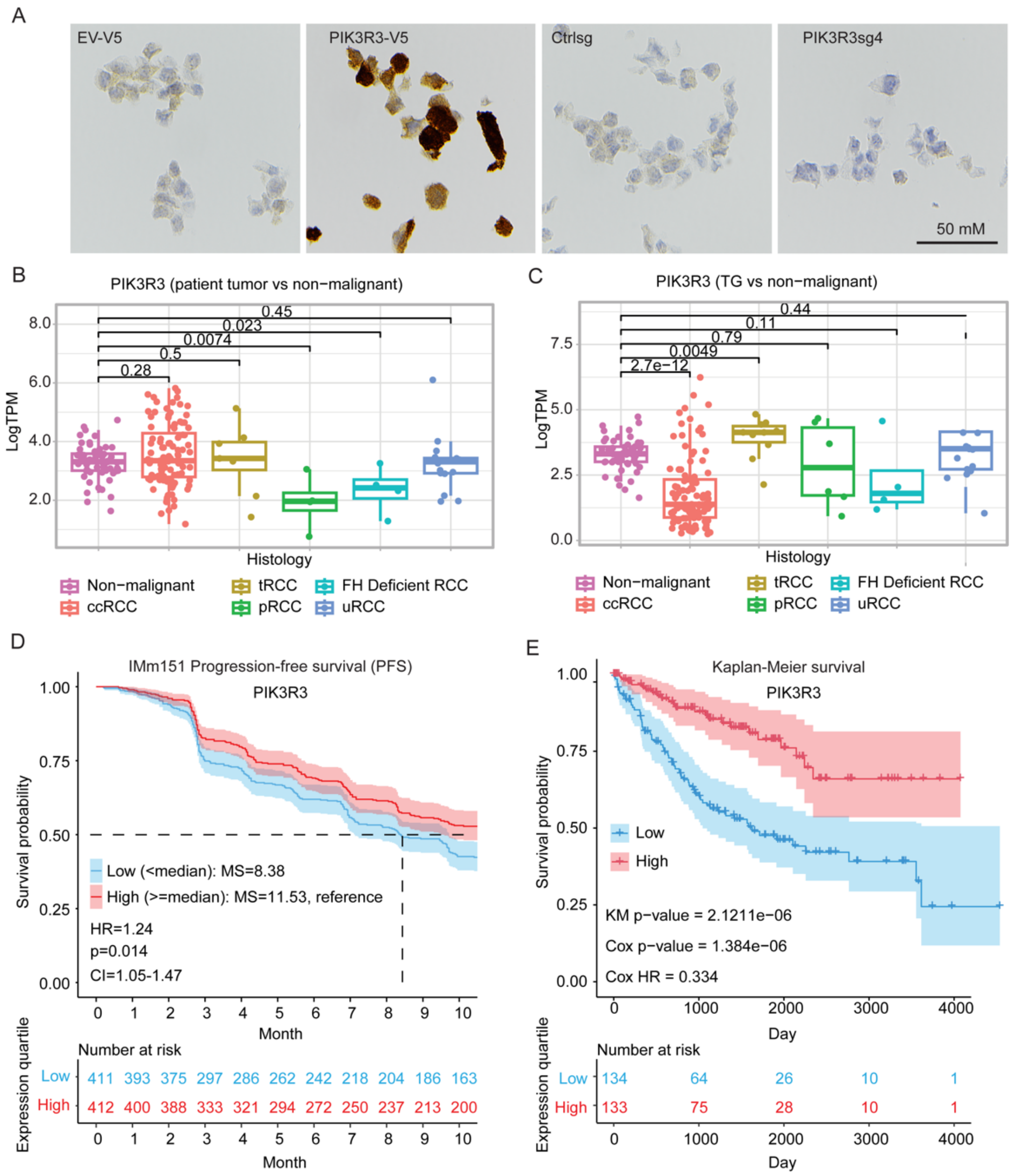

Supplemental Figure 8. PIK3R3 is downregulated in human ccRCC. (**A**) Representative images for PIK3R3 immunohistochemical staining in the HKC cells transduced with indicated vectors. (**B-C**) *PIK3R3* transcription in IMmotion151 clinical subtypes (**B**) and patient-derived

tumorgrafts (**C**) were detect by RNA-seq. (**D**) IMmotion151 clinical subtypes progression-free survival curves based on PIK3R3 gene expression. (**E**) TCGA-KIRC Kaplan-Meier survival curves for *PIK3R3* gene expression divided in quartiles.

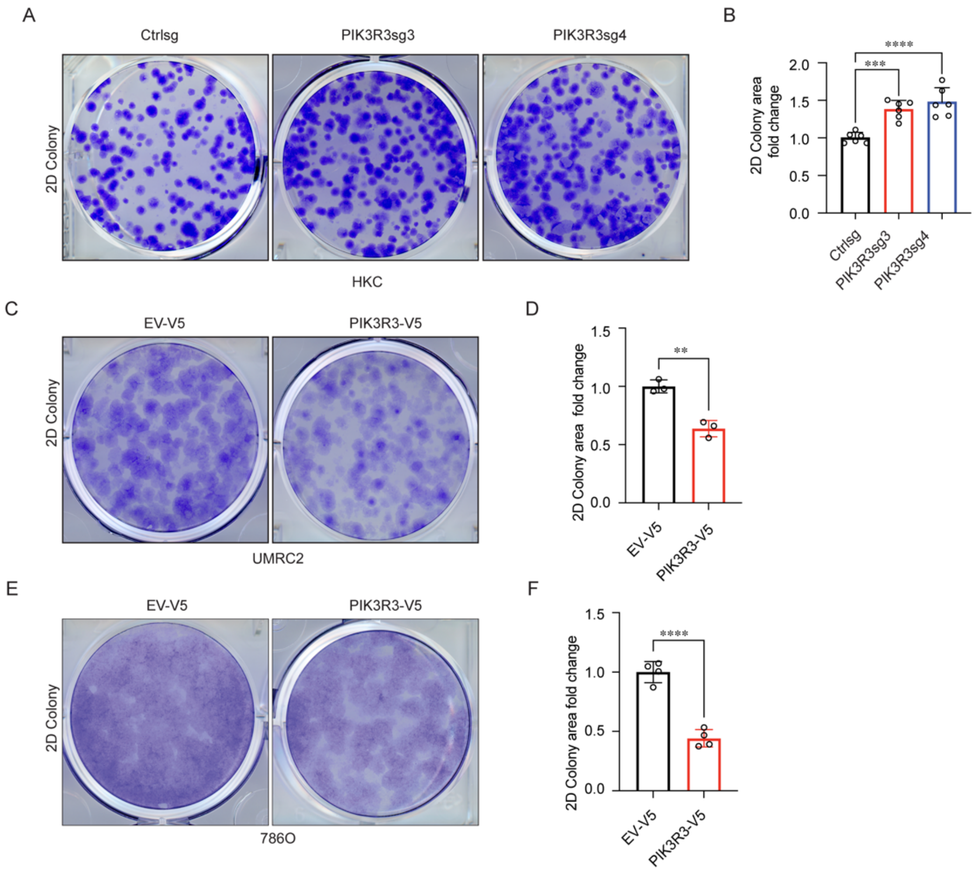

Supplemental Figure 9. **PIK3R3 suppresses tumor growth *in vitro.*** (**A-B**) 2D colony formation assay (**A**), and corresponding quantification data (n = 6) (**B**) in HKC cell lines transduced with indicated sgRNAs. (**C-F**) 2D colony formation assay (**C and E**), and corresponding quantification data (n = 3) (**D** and **F**) in UMRC2 or 786O cell lines transduced with indicated overexpressing vectors. Data show mean ± SD, **P < 0.01, ***P < 0.001, ****P < 0.0001, ns: not significant, one-way ANOVA analysis (**B**) or unpaired t-test (**D** and **F**)

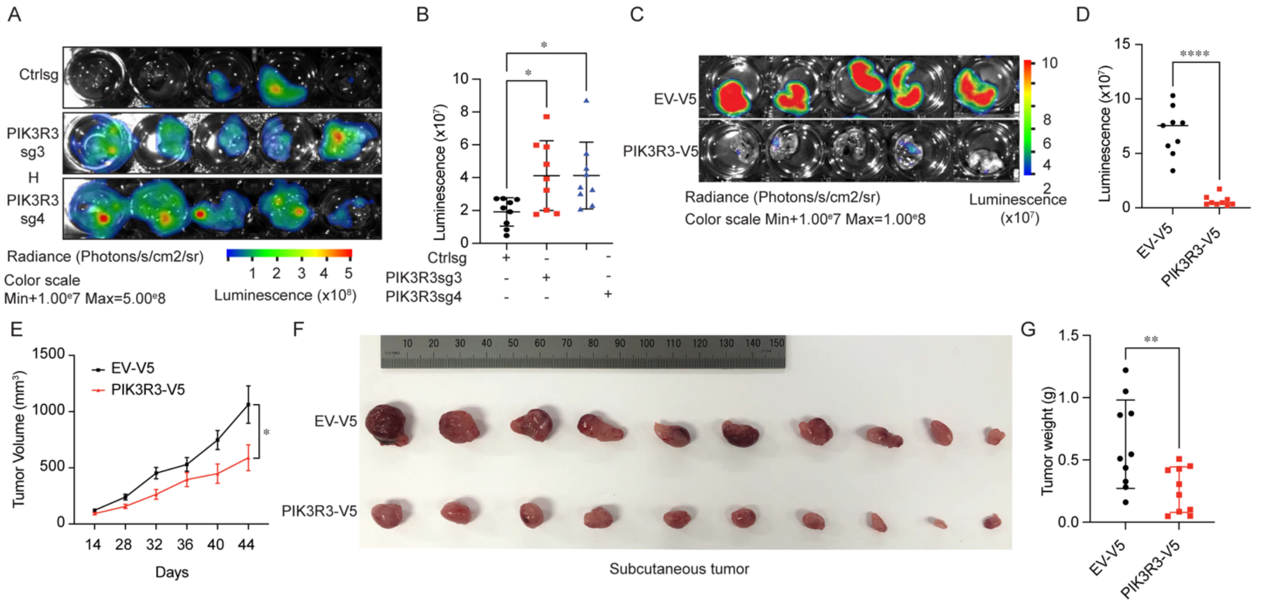

Supplemental Figure 10. PIK3R3 suppresses tumor growth *in vivo.* (**A**-**D**) Representative lung *ex vivo* bioluminescence imaging (**A** and **C**) of mice renal sub-capsule orthotopic injected with indicated vectors transduced HKC luciferase cell lines and corresponding quantitation data of bioluminescence signal (B and D). (**E-G**) Tumor volume (**E**), representative tumors images (**F**) and tumor weight (**G**) after dissection of mice that subcutaneously injected with empty vector or PIK3R3-V5 transduced UMRC2 cells. Data show mean ± SD; *P < 0.05, **P < 0.01, ***P < 0.001, ****P < 0.0001; one-way ANOVA (**B**), unpaired two-tailed t test (**D,** and **G**) or two-way ANOVA (**E**).

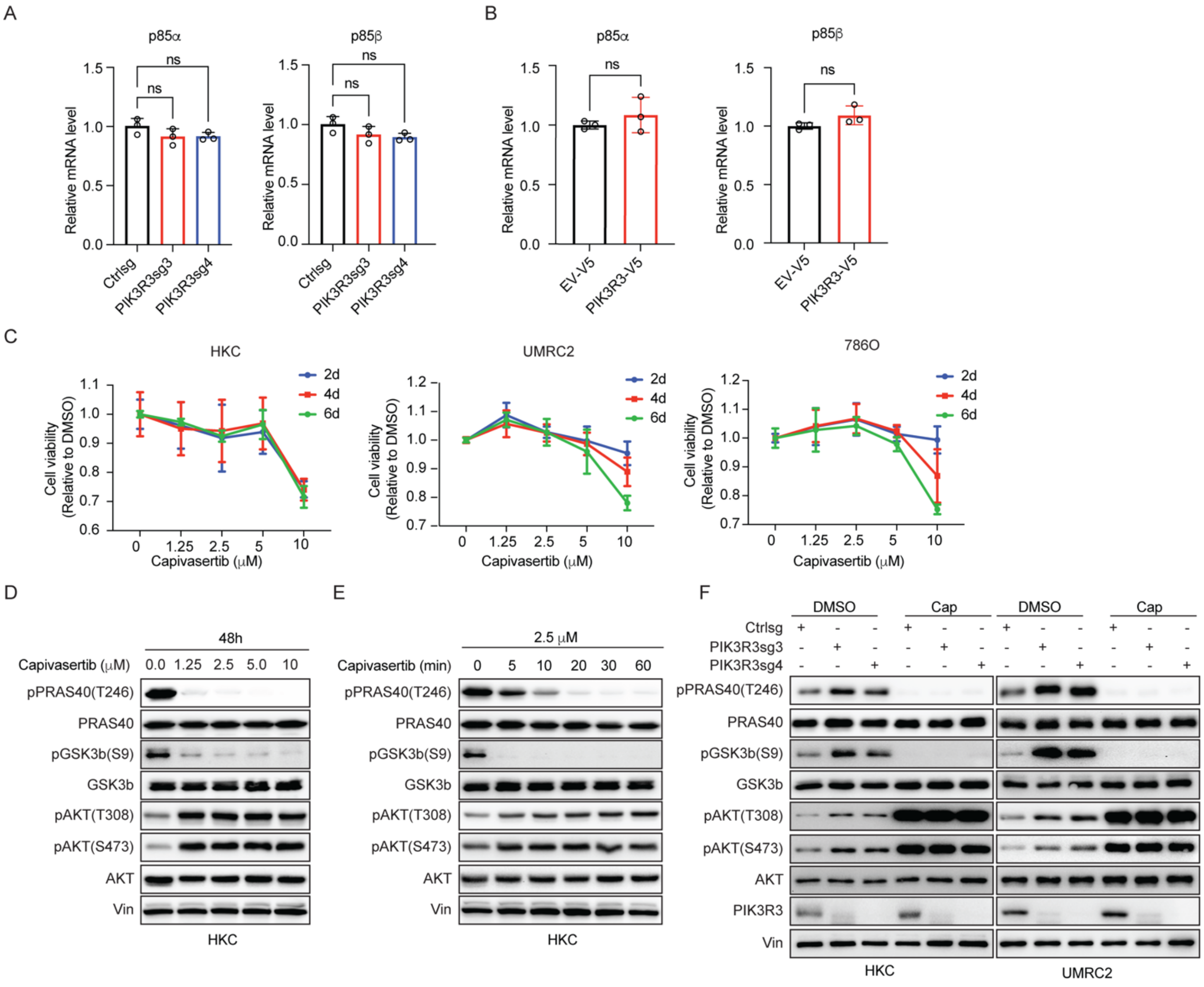

Supplemental Figure 11. PIK3R3 suppresses AKT activation. (**A-B**) qPCR test the RNA level of *p85a* and *p85b* in VHL knockout (**A**) or overexpression (**B**) HKC cells. (**C**) Cell viability of renal cells treated with different amount of Capivasertib (Cap), and different time as indicated. (**D**-**E**) Immunoblot of lysate from HKC cells treated with indicated concentration of Capivasertib for 48 hours (**D**) or treated with 2.5 μM Capivasertib for indicated time (**E**). (**F**) Immunoblot of lysate from PIK3R3 knockout HKC or UMRC2 cells that treated with DMSO or Capivasertib. Data show mean ± SD; ns: not significant, one-way ANOVA (**A and B**), unpaired two-tailed t test (**C** and **D**).

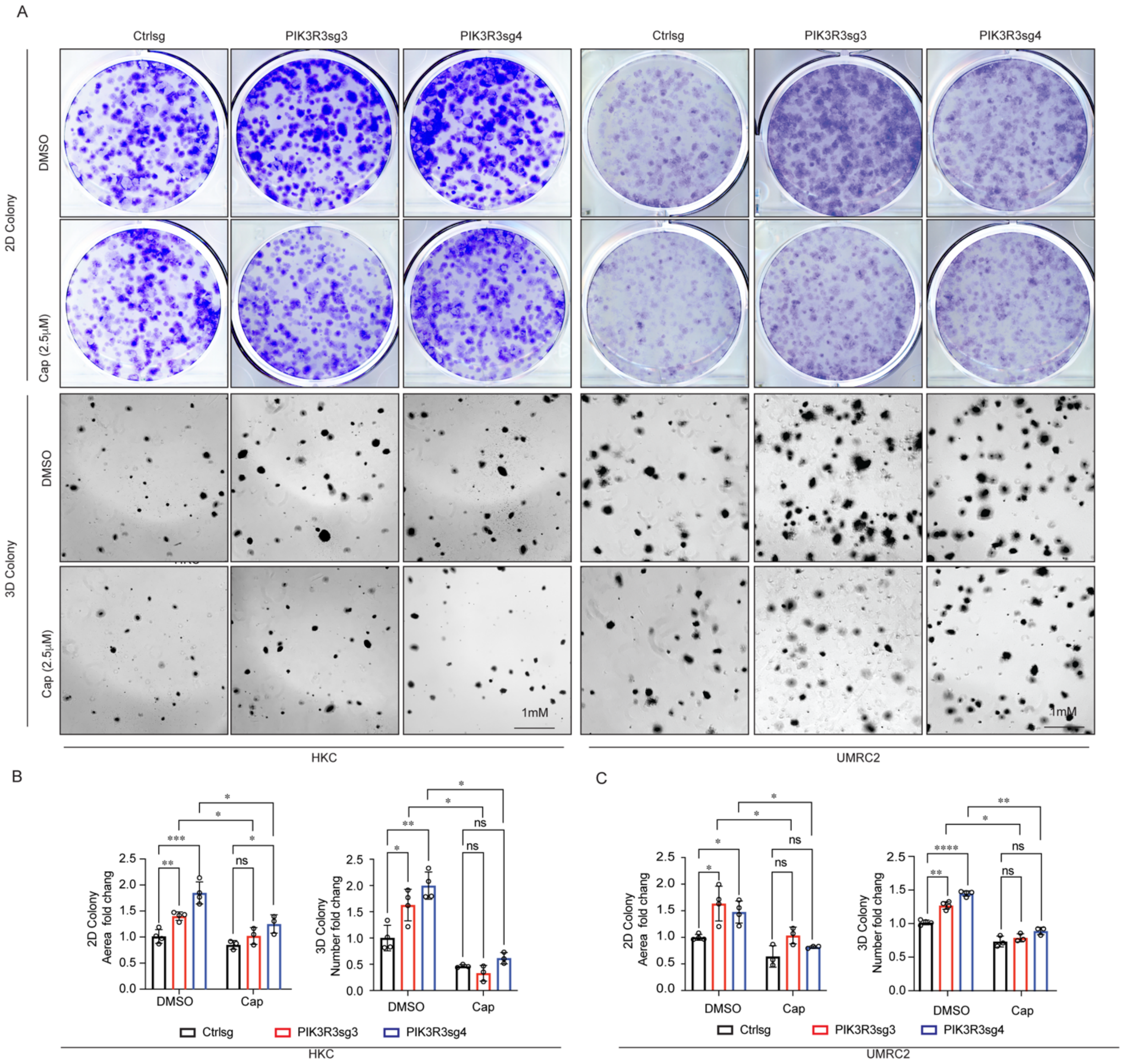

Supplemental Figure 12. AKT inhibitor reveres the PIK3R3 knockout induced tumor cells growth. (**A**) 2D and 3D colony formation assay in HKC or UMRC2 (VHL expressing) cell lines transduced with indicated sgRNAs and followed by treatment with Capivasertib (2.5 μM). (**B-C**) Corresponding quantification data (n = 3) of 2D and 3D colony formation assay. Data show mean ± SD; *P < 0.05, **P < 0.01, ***P < 0.001, ****P < 0.0001, ns: not significant, two-way ANOVA.

Supplemental Table1: sgRNAs, shRNAs and siRNAs

**sgRNA**	**Sequence**
VHLsg1	CATACGGGCAGCACGACGCG
VHLsg2	GCGATTGCAGAAGATGACCT
PIK3R3sg1	GGATGCAGAATGGTACTGGG
PIK3R3sg2	CGATGACGCAGACTGGAGGG
PIK3R3sg3	CCCTCCAGTCTGCGTCATCG
PIK3R3sg4	TGAGGCATCTCGGACCAAGA
P85αsg2	GTGATTATACTCTTACACTA
P85αsg3	TTTCCTAGATACACCCTCCG
P85βsg1	GCTGCCGTGTGCGCTCGTTG
P85βsg3	AGACTGGTCCCTGAGCGACG
HIF1αsg1	CCATCAGCTATTTGCGTGTG
HIF2αsg1	AATCTCCTCATGGTCGCA
HIF2αsg2	TCATGAGGATGAAGTGCA
ARNTsg1	TTGGCAGTAGTCGCCGCCA
ARNTsg2	GTGGCATCTGCGGCCATGG
**shRNAs**	**Sequence**
METTL3sh1	CAAGGAACAATCCATTGTT
METTL3sh2	AAGTATGTTCACTATGAAA
**siRNAs**	**ThermoFisher Cat#**
IGF2BP1si pool	s20916; s20917; s20918
IGF2BP2si pool	s20922; s20923; s20924
IGF2BP3si pool	S20919; s20920; s20921

Supplemental Table 2: Primers' sequence

**Primers for qPCR**			
**Primer**	**Sequence**	**Primer**	**Sequence**
GAPDH-F	TCAAGGCTGAGAACGGGAAG	IGF2BP3-F	CTTCTATGCTTGCCAGGTTGC
GAPDH-R	GGACTCCACGACGTACTCAG	IGF2BP3-R	GAGCCTTCTGTTGTTGGTGC
hVHL-F	GGAGCCTAGTCAAGCCTGAGA	PCSK9-F	GACGATGCCTGCCTCTACTC
hVHL-R	CATCCGTTGATGTGCAATGCG	PCSK9 -R	CCAATGATGTCCTCCCCTGG
METTL3-F	TTGTCTCCAACCTTCCGTAGT	SMD6-F	TTGCAACCCCTACCACTTCA
METTL3-R	CCAGATCAGAGAGGTGGTGTAG	SMD6-R	TTGGTGGCATCTGGAGACAT
P85a-F	TGGACGGCGAAGTAAAGCATT	TCLD2-F	CTGCAGCGCAAACCCTAAAG
P85a-R	AGTGTGACATTGAGGGAGTCG	TCLD2-R	GCATCTCAGTTGACACCCCT
P85b-F	GGGGACATTTCAAGGGAGGA	ADGRL1-F	CGATGCTGACCCTTTCCAGA
P85b-R	ACAACGGAGCAGAAGGTGAG	ADGRL1-R	TCTGCTCCACTTTGTAGGGG
PIK3R3-F	TGAGGAAGATGAAAACCTGCCCCA	IMPA2-F	GAAACCTCTCTCGCAACTCAG
PIK3R3-R	CCATCGGCCACCACAGAGCAAG	IMPA2-R	GGGCAGGACAGATCATCAGAA
PTK2B-F	AATGCACTTGACAAGAAGTC	CLK3-F	CGTACCTGAGCTACCGATGGA
PTK2B-R	GCTTTAAGTTCTCCTGCATC	CLK3-R	TCCCTTCGGGACGGGTATC
IGF2BP1-F	ATAGCTCCTTTATGCAGGCTC	SMD6-F	CACTGAAACGGAGGCTACCAAC
IGF2BP1-R	CGGGTGGTGCAATCTTGATG	SMD6-R	CCTGGTCGTACACCGCATAGAG
IGF2BP2-F	AAACATCCCTCCTCACCTGC		
IGF2BP2-R	GTGTCTGTGTTGACTTGTTCC		
**Primers for RIP-qPCR**			
**Primer**	**Sequence**		
PIK3R3-ripF	TGCTCGGCCTCTCCACTTCACA	Myc-ripF	GCATACATCCTGTCCGTCCA
PIK3R3-ripR	ACGTCAGGCTTGCCTACCCTGT	Myc-ripR	GTCGTTTCCGCAACAAGTCC
PFKFB3-ripF	ATCTTGACCTGGGAAGACGC	PTK2B-ripF	GCCTTGCTGTTGGTCATGTG
PFKFB3-ripR	CCTTGCACATCTTTGGAGGC	PTK2B-ripR	GTGCAAAGTGGCAAGGAAG

Supplemental Table 3: Antibodies and beads

**Antibodies**				
**Name**	**Vendor**	**Cat#**	**Dilution**	**References**
Mouse anti-alpha-Tubulin	Cell Signaling Technology	Cat# 3873; RRID: AB_1904178	WB: 1:2000	(1)
Rabbit anti-VHL for WB	Cell Signaling Technology	Cat# 68547; RRID: AB_2716279	WB: 1:1000	(1)
Mouse anti-VHL for WB	Santa Cruz Biotechnology	Cat# sc-17780, RRID:AB_628435	WB: 1:500	(2)
Mouse anti-VHL for Co-IP	BD Biosciences	Cat# c, RRID:AB_396376	Co-IP: 1:100	(3)
Rabbit anti-Mettl3	Abcam	Cat# ab195352, RRID:AB_2721254	WB: 1:1000; Co-IP: 1:100	(4)
Rabbit anti-Mettl14	Sigma-Aldrich	Cat# HPA038002, RRID:AB_10672401	WB: 1:1000; Co-IP: 1:100	(5)
Rabbit anti-WTAP	Proteintech	Cat# 10200-1-AP, RRID:AB_2216349	WB: 1:2000	(6)
Mouse anti-beta-Actin	Cell Signaling Technology	Cat# 3700, RRID:AB_2242334	WB: 1:2000	(7)
Rabbit anti-GST-Tag	Cell Signaling Technology	Cat# 2625, RRID:AB_490796	WB: 1:1000	(1)
Rabbit anti-HIF1alpha	Cell Signaling Technology	Cat# 14179, RRID:AB_2622225	WB: 1:1000	(1)
Rabbit anti-HIF2alpha	Cell Signaling Technology	Cat# 7096, RRID:AB_10898028	WB: 1:1000	(1)
Mouse anti-ARNT1/HIF1beta	BD Biosciences	Cat# 611078; RRID: AB_398391	WB: 1:1000	(1)
Rabbit anti-m6A	Synaptic Systems	Cat# 202 003, RRID:AB_2279214	Dot blot: 1:1000; RIP: 1:100	(5)
Rabbit anti-GFP	Proteintech	Cat# 50430-2-AP, RRID:AB_11042881	WB: 1:1000	(8)
Rabbit anti-RFP	Proteintech	Cat# 67378-1-Ig, RRID:AB_2882625	WB: 1:1000	(9)
Mouse anti-Vinculin	Sigma-Aldrich	Cat# V9131, RRID:AB_477629	WB: 1:4000	(1)
Rabbit anti-PIK3R3	Cell Signaling Technology	Cat# 11889, RRID:AB_2797756	WB: 1:1000; IHC 1:100	(10)
Mouse anti-PIK3R3	R&D systems	MAB6638-SP	WB: 1:1000	(11)
Rabbit antib-IGF2BP1	Cell Signaling Technology	Cat# 8482, RRID:AB_11179079	WB: 1:1000	(12)
Rabbit antib-IGF2BP2	Proteintech	Cat# 11601-1-AP, RRID:AB_2122672	WB: 1:1000	(13)
Rabbit antib-IGF2BP3	Proteintech	Cat# 14642-1-AP, RRID:AB_2122782	WB: 1:1000	(13)
Rabbit anti-AKT(pan)	Cell Signaling Technology	Cat# 4691, RRID:AB_915783	WB: 1:1000	(14)
Rabbit anti-Phospho-Akt (Thr308)	Cell Signaling Technology	Cat# 2965, RRID:AB_2255933	WB: 1:1000	(14)

Rabbit anti-Phospho-Akt (Thr473)	Cell Signaling Technology	Cat# 9271, RRID:AB_329825	WB: 1:1000	(15)
Rabbit anti-V5-Tag	Cell Signaling Technology	Cat# 13202; RRID: AB_2687461	WB: 1:1000	(7)
Rabbit Anti-p110alpha	Cell Signaling Technology	Cat# 4249, RRID:AB_2165248	WB: 1:1000	(16)
Rabbit Anti- p110beta	Cell Signaling Technology	Cat# 3011, RRID:AB_2165246	WB: 1:1000	(16)
Rabbit anti-p85	Cell Signaling Technology	Cat# 4257, RRID:AB_659889	WB: 1:1000	(17)
Rabbit anti-p85alpha	Proteintech	Cat# 60225-1-Ig, RRID:AB_11042594	WB: 1:1000	(18)
Rabbit Anti- p85beta	Abcam	Cat# ab180967	WB: 1:1000	(19)
Mouse anti-Ub	Santa Cruz Biotechnology	Cat# sc-8017; RRID: AB_628423	WB: 1:200	(1)
Rabbit Anti- PRAS40	Cell Signaling Technology	Cat# 2691; RRID: AB_2225033	WB: 1:1000	(20)
Rabbit Anti-pPRAS40(T246)	Cell Signaling Technology	Cat# 2997; RRID: AB_2258110	WB: 1:1000	(20)
Rabbit Anti- GSK3β	Cell Signaling Technology	Cat# 12456; RRID: AB_2636978	WB: 1:1000	(21)
Rabbit Anti-pGSK3β(S9)	Cell Signaling Technology	Cat# 9336; RRID: AB_331405	WB: 1:1000	(21)
Normal Rabbit IgG	Cell Signaling Technology	Cat# 2729; RRID: AB_1031062		(1)
Normal Mouse IgG	Santa Cruz Biotechnology	Cat# sc-2025, RRID:AB_737182		(1)
HRP-conjugated goat anti-mouse	Thermo Fisher Scientific	Cat# 31430; RRID: AB_228307	WB: 1:5000	(1)
HRP-conjugated goat anti-rabbit IgG	Thermo Fisher Scientific	Cat# 31460; RRID: AB_228341	WB: 1:5000	(1)
**Beads**				
Rat anti-HA affinity matrix	Roche	Cat# 11815016001; RRID: AB_390914		(7)
Mouse anti-FLAG M2 Affinity Gel	Sigma-Aldrich	Cat# A2220, RRID: AB_10063035		(7)
Glutathione Sepharose® 4B	Sigma-Aldrich	Cat# GE17-0756-01		(1)
Protein G Agarose	Sigma-Aldrich	Cat# 11243233001		(22)
Protein A/G Magnetic Beads	Thermo Scientific	Cat# 88802		(23)

1. Hu L, Xie H, Liu X, Potjewyd F, James LI, Wilkerson EM, et al. TBK1 Is a Synthetic Lethal Target in Cancer with VHL Loss. *Cancer Discov.* 2020;10(3):460-75.
2. Hu L, Wu H, Jiang T, Kuang M, Liu B, Guo X, et al. pVHL promotes lysosomal degradation of YAP in lung adenocarcinoma. *Cell Signal.* 2021;83:110002.

3. Li S, Li W, Yuan J, Bullova P, Wu J, Zhang X, et al. Impaired oxygen-sensitive regulation of mitochondrial biogenesis within the von Hippel-Lindau syndrome. *Nat Metab.* 2022;4(6):739-58.
4. Guan H, Tian K, Luo W, and Li M. m(6)A-modified circRNA MYO1C participates in the tumor immune surveillance of pancreatic ductal adenocarcinoma through m(6)A/PD-L1 manner. *Cell Death Dis.* 2023;14(2):120.
5. Lin S, Choe J, Du P, Triboulet R, and Gregory RI. The m(6)A Methyltransferase METTL3 Promotes Translation in Human Cancer Cells. *Mol Cell.* 2016;62(3):335-45.
6. Gao XQ, Zhang YH, Liu F, Ponnusamy M, Zhao XM, Zhou LY, et al. The piRNA CHAPIR regulates cardiac hypertrophy by controlling METTL3-dependent N(6)-methyladenosine methylation of Parp10 mRNA. *Nat Cell Biol.* 2020;22(11):1319-31.
7. Zhou J, Simon JM, Liao C, Zhang C, Hu L, Zurlo G, et al. An oncogenic JMJD6-DGAT1 axis tunes the epigenetic regulation of lipid droplet formation in clear cell renal cell carcinoma. *Mol Cell.* 2022;82(16):3030-44 e8.
8. Shen C, Li R, Negro R, Cheng J, Vora SM, Fu TM, et al. Phase separation drives RNA virus-induced activation of the NLRP6 inflammasome. *Cell.* 2021;184(23):5759-74 e20.
9. Che R, Liu C, Wang Q, Tu W, Wang P, Li C, et al. The Valsa Mali effector Vm1G-1794 protects the aggregated MdEF-Tu from autophagic degradation to promote infection in apple. *Autophagy.* 2023;19(6):1745-63.
10. Ali SR, Humphreys KJ, Simpson KJ, McKinnon RA, Meech R, and Michael MZ. Functional high-throughput screen identifies microRNAs that promote butyrate-induced death in colorectal cancer cells. *Mol Ther Nucleic Acids.* 2022;30:30-47.
11. Tourette C, Li B, Bell R, O'Hare S, Kaltenbach LS, Mooney SD, et al. A large scale Huntingtin protein interaction network implicates Rho GTPase signaling pathways in Huntington disease. *J Biol Chem.* 2014;289(10):6709-26.
12. Huang H, Weng H, Sun W, Qin X, Shi H, Wu H, et al. Recognition of RNA N(6)-methyladenosine by IGF2BP proteins enhances mRNA stability and translation. *Nat Cell Biol.* 2018;20(3):285-95.
13. Peng F, Xu J, Cui B, Liang Q, Zeng S, He B, et al. Oncogenic AURKA-enhanced N(6)-methyladenosine modification increases DROSHA mRNA stability to transactivate STC1 in breast cancer stem-like cells. *Cell Res.* 2021;31(3):345-61.
14. Guo J, Chakraborty AA, Liu P, Gan W, Zheng X, Inuzuka H, et al. pVHL suppresses kinase activity of Akt in a proline-hydroxylation-dependent manner. *Science.* 2016;353(6302):929-32.
15. Ryu S, Spadaro O, Sidorov S, Lee AH, Caprio S, Morrison C, et al. Reduction of SPARC protects mice against NLRP3 inflammasome activation and obesity. *The Journal of clinical investigation.* 2023;133(19).
16. Takahashi M, Okamoto Y, Kato Y, Shirahama H, Tsukahara S, Sugimoto Y, et al. Activating mutations in EGFR and PI3K promote ATF4 induction for NSCLC cell survival during amino acid deprivation. *Heliyon.* 2023;9(4):e14799.
17. Sp N, Kang DY, Jo ES, Lee JM, Bae SW, and Jang KJ. Pivotal Role of Iron Homeostasis in the Induction of Mitochondrial Apoptosis by 6-Gingerol Through PTEN Regulated PD-L1 Expression in Embryonic Cancer Cells. *Front Oncol.* 2021;11:781720.
18. Pi S, Mao L, Chen J, Shi H, Liu Y, Guo X, et al. The P2RY12 receptor promotes VSMC-derived foam cell formation by inhibiting autophagy in advanced atherosclerosis. *Autophagy.* 2021;17(4):980-1000.
19. Lin J, Zeng C, Zhang J, Song Z, Qi N, Liu X, et al. EFNA4 promotes cell proliferation and tumor metastasis in hepatocellular carcinoma through a PIK3R2/GSK3beta/beta-catenin positive feedback loop. *Mol Ther Nucleic Acids.* 2021;25:328-41.

20. Shimobayashi M, Thomas A, Shetty S, Frei IC, Wolnerhanssen BK, Weissenberger D, et al. Diet-induced loss of adipose hexokinase 2 correlates with hyperglycemia. *Elife.* 2023;12.

21. Chan GKL, Maisel S, Hwang YC, Pascual BC, Wolber RRB, Vu P, et al. Oncogenic PKA signaling increases c-MYC protein expression through multiple targetable mechanisms. *Elife.* 2023;12.

22. Zhang J, Wu T, Simon J, Takada M, Saito R, Fan C, et al. VHL substrate transcription factor ZHX2 as an oncogenic driver in clear cell renal cell carcinoma. *Science.* 2018;361(6399):290-5.

23. Petrosino JM, Hinger SA, Golubeva VA, Barajas JM, Dorn LE, Iyer CC, et al. The m(6)A methyltransferase METTL3 regulates muscle maintenance and growth in mice. *Nat Commun.* 2022;13(1):168.

Supplemental Table 4: PIK3R3 3'UTR WT and mutant sequence

Name	Sequence	Comment
**PIK3R3 3'UTR[wt]**	AGAGGAAGTGGGAAGAGAGGTGGTTCTCTGGCATTTT<br>TTTCTACAGTTTTTATTAGACTACGATGAGGGCATTCT<br>TTCTACATAGACTGCTTGTTTTGCACAAGAAGTGATTT<br>TGTGAATGTGAAGTGGAGAGGCCGAGCAGCAGCCGG<br>CCGGGATGGGGGCATTAGAGGCCTGAGGTTCTCTAG<br>GACTCAGCCATGCCGCTGCACTGACATACTAAGCTGG<br>AAGCAGATGTTTTTTTTG	Red highlighted "**A**" is the predicated m6A modification site
**PIK3R3 3'UTR[mut]**	AGAGGAAGTGGGAAGAGAGGTGGTTCTCTGGCATTTT<br>TTTCTACAGTTTTTATTAGCCTACGATGAGGGCATTCT<br>TTCTACATAGCCTGCTTGTTTTGCACAAGAAGTGATTT<br>TGTGAATGTGAAGTGGAGAGGCCGAGCAGCAGCCGG<br>CCGGGATGGGGGCATTAGAGGCCTGAGGTTCTCTAG<br>GCCTCAGCCATGCCGCTGCACTGACATACTAAGCTGG<br>AAGCAGATGTTTTTTTTG	The predicated m6A sites of WT PIK3R3 3'UTR were mutated to "**C**"
