# Supplementary material for: Von Hippel Lindau tumor suppressor controls m6A-dependent gene expression in renal tumorigenesis
Source: J Clin Invest. 2024 Apr 15;134(8):e175703. doi: 10.1172/JCI175703 (PMC11014668; doi:10.1172/JCI175703)

# Full unedited gel for Figure 1

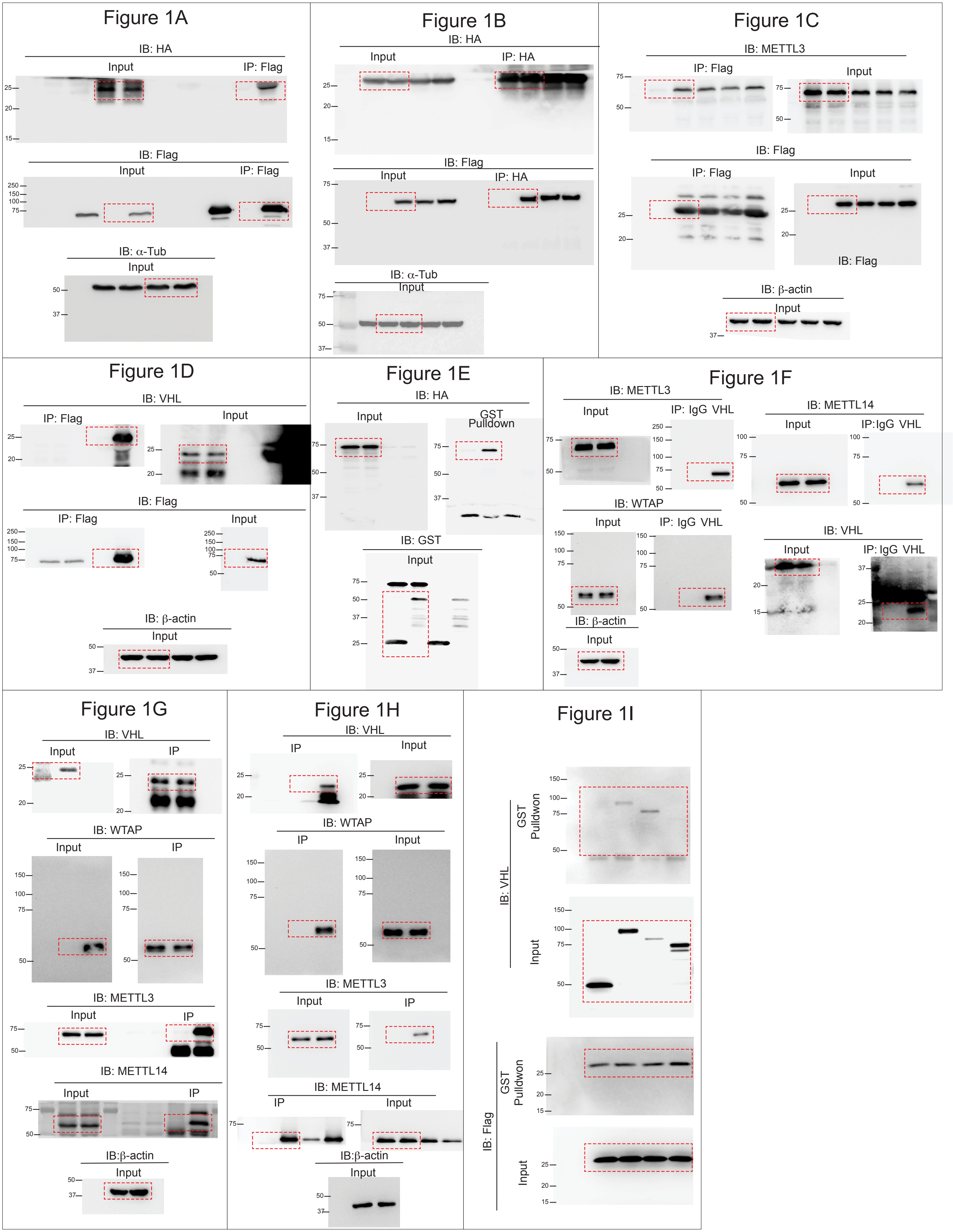

# Full unedited gel for Figure 2

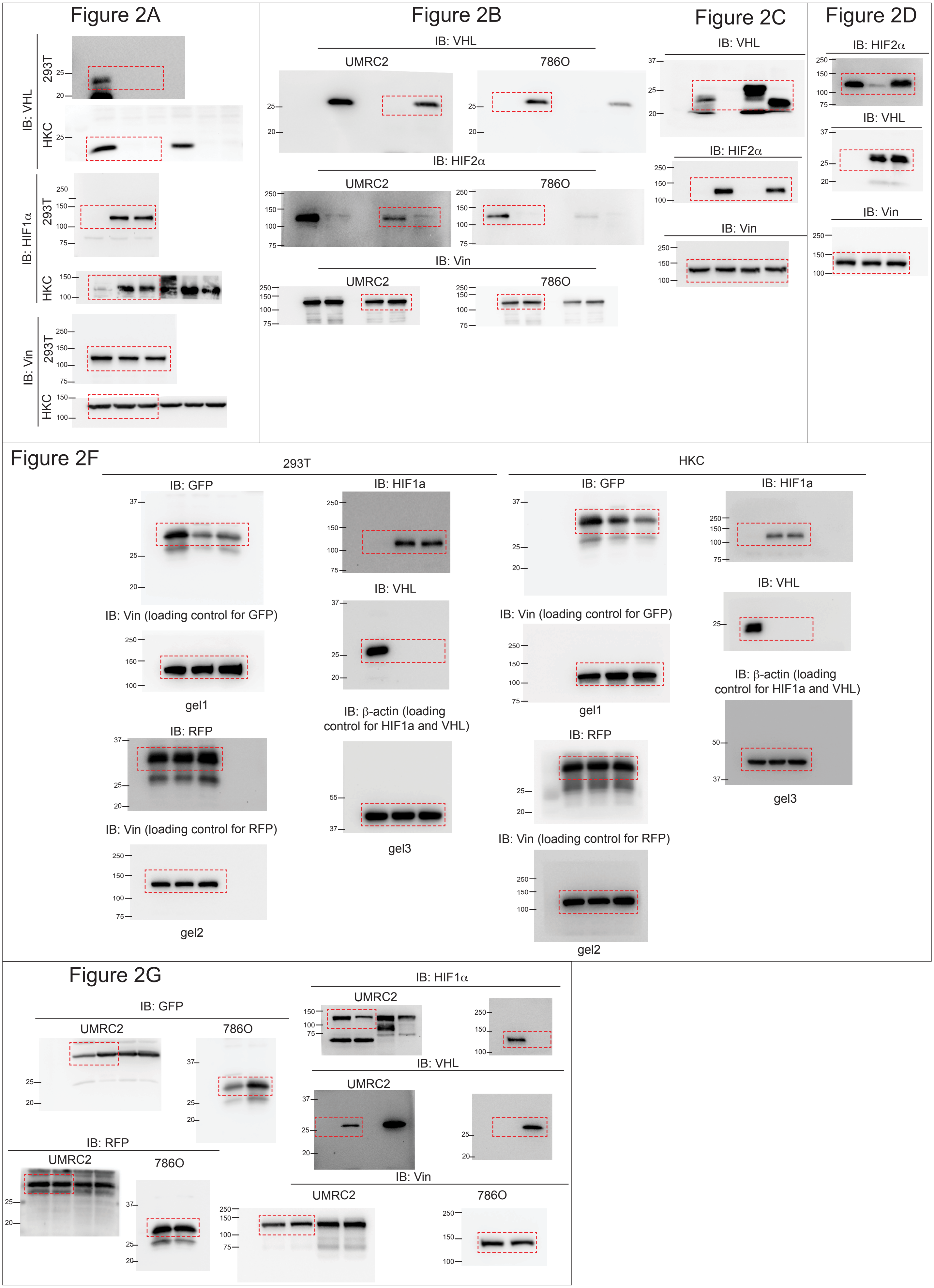

# Full unedited gel for Figure 3

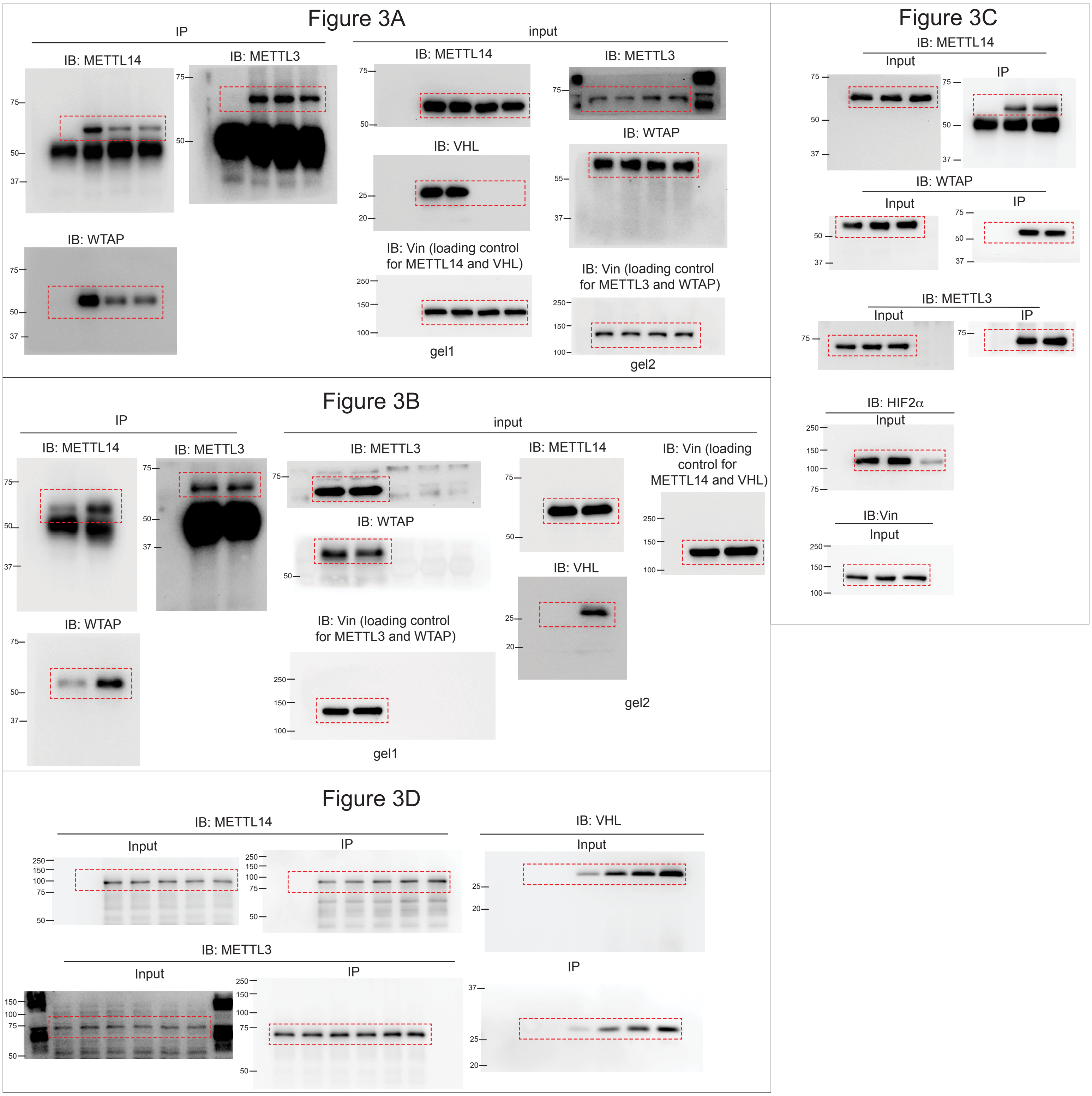

# Full unedited gel for Figure 7

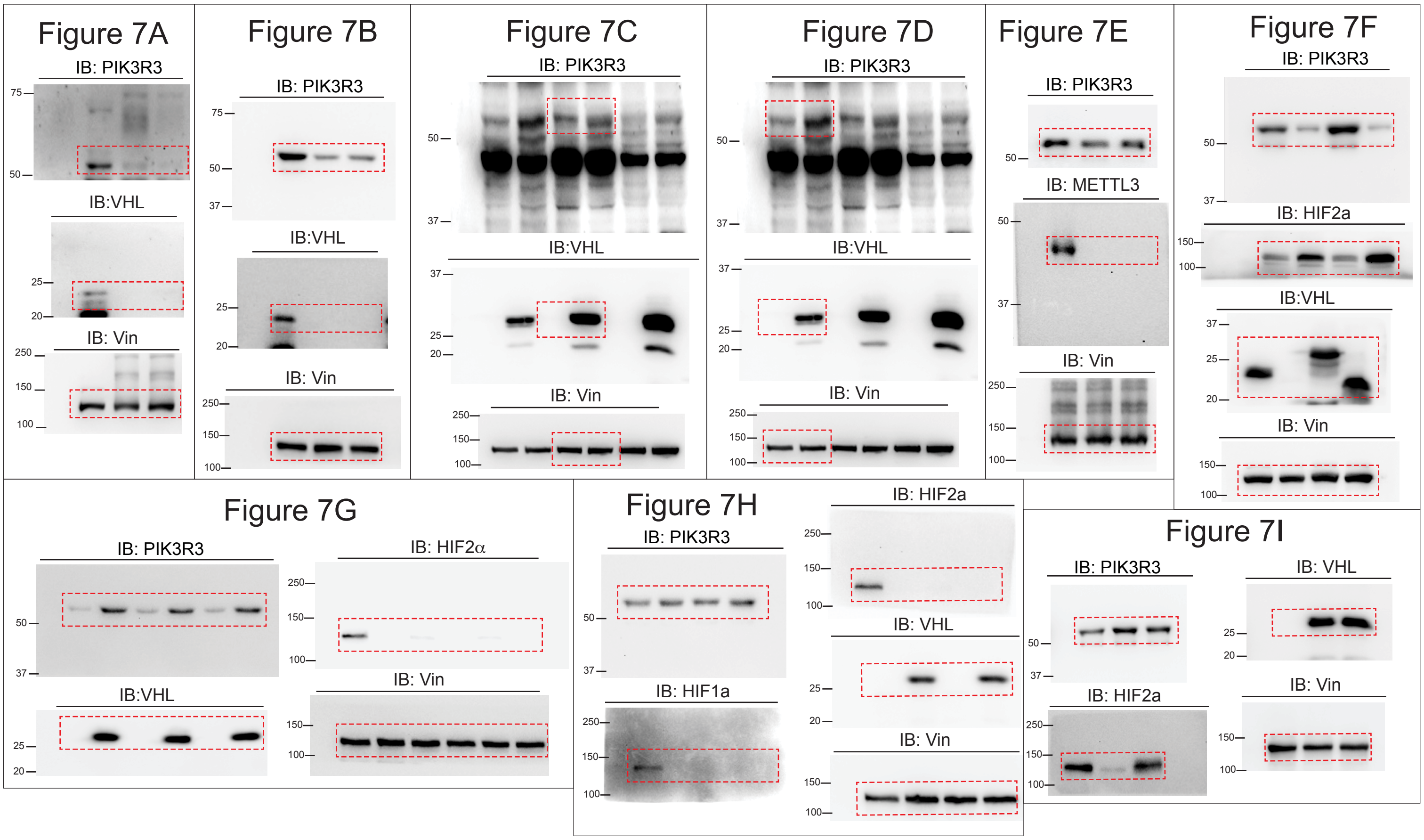


# Full unedited gel for Figure 8

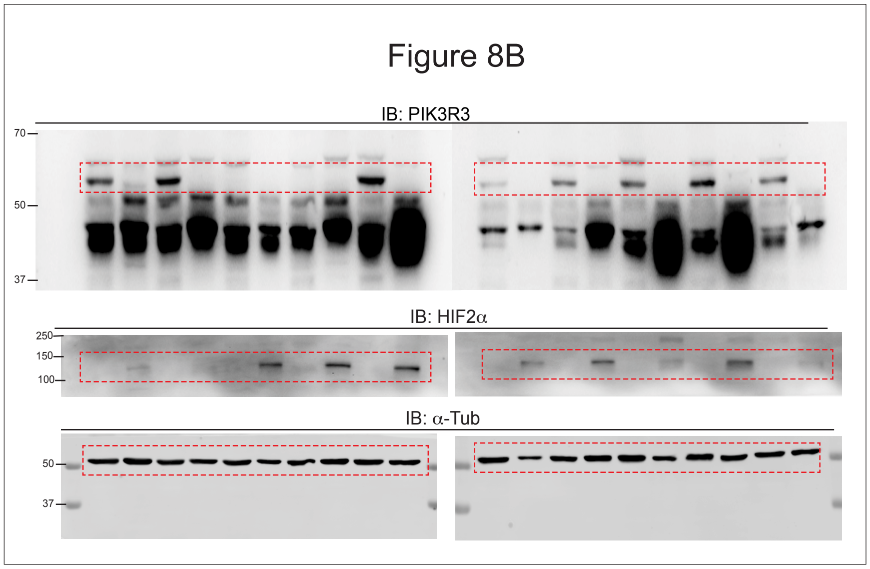


# Full unedited gel for Figure 9

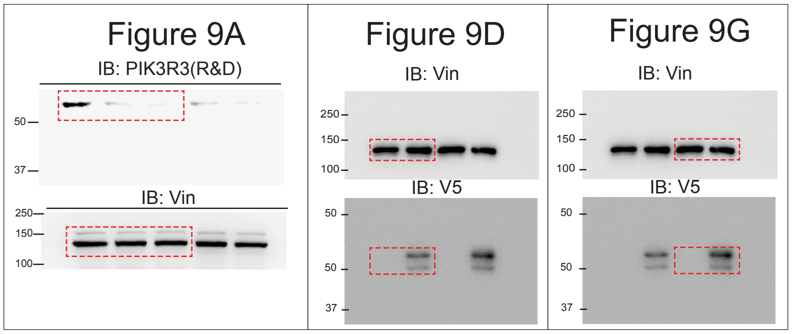

# Full unedited gel for Figure 11

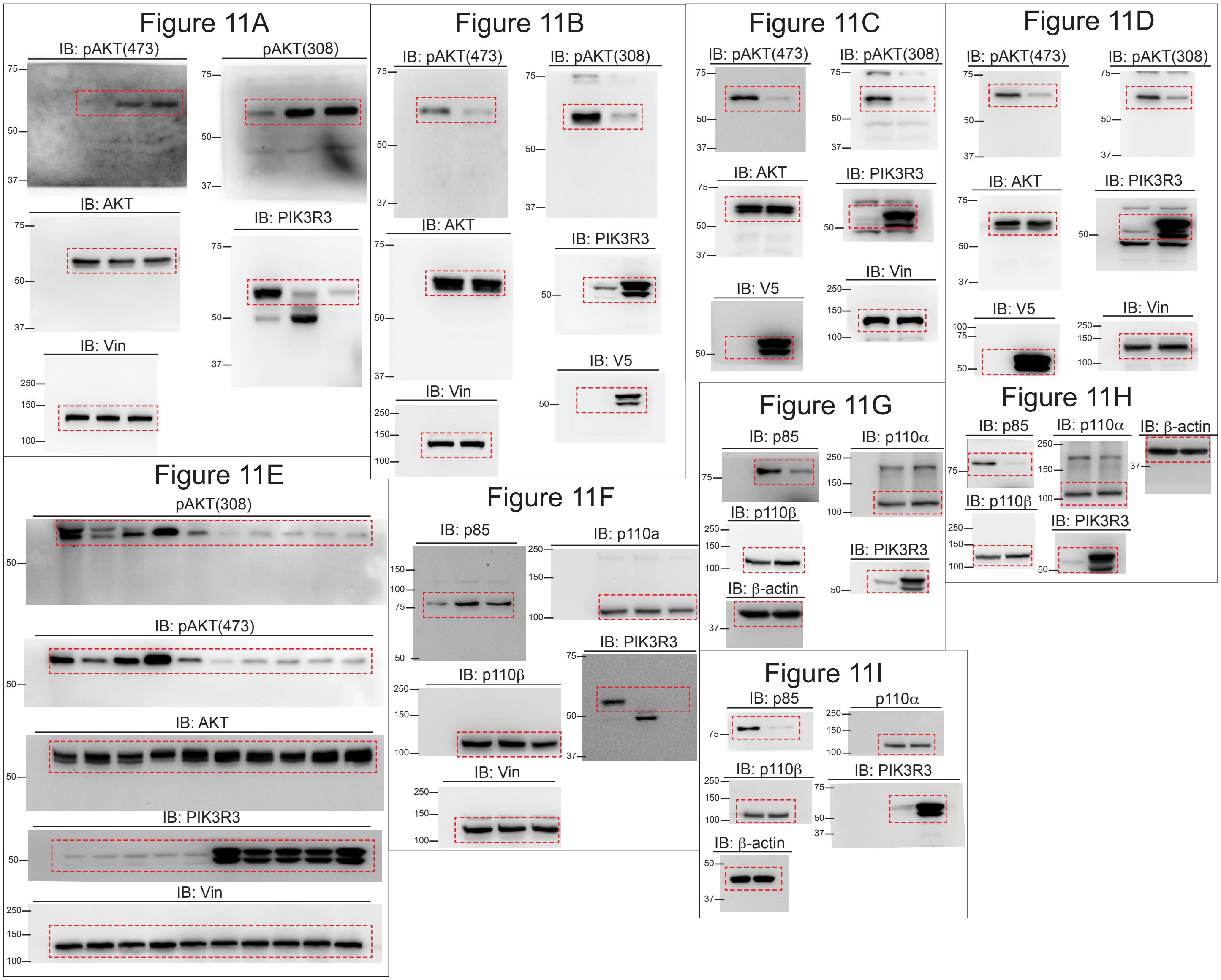

Full unedited gel for Figure 12

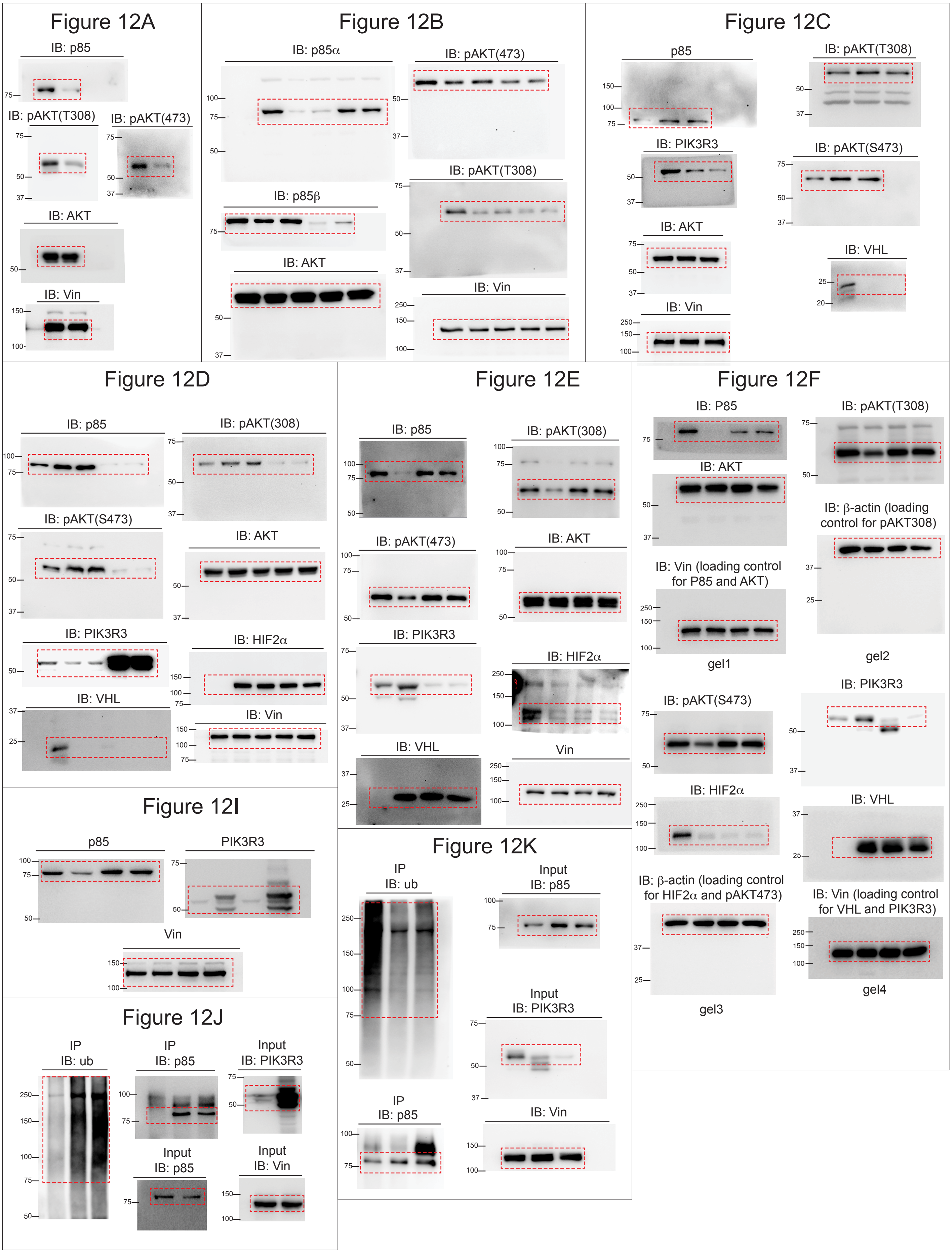

# Full unedited gel for Figure S1

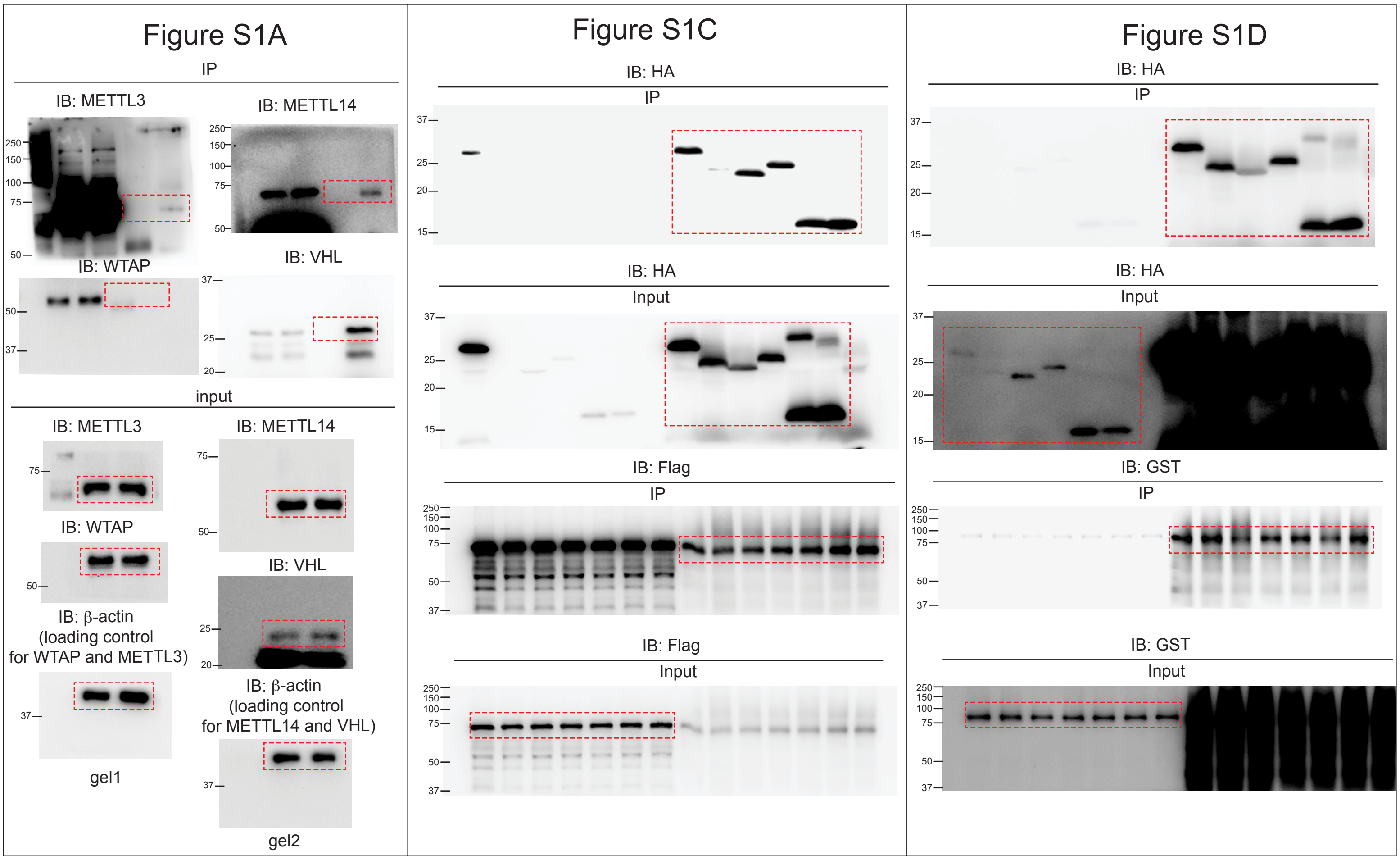


# Full unedited gel for Figure S2

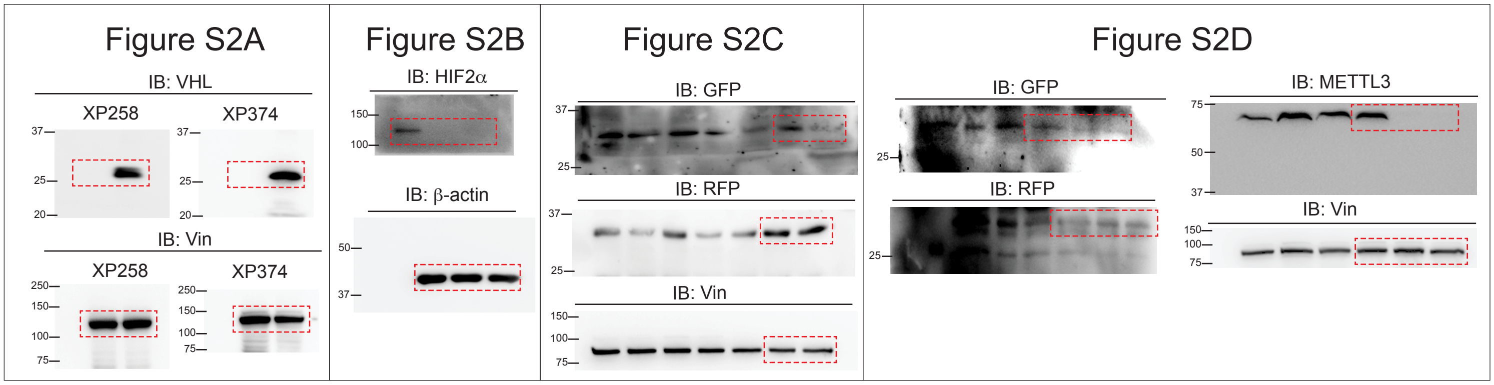

# Full unedited gel for Figure S3

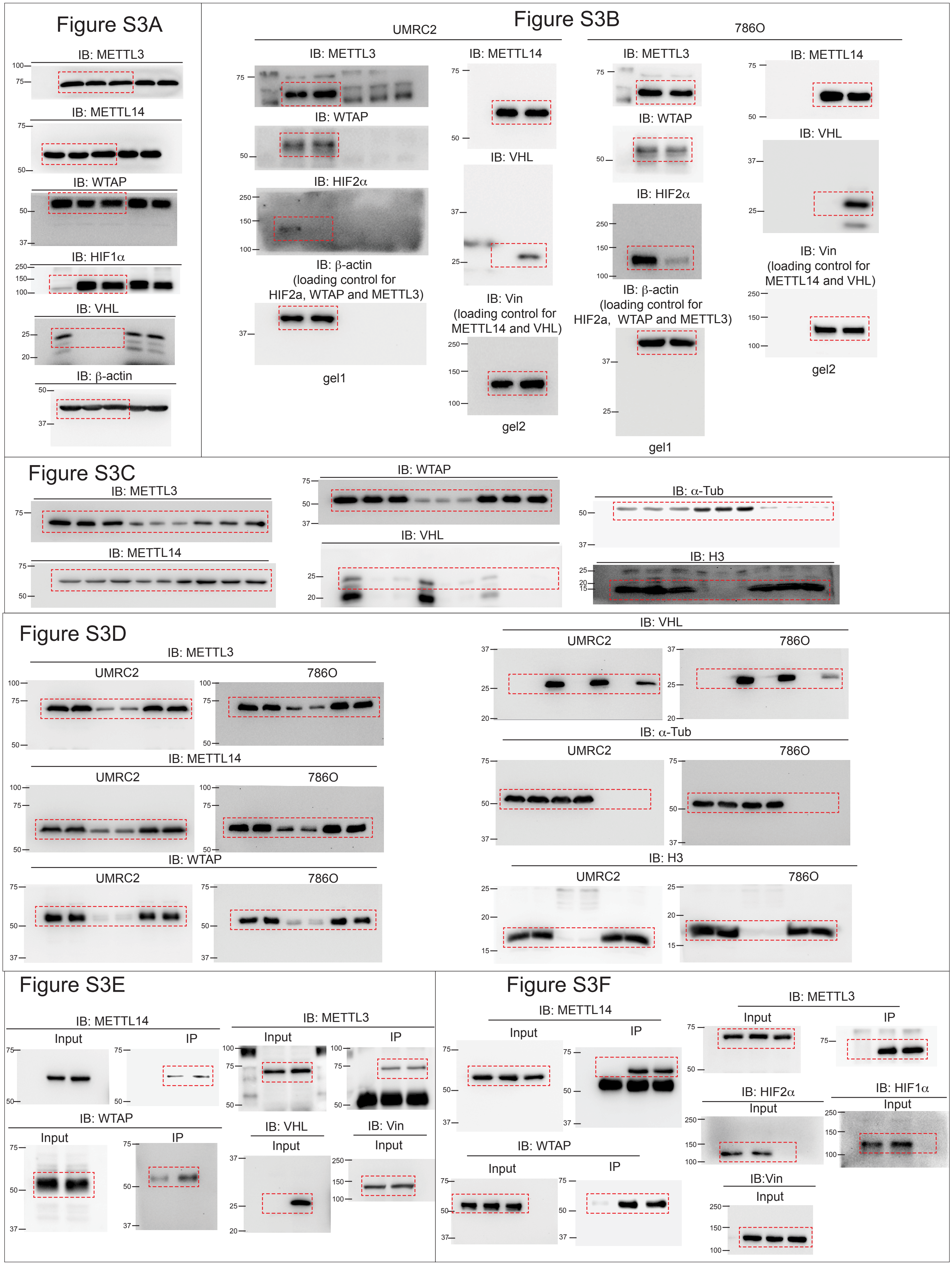

# Full unedited gel for Figure S6

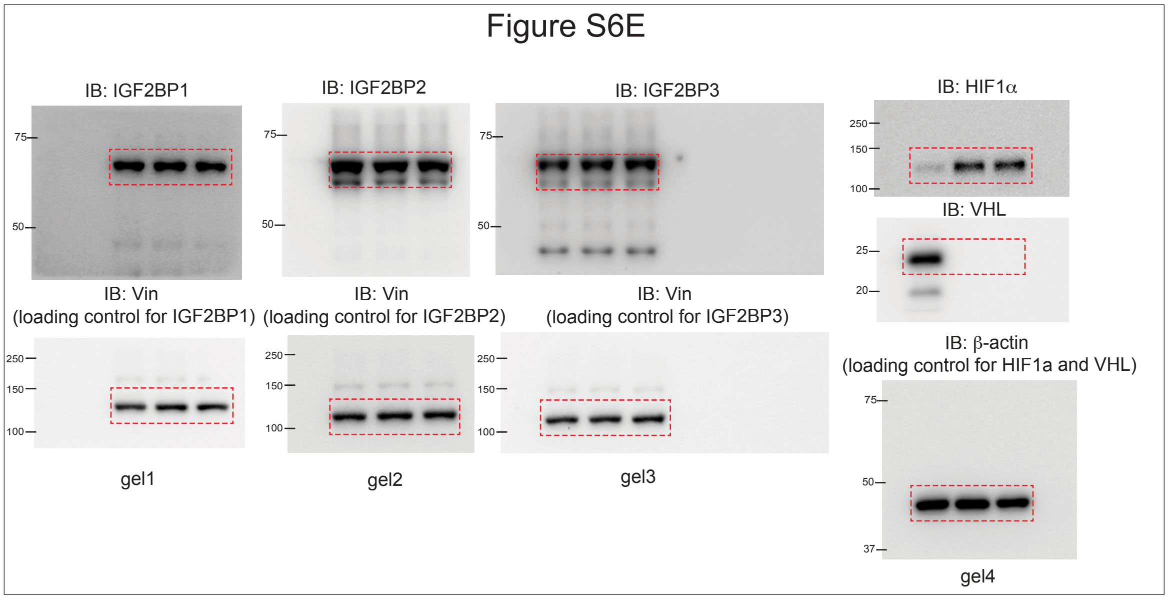


# Full unedited gel for Figure S7

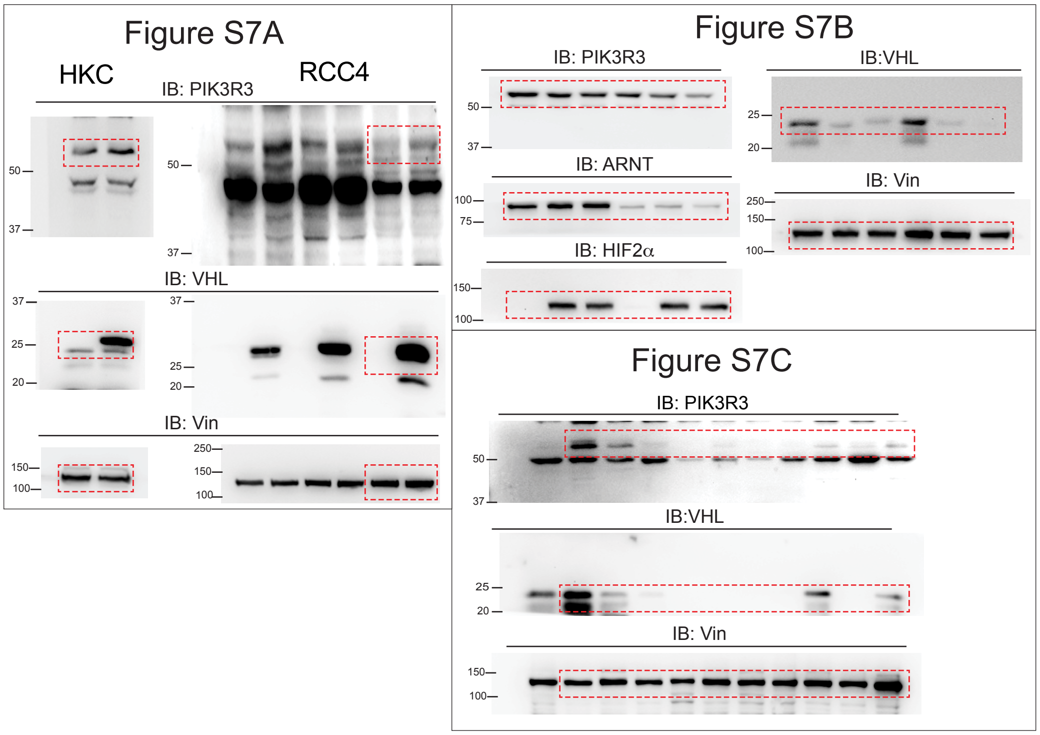

# Full unedited gel for Figure S11

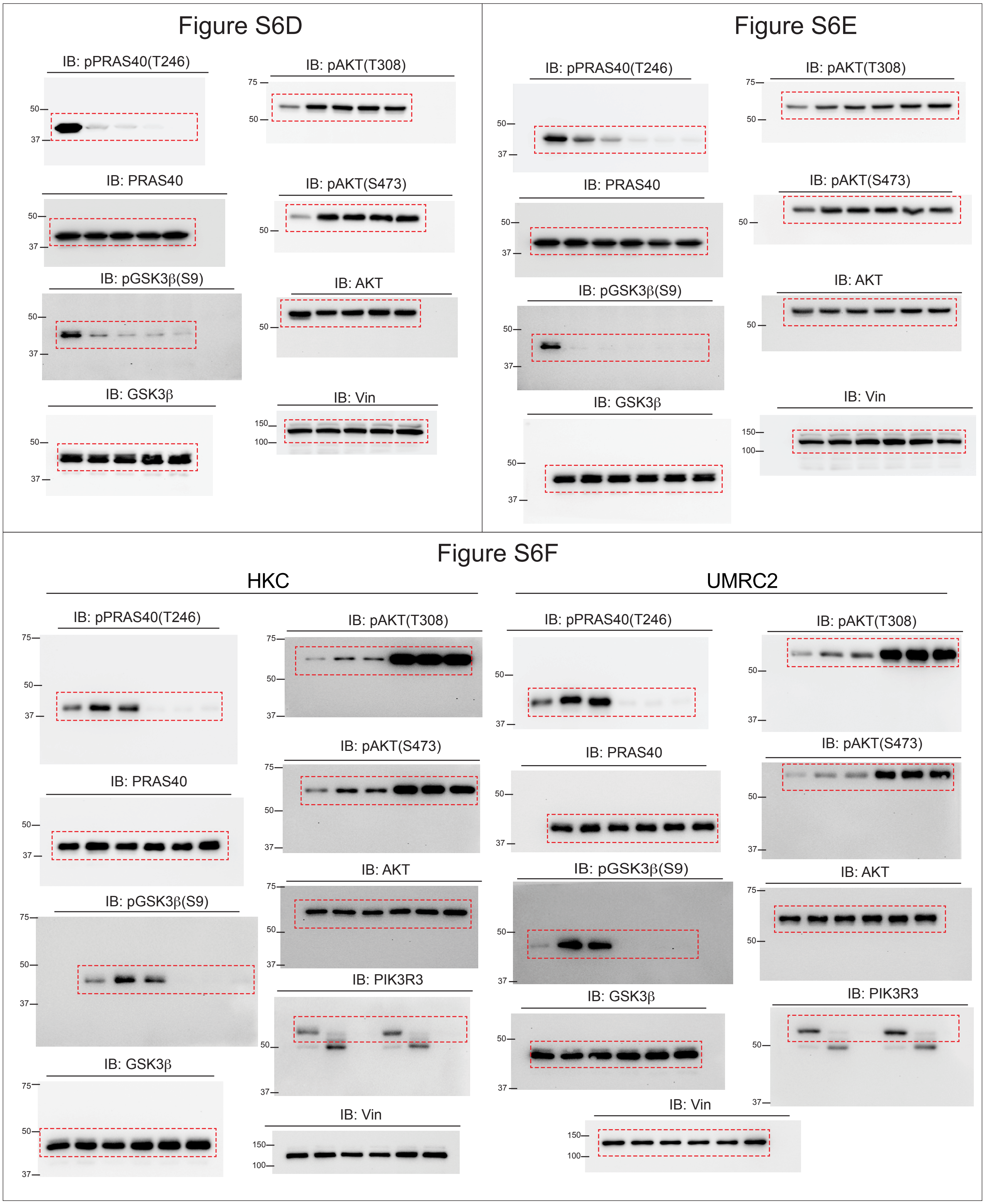

Supplement: Unedited blot and gel images [file jci-134-175703-s014.pdf]
